# An updated checklist of the ants of India with their specific distributions in Indian states (Hymenoptera, Formicidae)

**DOI:** 10.3897/zookeys.551.6767

**Published:** 2016-01-11

**Authors:** Himender Bharti, Benoit Guénard, Meenakshi Bharti, Evan P. Economo

**Affiliations:** 1 Department of Zoology and Environmental Sciences, Punjabi University, Patiala, Punjab, India; 2 School of Biological Sciences, Kadoorie Biological Sciences Building, The University of Hong Kong, Pok Fu Lam Road, Hong Kong SAR, China; 3 Okinawa Institute of Science and Technology Graduate University, Onna, Okinawa, Japan 904-0495

**Keywords:** Checklist, Indian Ants, Formicidae

## Abstract

As one of the 17 megadiverse countries of the world and with four biodiversity hotspots represented in its borders, India is home to an impressive diversity of life forms. However, much work remains to document and catalogue the species of India and their geographic distributions, especially for diverse invertebrate groups. In the present study, a comprehensive and critical list of Indian ant species is provided with up-to-date state-wise distribution. A total of 828 valid species and subspecies names belonging to 100 genera are listed from India. Potential erroneous data, misidentifications and dubious distributional records that may exist in the literature are also identified. The present exhaustive listing of Indian ants will provide a holistic view about diversity and distribution and will also help to identify major undersampled areas where future sampling and taxonomic efforts should be directed.

## Introduction

The Indian subcontinent is well known for its high biodiversity, varied environments and habitats, and interesting geological history. However, much work remains to document and catalogue the species of India and their geographic distributions, especially for diverse invertebrate groups. The country, with a total land area of over 3.2 million km^2^, is positioned on the Indian Plate (the northern portion of Indo-Australian plate) which separated from Gondwanaland during the late Cretaceous, then collided with Eurasia in the Cenozoic (Briggs 2003, Lomolino et al. 2010), although the precise age of this event is still debated (Aitchison et al. 2007). The collision led to the formation of Tibetan Plateau and the Himalaya. The Indian Plate has acted as a vessel carrying fauna and flora from Africa and Madagascar to Eurasia (Briggs 2003). This varied geological history has led to the emergence of a wide diversity of flora and fauna in India, which comprises Malayan, Afrotropical, Mediterranean, central Asian and eastern Palearctic elements.

Most of the country’s land can be assigned to one of two ecozones, the Palaearctic and Indo-Malayan, and 13 terrestrial ecoregions (Olson et al. 2001). The Himalayan system, part of the Palaearctic ecozone, stretches over 3000 kilometers in length, from Myanmar to east of Afghanistan (between longitudes 70E to 100E and latitudes 25N to 40N) and from 80 kilometers to 300 kilometers in width (Bharti 2008). The Himalayas, which form the northern boundary of the country, span across ten states (Jammu & Kashmir, Himachal Pradesh, Uttarakhand, Sikkim, Meghalaya, Tripura, Manipur, Mizoram, Nagaland, Arunachal Pradesh and a little part of Assam and West Bengal). The mountain system extends from east of Brahmaputra to the bend of Indus in the west, but the Himalayan system stretches further from Myanmar to Afghanistan. Kunlun represents the northern extreme of the Himalayan range, followed by the Tibetan plateau. The mountain system meets with high ranges of Central Asia (Hindu Kush, Trans Karakoram, Tian Shan, Kunlun, Trans Alai) forming the Pamir Knot, and Tibet lies to the north-east. The Western Ghats are part of the Western Ghats-Sri Lanka global hotspot, running roughly in a North-south direction for about 1500 kilometres parallel to the coast bordering the Arabian Sea.

Approximately 21% of the country’s landmass is covered by forests (tree canopy density >10%), of which 12% comprises moderately or very dense forests (tree canopy density >40%) (CBD 2014). These include tropical rainforests of the Andaman Islands, the Western Ghats, and Northeast India; coniferous forests of Himalaya; deciduous Sal (*Shorea robusta*) forest of Eastern India; the dry deciduous Teak (various species of *Tectona*) forest of Central and Southern India; and the Babul (*Acacia*) dominated thorn forest of the Central Deccan and Western Gangetic plain (Tritsch 2001). According to the latest estimates (CBD 2014), the country accounts for 7–8% of the total plant and animal species globally recorded, including over 45,000 species of plants and 92,873 species of animals. This included 423 mammalian species (7.81% of Indian total), 1,233 avian species (13.66%), 526 reptilian species (5.7%), 342 amphibian species (5.05%), 3,022 fish species (9.41%) and 63,423 of insects species (6.22%). Out of these, about 4,045 species of flowering plant (angiosperms), 47 species of mammals, 53 species of birds, 156 species of reptiles and 168 species of amphibians are endemic to India (CBD 2014). Most of the endemic taxa listed above are localised in one of the four biodiversity hotspots recognised in India; Himalaya, Indo-Burma, the Western Ghats - Sri Lanka and Sundaland (Nicobar Islands) (Myers et al. 2000 and CBD 2014).

Current data about the diversity and distribution of Indian ants is provided in this work. Ants constitute an important fraction of the animal biomass in terrestrial ecosystems and respond to stress on a much finer scale compared to vertebrates (Hölldobler and Wilson 1990; Andersen 1997). They are widely used to assess landscape disturbance and species diversity (Paknia and Pfeiffer 2011). They perform major ecological functions (predators, scavengers, soil turners, nutrient cyclers, pollinators) and are also responsible for dispersal of numerous plant species (Lach et al. 2010, Del Toro et al. 2012, Guénard 2013). Furthermore, ants mark their presence at almost all levels of terrestrial food webs (Pfeiffer et al. 2013). In this context, knowledge about their diversity and distribution may add to our understanding of their ecological functions, biogeographic patterns and global affinities.

Jerdon (1851, 1854) catalogued the ants of Southern India. Later, Forel (1900a,b, 1901) extended the list by adding 267 new species from the region. Comprehensive documentation of Indian ants was carried by Bingham (1903), who included all the previous works. Later, further contributions were made by various myrmecologists including Forel, Donisthorpe, Emery, Santschi, Mukerjee, Brown, Bolton in terms of descriptions of new taxa (Appendix 1). However, the first ever checklist which cited Indian ants was published by Chapman and Capco (1951; Appendix 1) in their revision of Asian ants. Later efforts to combine knowledge on Indian ants were performed by Guénard and collaborators (Guénard et al. 2010; 2012) in the context of global generic richness and distribution in Asia. In recent years, Bharti and co-workers significantly increased our understanding of ant diversity with both new species descriptions and new distributional records (Appendix 1). This led Bharti (2011) to compile the first modern species checklist inclusive of all earlier records for 652 valid species and subspecies from India and include all the ant records from Himalaya irrespective of its political division. Despite all these efforts, our knowledge about the diversity and distribution of Indian ants remains incomplete and fragmentary, especially on finer geographic scales.

In the present study we provide a comprehensive and critical list of Indian ants with current known state-wise distribution. Our aim is to consolidate previous data, to identify potential erroneous data, misidentifications, dubious distributional records, and more generally to provide a holistic view about the diversity and distribution of Indian ants. This list should also help identify major undersampled areas where future sampling and taxonomic efforts should be directed.

## Methods

Species lists were compiled for 28 Indian states and two union territories (Andaman and Nicobar Islands and Delhi). Data from Union territories of Chandigarh were merged with Punjab, Dadra and Nagar Haveli with Maharashtra, Daman and Diu with Goa, Lakshadweep with Kerala, and data for the state of Telangana were merged with Andhra Pradesh. These lists have been generated based on the literature review of published material, physical examination of material lying in Natural History Museum, London; Indian depositories; personal collection of the first author and websites (cited in the reference section). Additionally, the outcome of recent surveys in approximately the last 15 years in the various regions of India have also significantly enriched the data and have added to the much needed distributional data of various species. Morphospecies have not been included in the list and species name validity, authority and spelling conform to Bolton’s (2015) Synopsis of the Formicidae and Catalogue of Ants of the World.

### Misidentifications and dubious/erroneous records

The continuing accumulation of distributional records and knowledge on species habitats facilitates the identification of previously cited erroneous distributions. This is especially facilitated by the compilation of large global databases and visualization tools like Antweb.org and GABI/antmaps.org Some of the material cited in the earlier literature have been found to bear either erroneous data in terms of locality, or erroneously presented from a region (e.g. potential occurrence), as in the latter case the concerned depositories couldn’t verify the existence of such a material in their possession. Additionally, some of the specimens recorded in the old literature do not have specific locality labels, instead bear labels such as “Northwest Provinces”, “Western India”, “Himalaya”, “Panchmarhi Hills”, “India” to mention a few. Furthermore, as commented by Ward (2007), ant taxonomy is a difficult discipline and species definitions change with taxonomic revisions and with more input of material, so some of the records of Indian ants were found to be misidentifications. To minimize further confusion and future “taxonomic noise,” we mark these records as dubious and provide brief explanation about their dubious status.

## Results and discussion

From India, distributional data for 828 species and subspecies is listed, representing 100 genera grouped in 10 subfamilies. In terms of species richness, the subfamily Myrmicinae is the most speciose (354 species, 42.7%), followed by Formicinae (241 species, 29.1%) Ponerinae (111 species, 13.4%), Dorylinae (55 species, 6.6%) and Dolichoderinae (30 species, 3.6%), while the rest of the smaller subfamilies together constitute 4.2% (Pseudomyrmecinae 11 species, Amblyoponinae 10 species, Proceratiinae 6 species, Ectatomminae 5 species and Leptanillinae 4 species). The trend for generic richness is almost the same except for the subfamily Ponerinae which represents a larger percentage of generic richness than Formicinae (Myrmicinae 37.4%, Ponerinae 20.2% and Formicinae 18.2%).

### Species diversity within genera

The most speciose ant genus is *Camponotus* with 83 named species (one tenth of the total known Indian species), followed by *Polyrhachis* (71 species, 8.5%), *Pheidole* (58 species, 7.0%). Other diverse genera include *Tetramorium* and *Crematogaster* (42 and 41 species, each 5.0%), *Leptogenys* (34 species, 4.1%), *Myrmica* (33 species, 4.0%), *Aenictus* (32 species, 3.8%), *Strumigenys* and *Carebara* (24 species each, 2.9%) respectively (Table 1). Above and beyond these ten genera which have wide distribution within India (except *Myrmica*, which is restricted to Himalayan region), a large majority of genera (66) can be at this point perceived as species-poor in India (5 or less species) including 30 monospecific genera in India (Table 1), and inclusive of two monotypic exotic genera *Anoplolepis* and *Paratrechina*.

**Table 1.** Number of named species of ants per genus in India.

Genus name	# Species & subspecies in genus	Genus name	# Species & subspecies in genus
*Camponotus*	83	*Cryptopone*	3
*Polyrhachis*	71	*Discothyrea*	3
*Pheidole*	58	*Harpegnathos*	3
*Tetramorium*	42	*Odontomachus*	3
*Crematogaster*	41	*Paratopula*	3
*Leptogenys*	34	*Philidris*	3
*Myrmica*	33	*Platythyrea*	3
*Aenictus*	32	*Prenolepis*	3
*Carebara*	24	*Stenamma*	3
*Strumigenys*	24	*Vollenhovia*	3
*Monomorium*	20	*Acropyga*	2
*Aphaenogaster*	15	*Dilobocondyla*	2
*Cerapachys*	15	*Echinopla*	2
*Lepisiota*	15	*Leptanilla*	2
*Lasius*	14	*Lordomyrma*	2
*Cardiocondyla*	13	*Mayriella*	2
*Diacamma*	12	*Mesoponera*	2
*Formica*	12	*Parvaponera*	2
*Temnothorax*	12	*Pristomyrmex*	2
*Anochetus*	11	*Probolomyrmex*	2
*Tetraponera*	10	*Pseudoneoponera*	2
*Dolichoderus*	10	*Recurvidris*	2
*Hypoponera*	9	*Solenopsis*	2
*Nylanderia*	9	*Sphinctomyrmex*	2
*Technomyrmex*	9	*Vombisidris*	2
*Plagiolepis*	8	*Anillomyrma*	1
*Lophomyrmex*	7	*Anoplolepis*	1
*Tapinoma*	7	*Bannapone*	1
*Trichomyrmex*	7	*Buniapone*	1
*Bothroponera*	6	*Calyptomyrmex*	1
*Brachyponera*	6	*Centromyrmex*	1
*Dorylus*	6	*Emeryopone*	1
*Ectomomyrmex*	6	*Gauromyrmex*	1
*Meranoplus*	6	*Gesomyrmex*	1
*Pseudolasius*	6	*Indomyrma*	1
*Stigmatomma*	6	*Iridomyrmex*	1
*Cataulacus*	5	*Kartidris*	1
*Gnamptogenys*	5	*Liometopum*	1
*Myrmoteras*	5	*Liomyrmex*	1
*Cataglyphis*	4	*Metapone*	1
*Chronoxenus*	4	*Myopias*	1
*Messor*	4	*Myopopone*	1
*Myrmecina*	4	*Mystrium*	1
*Myrmicaria*	4	*Ochetellus*	1
*Ponera*	4	*Odontoponera*	1
*Oecophylla*	1	*Paratrechina*	1
*Paraparatrechina*	1	*Perissomyrmex*	1
*Prionopelta*	1	*Rhopalomastix*	1
*Proceratium*	1	*Tyrannomyrmex*	1
*Protanilla*	1	*Yavnella*	1

Within India, several genera including *Myrmica*, *Formica*, *Lasius*, *Stenamma*, *Perissomyrmex* and a majority of the species of *Aphaenogaster* and *Temnothorax* are restricted to the Palearctic region of Himalaya (Table 2, Bharti 2008), while the genera *Calyptomyrmex*, *Emeryopone*, *Indomyrma*, *Lordomyrma*, *Myrmoteras*, *Tyrannomyrmex* and *Yavnella* represent tropical elements restricted to Western Ghats, and *Metapone* to Nicobar Islands. Other tropical genera (*Anillomyrma*, *Buniapone*, *Centromyrmex*, *Dilobocondyla*, *Discothyrea*, *Gauromyrmex*, *Gesomyrmex*, *Indomyrma*, *Kartidris*, *Liomyrmex*, *Mayriella*, *Myopopone*, *Odontoponera*, *Oecophylla*, *Paraparatrechina*, *Paratopula*, *Platythyrea*, *Probolomyrmex*, *Rhopalomastix*, *Tyrannomyrmex*, *Vollenhovia* and *Vombisidris*) are represented by one or few species (Table 2).

**Table 2.** Known species and subspecies diversity per genus within the different Indian states considered.

	Andaman & Nicobar Islands	Andhra Pradesh	Arunachal Pradesh	Assam	Bihar	Chhattisgarh	Delhi	Goa	Gujarat	Haryana
*Acropyga*	1		1	1						
*Aenictus*	4		15	11		1	1	1	4	1
*Anillomyrma*					1					
*Anochetus*	1		3	2	1			1	3	1
*Anoplolepis*	1		1	1				1	1	
*Aphaenogaster*	4		7	1				1		
*Bothroponera*		1	3	3	1			3		1
*Brachyponera*	1		2	3						2
*Buniapone*				1						
*Calyptomyrmex*										
*Camponotus*	16	2	28	26	4	1	6	7	5	6
*Cardiocondyla*	1		3	3	1			2	1	1
*Carebara*	2		6	4				1		
*Cataglyphis*			1		1		1		1	2
*Cataulacus*	4		3	3	1			2		2
*Centromyrmex*				1						
*Cerapachys*			3	2				2		1
*Chronoxenus*	1		2	3			1			
*Crematogaster*	5		17	9	1		2	3	4	11
*Cryptopone*	1									
*Diacamma*	3		5	5	1			2		
*Dilobocondyla*										
*Discothyrea*				1						
*Dolichoderus*	1		4	5					1	
*Dorylus*			4	3	1		2		2	3
*Echinopla*	1		1							
*Ectomomyrmex*			3	3						
*Emeryopone*										
*Formica*										
*Gauromyrmex*			1							
*Gesomyrmex*				1						
*Gnamptogenys*	1		3	3						
*Harpegnathos*			1	2				1		
*Hypoponera*			7	4				1	1	
*Indomyrma*										
*Iridomyrmex*				1	1					
*Kartidris*										
*Lasius*			3	1						
*Lepisiota*		2	3	3	1		1	2	2	4
*Leptanilla*										
*Leptogenys*	2		9	13	1	1		2	4	
*Liometopum*			1	1						
*Liomyrmex*	1									
*Lophomyrmex*			6	2						1
*Lordomyrma*										
*Mayriella*			2							
*Meranoplus*			2	2	1		1	1	1	1
*Mesoponera*										
*Messor*										2
*Metapone*	1									
*Monomorium*	4	1	7	7			2	2	2	2
*Myopias*										
*Myopopone*	1		1	1						
*Myrmecina*			1	1						
*Myrmica*			8		1					
*Myrmicaria*			2	2	1			1		
*Myrmoteras*										
*Mystrium*										
*Nylanderia*	3	1	2	1						
*Ochetellus*										1
*Odontomachus*	2		3	3						
*Odontoponera*	1		1	1			1			1
*Oecophylla*	1	1	1	1	1		1	1	1	1
*Paraparatrechina*									1	
*Paratopula*	1									
*Paratrechina*	1		1	1			1	1	1	
*Parvaponera*			1	1						
*Perissomyrmex*										
*Pheidole*	6	1	14	15	2		7	3	4	4
*Philidris*	3		1	1						
*Plagiolepis*			2	1					1	
*Platythyrea*	2		1	1				1		
*Polyrhachis*	18		17	21	1		1	2	3	1
*Ponera*			1							
*Prenolepis*			1	1						1
*Prionopelta*										
*Pristomyrmex*				1						
*Probolomyrmex*										
*Proceratium*			1							
*Protanilla*										
*Pseudolasius*			1							
*Pseudoneoponera*	1		2	2				1		
*Recurvidris*			1	1						
*Rhopalomastix*										
*Solenopsis*	1		1	1	1			1	1	
*Sphinctomyrmex*										
*Stenamma*										
*Stigmatomma*			1							
*Strumigenys*			10	8				3	2	
*Tapinoma*	2		1	1			1	2	1	2
*Technomyrmex*	1		3	3			1		1	3
*Temnothorax*								1		
*Tetramorium*	4		10	9	3	1	2	7	4	4
*Tetraponera*	5	1	5	5	1		2	4	2	3
*Trichomyrmex*	1		4	3	1		2	3	6	5
*Tyrannomyrmex*										
*Vollenhovia*	2									
*Vombisidris*										
*Yavnella*										
# ***Species***	**112**	**10**	**255**	**217**	**29**	**4**	**36**	**66**	**60**	**67**
# ***Genera***	**40**	**8**	**61**	**58**	**23**	**4**	**19**	**32**	**27**	**27**

	Himachal Pradesh	Jammu & Kashmir	Jharkhand	Karnataka	Kerala	Madhya Pradesh	Maharashtra	Manipur	Meghalaya	Mizoram
*Acropyga*	1			2			1	1	1	1
*Aenictus*	12	5		11	10	2	12	5	6	2
*Anillomyrma*										
*Anochetus*	3	4	1	7	3		3	2	2	2
*Anoplolepis*				1	1		1	1	1	1
*Aphaenogaster*	10	8		1		2	2		4	
*Bothroponera*	2	1	1	3	3	1	3	2		2
*Brachyponera*	3	2		2	2	1	2	2	2	2
*Buniapone*									1	
*Calyptomyrmex*					1					
*Camponotus*	18	15	4	18	26	1	19	7	13	2
*Cardiocondyla*	5	2		5	3		5	2	1	2
*Carebara*	8	5		7	8		2	1	3	2
*Cataglyphis*	1	2				2	1		2	
*Cataulacus*	3	1		2	3		2	1	2	1
*Centromyrmex*				1	1					
*Cerapachys*	4	2		3	11		1	1	3	1
*Chronoxenus*	2	3		3	1		3	1	1	
*Crematogaster*	13	10	1	18	11	2	13	9	11	4
*Cryptopone*	1	1			1					
*Diacamma*		1		8	7		3	2	2	2
*Dilobocondyla*	1			1						
*Discothyrea*				1	2					
*Dolichoderus*	1	2		4	1			2	6	1
*Dorylus*	2	2	1	2	1		2	2	1	2
*Echinopla*									1	
*Ectomomyrmex*	1			3	4				3	
*Emeryopone*				1						
*Formica*	10	12				1				
*Gauromyrmex*	1									
*Gesomyrmex*										
*Gnamptogenys*				1	2			2	3	2
*Harpegnathos*	1	1		2	2		2	1	1	
*Hypoponera*	3	4		7	4		3	1		2
*Indomyrma*				1	1					
*Iridomyrmex*			1				1		1	
*Kartidris*									1	
*Lasius*	7	8							1	
*Lepisiota*	12	8	1	6	4	2	6	2	5	1
*Leptanilla*	1				1					
*Leptogenys*	5	3	1	13	17	1	7	3	9	2
*Liometopum*									1	
*Liomyrmex*										
*Lophomyrmex*	4	3		1	1		1		4	1
*Lordomyrma*					2					
*Mayriella*	1	1							1	
*Meranoplus*	1	1	1	2	5		2	2	3	2
*Mesoponera*				1	1		1			
*Messor*	2	3			1	1	1			
*Metapone*										
*Monomorium*	6	6		10	6	1	7	6	5	2
*Myopias*		1								
*Myopopone*										
*Myrmecina*				1	3		1		1	
*Myrmica*	15	20				3			3	
*Myrmicaria*	1	1	1	2	1		1	1	1	
*Myrmoteras*				1	5					
*Mystrium*										
*Nylanderia*	6	4		2	3		2	1		
*Ochetellus*	1			1			1			
*Odontomachus*	1	2		1	1			1	3	
*Odontoponera*	1	1		1	1				1	
*Oecophylla*	1	1	1	1	1	1	1	1	1	1
*Paraparatrechina*	1	1								
*Paratopula*				1	1					
*Paratrechina*	1	1		1	1		1	1	1	1
*Parvaponera*				1	1					
*Perissomyrmex*										
*Pheidole*	15	16	2	15	13		25	3	16	2
*Philidris*										
*Plagiolepis*	2	3		4	1		2		1	
*Platythyrea*	2	1		2	1		1			
*Polyrhachis*	10	5		25	23		9	4	19	2
*Ponera*	2									
*Prenolepis*	1	1						1		
*Prionopelta*	1	1								
*Pristomyrmex*										
*Probolomyrmex*				1						
*Proceratium*	1								1	
*Protanilla*					1					
*Pseudolasius*	3	2							1	
*Pseudoneoponera*	2	2		1	1		1	1	1	1
*Recurvidris*	1	1		1	1		1	1	1	
*Rhopalomastix*				1						
*Solenopsis*	1	1	1	2	2		1	1	1	1
*Sphinctomyrmex*					1					
*Stenamma*	3	1								
*Stigmatomma*	1			2	2					
*Strumigenys*	5	1		6	14		4	2	4	3
*Tapinoma*	2	3		3	2		2	2	2	1
*Technomyrmex*	3	1		5	4		2	3	1	
*Temnothorax*	10	7		1	1	2			1	
*Tetramorium*	12	5	2	15	22	1	9	4	12	2
*Tetraponera*	3	3	1	4	6	1	5	2	4	1
*Trichomyrmex*	5	4	1	6	5	1	6		1	1
*Tyrannomyrmex*					1					
*Vollenhovia*	1									
*Vombisidris*				2	1					
*Yavnella*					1					
# ***Species***	**259**	**206**	**21**	**257**	**268**	**26**	**181**	**87**	**178**	**55**
# ***Genera***	**63**	**55**	**16**	**61**	**63**	**18**	**46**	**39**	**54**	**33**

	Nagaland	Orissa	Punjab	Rajasthan	Sikkim	Tamil Nadu	Tripura	Uttar Pradesh	Uttarakhand	West Bengal
*Acropyga*	1	1			1				1	1
*Aenictus*	2		3	1	11	5	1	4	4	16
*Anillomyrma*										
*Anochetus*	1	2	1	3	3	6		2	2	5
*Anoplolepis*	1	1	1		1	1	1			1
*Aphaenogaster*					7	1	1		6	7
*Bothroponera*	1	2	1		3	6	2	1	1	6
*Brachyponera*	2	1	3		3	1	2	1	3	4
*Buniapone*					1				1	
*Calyptomyrmex*										
*Camponotus*	4	11	9	9	27	17	4	3	15	47
*Cardiocondyla*	3		1		4	2	1	1	2	5
*Carebara*		4	4	1	5	2		1	4	10
*Cataglyphis*			2	2	1	2		2	1	1
*Cataulacus*	2	1			2	3			2	2
*Centromyrmex*		1								1
*Cerapachys*	2	1	2	1	3	2	1	1	4	5
*Chronoxenus*	1	1	2		4	1			1	4
*Crematogaster*	6	5	8	2	20	14	2	3	8	28
*Cryptopone*						1				1
*Diacamma*	1	2			6	5	2			6
*Dilobocondyla*					1				1	
*Discothyrea*					1					
*Dolichoderus*	1				9		1		1	4
*Dorylus*	2	2	2	2	3	3		2	2	5
*Echinopla*										
*Ectomomyrmex*					3	2	1		1	4
*Emeryopone*										
*Formica*					1				3	
*Gauromyrmex*					1					
*Gesomyrmex*										
*Gnamptogenys*					2	1				3
*Harpegnathos*	1		2		1	2			1	2
*Hypoponera*	2	1	2		5	2			2	7
*Indomyrma*										
*Iridomyrmex*		1			1	1				1
*Kartidris*					1					1
*Lasius*					5				3	5
*Lepisiota*		2	7	2	4	2		1	9	11
*Leptanilla*						1				
*Leptogenys*	1	4	1	1	13	8	4	2	4	21
*Liometopum*										
*Liomyrmex*										1
*Lophomyrmex*		1			4	1		1	3	4
*Lordomyrma*										
*Mayriella*					1				1	1
*Meranoplus*	1	1	1	1	2	4	2	1	1	4
*Mesoponera*										1
*Messor*			2	2				1		
*Metapone*										
*Monomorium*	2	6	3	6	7	9	2	4	4	11
*Myopias*										
*Myopopone*					1					
*Myrmecina*		1			1	1				1
*Myrmica*					8				3	8
*Myrmicaria*			1		2	2			1	3
*Myrmoteras*						2				
*Mystrium*						1			1	
*Nylanderia*		1	1	1	3	3			4	6
*Ochetellus*									1	
*Odontomachus*	1				2					2
*Odontoponera*	1		1		1			1	1	1
*Oecophylla*	1	1	1	1	1	1	1	1	1	1
*Paraparatrechina*					1					1
*Paratopula*		1						1		1
*Paratrechina*	1	1	1	1	1	1	1	1	1	1
*Parvaponera*			1		1	1				1
*Perissomyrmex*										1
*Pheidole*	3	6	6	2	17	16	3	5	8	27
*Philidris*					1					1
*Plagiolepis*			1	1	4				1	5
*Platythyrea*			1		1	1			1	2
*Polyrhachis*	1	4	3	1	12	13	5	1	6	31
*Ponera*					2				1	
*Prenolepis*			1						2	
*Prionopelta*										
*Pristomyrmex*					1					
*Probolomyrmex*						2				
*Proceratium*					1			1	1	1
*Protanilla*										
*Pseudolasius*					3				1	
*Pseudoneoponera*	1	1	2		1	1	1		2	2
*Recurvidris*		1	1		1	1		1	1	1
*Rhopalomastix*					1					1
*Solenopsis*	1	1	1	1	1	1	1			1
*Sphinctomyrmex*		1			1	1				1
*Stenamma*										
*Stigmatomma*					2	2				3
*Strumigenys*	2				11	4	1	2	3	9
*Tapinoma*	1	2	2	1	1	1	1	2	1	3
*Technomyrmex*	1	1	1		4	3		1	2	3
*Temnothorax*					1				2	
*Tetramorium*	2	2	7	3	14	10	1	1	6	16
*Tetraponera*	3	3	4	1	4	6	1	2	3	6
*Trichomyrmex*		1	5	6	3	5		2	4	5
*Tyrannomyrmex*										
*Vollenhovia*										
*Vombisidris*										
*Yavnella*										
# ***Species***	**56**	**79**	**98**	**52**	**276**	**184**	**43**	**53**	**149**	**382**
# ***Genera***	**33**	**37**	**39**	**24**	**69**	**51**	**25**	**31**	**54**	**65**

Despite including nearly a third of the global ant generic richness (100/323), no genera are known to be endemic to India.

### Regional diversity, endemism and exotic species

Two biogeographically significant regions of India, Himalaya and Western Ghats harbour a large number of ant species. 656 species from 88 genera were recorded from Himalaya, and 455 species from 75 genera were recorded from the Western Ghats.

From a total 828 species, 256 species (31%) we considered endemic to India and approximately 71% of these endemics are exclusively concentrated in two of the above listed biodiversity hotspots. Although we feel that some of the Indian states are underrepresented in the existing data due to inadequacy of surveys, based on the currently available data the state of West Bengal has the highest number of species (382) representing 65 genera followed by state of Sikkim with 276 species representing 69 genera.

The endemism of Indian ants (31%) is much higher than for birds (4.3%), fishes (8%), angiosperms (10%) or mammals (11%), lower than amphibians (49%) and most similar to reptiles (29%) (CBD 2014). With nearly one of three ant species known to be endemic to India, more conservation efforts should be directed to this group to evaluate the distribution and ecology of these species and evaluate the potential threat that some of these species might already experience.

### Undersampling and future directions

With 828 species recorded, India represents one of the richest countries in the Indo-Malayan region. India remains less diverse than China with over 950 species recorded (Guénard and Dunn 2012; Liu et al. 2015), but similar to the island of Borneo (Pfeiffer et al. 2011, http://antmaps.org) and more diverse than the Philippines (General and Alpert 2012, http://antmaps.org). However, considering the high number of species recently described from India by Bharti & co-workers (Appendix 1) and the lack of knowledge for a large part of India (Figure 2), there is little doubt the number of ant species reported from India should keep increasing in the forseeable future.

**Figure 1.**
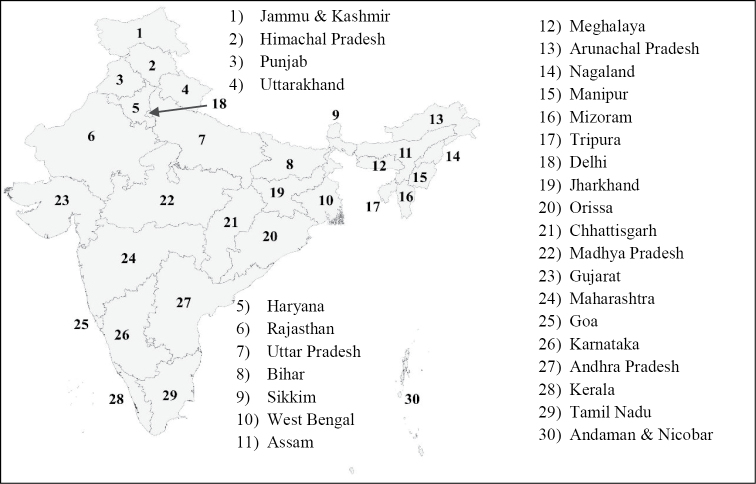
List and geographic position of Indian states considered in this study

**Figure 2.**
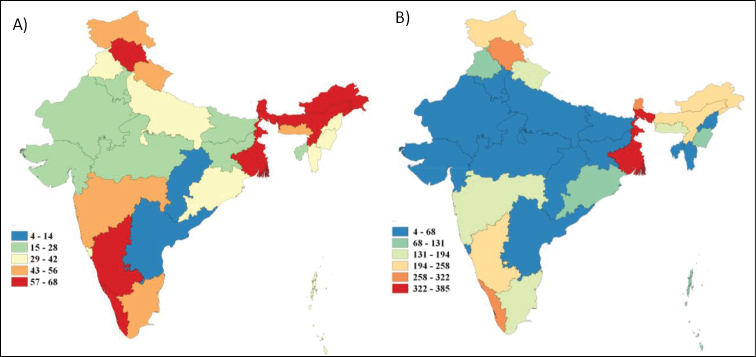
Generic (**A**) and species (**B**) richness based on nominal species for the different states of India.

We present, for the first time, patterns of species richness for the different Indian states, and thereby provide a more detailed biogeographic picture of ant richness. Above all, these results reveal large areas lacking surveys and/or taxonomic resolution on the local myrmecofauna (Figure 2A, B). Our results indicate that no less than seventeen of the thirty administrative regions studied (57%) have fewer than 100 species recorded. In comparison, this roughly corresponds to the species richness observed in countries located in cooler-temperate regions like Poland (Czechowski et al. 2012) or North Korea (Radchenko 2005). Considering the geographic position of Indian states and their climatic ranges (Attri and Tyagi 2010), with most of these regions located in humid subtropical, wet and dry tropical or semi-arid climates, the faunal richness for these regions should be much higher than our current data indicate. For example, the small, Himalayan state of Sikkim (278 species) has higher recorded richness than the tropical moist region of Kerala (259 species). Similarly, regions of the northeastern part of India bordering Myanmar have highly diverse tropical moist forest ecosystems, but only have sparse records for ants.

These gaps in our current knowledge underscores the need for vigorous sampling and identification programs to target specific regions of India. Moreover, due to this incomplete knowledge, the true richness of the Indian ant fauna cannot be assessed with high confidence at this time. However, in light of the undersampling of most regions of the country, we expect the true richness to be greater than 1000 species.

### Non-native species

Among the species present in India, 24 species are considered here as non-native (see Table 3), although the exact origin of a few other species is still uncertain and thus could be included (or removed) if more targeted future studies are conducted. Among the exotic species, several are known for their invasive ecological characteristics: *Anoplolepis gracilipes* (Smith), *Paratrechina longicornis* (Latreille), and *Pheidole megacephala* (Fabricius). The ecological impacts of these species in India have not been studied to date. Furthermore, this list could, and likely will, expand in the future with new arrivals. In particular, several damaging species including *Solenopsis invicta* Buren and *Wasmannia auropunctata* (Roger) are already widespread in tropical and subtropical parts of Asia and with no doubt could find suitable habitats within the diversity of Indian ecosystems if given the opportunity. Both prevention and control measures would be highly adviseable to protect Indian ecosystems and economic interests from the arrival of invasive species.

**Table 3.** List of exotic ants in India

**Formicinae**:
*Anoplolepis gracilipes*
*Nylanderia vividula*
*Paratrechina longicornis*
*Plagiolepis alluaudi*
**Myrmicinae**:
*Cardiocondyla emeryi*
*Cardiocondyla mauritanica*
*Monomorium carbonarium*
*Monomorium monomorium*
*Monomorium pharaonis*
*Monomorium subopacum*
*Pheidole megacephala*
*Solenopsis geminata*
*Strumigenys emmae*
*Strumigenys membranifera*
*Strumigenys rogeri*
*Tetramorium bicarinatum*
*Tetramorium caldarium*
*Tetramorium pacificum*
*Tetramorium simillimum*
*Tetramorium tonganum*
*Trichomyrmex destructor*
**Ponerinae**:
*Brachyponera sennaarensis*
*Hypoponera ragusai*
*Leptogenys falcigera*

## Conclusion

As some of the states/regions are clearly undersampled, future explorations will reveal more species diversity of ants from India. Similarly, upcoming taxonomic revisions will redefine species boundaries, species distributions and affinities with adjoining biogeographic regions. Consequently, the data presented here marks a waypoint in the effort towards elucidating the regional diversity and distribution of Indian ants. In light of the ecological importance of ants in most terrestrial ecosystems, the relatively poor available knowledge of ants in most Indian states, and the high level of endemism of Indian ants, we encourage urgent, large-scale, and sustained efforts to monitor, characterize, and conserve the Indian myrmecofauna.

### Species list

**Table:** List of species of India with their known distribution in India states sorted by subfamily. Number in parentheses cite the source for each record and are presented in the Appendix 1. (I): Introduced species, (E) Endemic species to India.

Taxonomy	State records
**AMBLYOPONINAE**	
***Bannapone***	
*Bannapone pertinax* (Baroni Urbani, 1978) **(E)**	Sikkim (1), West Bengal (1)
***Myopopone***	
*Myopopone castanea* (Smith,1860)	Andaman and Nicobar Islands (105, 160, 189, 206, 254, 355), Arunachal Pradesh (1), Assam (382), Sikkim (7, 105, 114, 160, 206, 355)
***Mystrium***	
*Mystrium camillae* Emery, 1889	Tamil Nadu (7, 402), Uttarakhand (1)
***Prionopelta***	
*Prionopelta kraepelini* Forel, 1905	Himachal Pradesh (37), Jammu & Kashmir (37)
***Stigmatomma***	
*Stigmatomma awa* (Xu & Chu, 2012)	Arunachal Pradesh (1)
*Stigmatomma bellii* (Forel, 1900)	Karnataka (7, 19, 20, 105, 114, 160, 179, 261, 352), Kerala (1), Tamil Nadu (7), West Bengal (352)
*Stigmatomma boltoni* (Bharti & Wachkoo, 2011) **(E)**	Himachal Pradesh (7, 36)
*Stigmatomma minutum* Forel, 1913	Kerala (1), Tamil Nadu (7)
*Stigmatomma rothneyi* (Forel, 1900)	Karnataka (261), Sikkim (1), West Bengal (1)
*Stigmatomma xui* Bharti & Rilta, 2015	Sikkim (413)
**DOLICHODERINAE**	
***Chronoxenus***	
*Chronoxenus dalyi* (Forel, 1895)	Andaman and Nicobar Islands (254), Arunachal Pradesh (1), Assam (1), Jammu & Kashmir (348), Karnataka (178, 315), Maharashtra (178, 315), Sikkim (1), Tamil Nadu (7, 12, 178, 315, 352, 391), West Bengal (1)
*Chronoxenus myops* (Forel, 1895)	Assam (12, 315), Delhi (1), Himachal Pradesh (178, 315), Jammu & Kashmir (80), Karnataka (7, 178), Maharashtra (178, 315), Meghalaya (1), Punjab (79), Sikkim (1), West Bengal (1)
*Chronoxenus walshi* (Forel, 1895)	Kerala (1), Orissa (7, 12, 178, 315), Sikkim (1), West Bengal (1)
*Chronoxenus wroughtonii* (Forel, 1895)	Arunachal Pradesh (1), Assam (1), Himachal Pradesh (7, 178, 315), Jammu & Kashmir (1), Karnataka (7, 12, 178, 287, 315), Maharashtra (178, 315), Manipur (1), Nagaland (1), Punjab (79), Sikkim (1), Uttarakhand (1), West Bengal (1)
***Dolichoderus***	
*Dolichoderus affinis* Emery, 1889	Assam (248, 249, 355), Karnataka (362), Manipur (131, 244), Meghalaya (248, 249, 355), Sikkim (131, 355), West Bengal (1)
*Dolichoderus affinis glabripes* Forel, 1895	Assam (12, 131, 315), Meghalaya (1), Sikkim (1)
*Dolichoderus feae* Emery, 1889	Arunachal Pradesh (1), Manipur (131, 244), Meghalaya (1), Sikkim (1)
*Dolichoderus moggridgei* Forel, 1886	Assam (114, 131, 161, 172, 178, 315, 355), Sikkim (1)
*Dolichoderus moggridgei bicolor* Santschi, 1920 **(E)**	Sikkim (1)
*Dolichoderus moggridgei lugubris* Santschi, 1920 **(E)**	Sikkim (1)
*Dolichoderus sundari* Mathew & Tiwari, 2000 **(E)**	Arunachal Pradesh (1), Meghalaya (1)
*Dolichoderus taprobanae* (Smith, 1858)	Andaman and Nicobar Islands (254), Arunachal Pradesh (1), Assam (1), Himachal Pradesh (1), Jammu & Kashmir (80), Karnataka (178, 262), Kerala (178), Meghalaya (1), Mizoram (1), Nagaland (1), Sikkim (1), Tripura (250), Uttarakhand (1), West Bengal (1)
*Dolichoderus taprobanae gracilipes* (Mayr, 1879)	Karnataka (178), Sikkim (1), West Bengal (1)
*Dolichoderus thoracicus* (Smith, 1860)	Arunachal Pradesh (1), Assam (1), Gujarat (340), Jammu & Kashmir (80), Karnataka (178, 262, 340), Meghalaya (1), Sikkim (1), West Bengal (1)
***Iridomyrmex***	
*Iridomyrmex anceps* (Roger, 1863)	Assam (178, 207, 249, 315), Bihar (7, 122, 207, 214), Jharkhand (7, 122, 207, 214), Maharashtra (115), Meghalaya (1), Orissa (415), Sikkim (1), Tamil Nadu (7, 122, 207), West Bengal (7, 122, 207, 300, 356)
***Liometopum***	
*Liometopum lindgreeni* Forel,1902	Arunachal Pradesh (1), Assam (1), Meghalaya (1)
***Ochetellus***	
*Ochetellus glaber* (Mayr, 1862)	Haryana (23), Himachal Pradesh (23), Karnataka (178), Maharashtra (178), Uttarakhand (1)
***Philidris***	
*Philidris laevigata* (Emery, 1895)	Andaman and Nicobar Islands (254), Arunachal Pradesh (1), Assam (1), Sikkim (1), West Bengal (1)
*Philidris myrmecodiae* (Emery, 1887)	Andaman and Nicobar Islands (254)
*Philidris myrmecodiae andamanensis* (Forel, 1903) **(E)**	Andaman and Nicobar Islands (12, 161, 189, 254, 315)
***Tapinoma***	
*Tapinoma andamanense* Forel, 1903 **(E)**	Andaman and Nicobar Islands (114, 161, 189, 254, 315)
*Tapinoma annandalei* (Wheeler, 1928)	Orissa (114, 315, 389)
*Tapinoma himalaica* Bharti, Kumar & Dubovikoff, 2013 **(E)**	Himachal Pradesh (1), Jammu & Kashmir (77), Punjab (77)
*Tapinoma indicum* Forel, 1895	Goa (410), Karnataka (7, 122, 287), Kerala (7, 225), Maharashtra (115, 178, 214, 315, 383, 391), Manipur (244), Meghalaya (1), Uttar Pradesh (214), West Bengal (204)
*Tapinoma luff*ae (Kurian, 1955)	India (no state record, 32)
*Tapinoma melanocephalum* (Fabricius, 1793)	Andaman and Nicobar Islands (254, 257), Arunachal Pradesh (1), Assam (1), Delhi (1), Goa (7, 410, 411, 412), Gujarat (178, 335, 337, 338, 340, 342, 344), Haryana (335, 337, 340), Himachal Pradesh (342), Jammu & Kashmir (67, 80), Karnataka (7, 124, 125, 178, 205, 206, 214, 260, 262, 287, 306, 315, 335, 337, 340, 342, 352, 362), Kerala (225), Maharashtra (129, 178, 229, 257, 335, 337, 340, 342), Manipur (1), Meghalaya (1), Mizoram (1), Nagaland (1), Orissa (257, 335, 337, 340, 342), Punjab (29, 79, 342), Rajasthan (331, 333, 334, 335, 337, 338, 340, 342, 343, 344), Sikkim (1), Tamil Nadu (178, 205, 206, 286, 289, 293, 335, 337, 340, 342, 352), Tripura (1), Uttar Pradesh (2, 3, 326), Uttarakhand (1), West Bengal (1)
*Tapinoma wroughtonii* Forel, 1904	Haryana (408), Jammu & Kashmir (7, 114, 161, 162, 192, 315), Karnataka (352), West Bengal (352)
***Technomyrmex***	
*Technomyrmex albipes* (Smith, 1861)	Andaman and Nicobar Islands (254), Arunachal Pradesh (1), Assam (1), Gujarat (178), Haryana (408), Himachal Pradesh (214), Jammu & Kashmir (80), Karnataka (97, 178, 202, 203, 262, 264, 265, 287, 288, 291, 306, 362), Kerala (178, 225), Maharashtra (129, 178, 229), Manipur (244), Meghalaya (1), Nagaland (1), Orissa (415), Punjab (1), Sikkim (1), Tamil Nadu (97, 178), Uttar Pradesh (97), Uttarakhand (1), West Bengal (1)
*Technomyrmex bicolor* Emery, 1893	Karnataka (124), Kerala (225), Manipur (357)
*Technomyrmex brunneus* Forel, 1895	Delhi (1), Haryana (408), Karnataka (124, 262), Maharashtra (97, 178, 315), Sikkim (1)
*Technomyrmex elatior* Forel, 1902	Arunachal Pradesh (1), Assam (1), Himachal Pradesh (1), Kerala (225), Manipur (357), West Bengal (255)
*Technomyrmex horni* Forel, 1912	Haryana (97), Kerala (1)
*Technomyrmex indicus* Bolton, 2007 **(E)**	Karnataka (7, 97)
*Technomyrmex pratensis* (Smith, 1860)	Sikkim (1)
*Technomyrmex rector* Bolton, 2007 **(E)**	Arunachal Pradesh (1), Assam (1), Sikkim (1), Tamil Nadu (7, 97), Uttarakhand (1), West Bengal (1)
*Technomyrmex vitiensis* Mann, 1921	Himachal Pradesh (97), Karnataka (97, 127), Tamil Nadu (127)
**DORYLINAE**	
***Aenictus***	
*Aenictus aitkenii* Forel, 1901	Arunachal Pradesh (1), Assam (1), Himachal Pradesh (1), Jammu & Kashmir (80), Karnataka (7, 19, 20, 114, 184, 261, 306, 318, 383), Kerala (184, 318), Maharashtra (184, 318), Manipur (1), Sikkim (1), West Bengal (1)
*Aenictus ambiguus* Shuckard, 1840 **(E)**	Arunachal Pradesh (1), Assam (1), Gujarat (184, 355, 399), Himachal Pradesh (190, 192), Kerala (225), Maharashtra (184, 355, 399), Manipur (1), Nagaland (1), Sikkim (1), Uttar Pradesh (355, 399), West Bengal (1)
*Aenictus aratus* Forel, 1900	Himachal Pradesh (352, 399), Karnataka (287, 352, 362, 399), Kerala (352, 399), Maharashtra (352, 399), Tamil Nadu (352, 399), West Bengal (352)
*Aenictus arya* Forel, 1901 **(E)**	Karnataka (7, 114, 158, 184, 261, 352), West Bengal (352)
*Aenictus binghami* Forel, 1900	Andaman and Nicobar Islands (254), Arunachal Pradesh (1), Assam (1), Meghalaya (1), Tripura (1)
*Aenictus brevicornis* (Mayr, 1879)	Arunachal Pradesh (1), Assam (1), Gujarat (335, 340, 342), Haryana (333, 335, 340), Himachal Pradesh (342), Karnataka (184, 249, 261, 287, 333, 335, 340, 342, 352, 356, 399), Kerala (184, 249, 261, 333, 335, 340, 342, 352, 356, 399), Maharashtra (335, 342, 356), Meghalaya (1), Mizoram (1), Punjab (214, 342), Rajasthan (7, 333, 334, 335, 340, 342), Sikkim (1), Tamil Nadu (335, 340, 342), Uttar Pradesh (335, 340, 342, 352, 356, 399), West Bengal (1)
*Aenictus certus* Westwood, 1842	India (no further state, 32)
*Aenictus ceylonicus* (Mayr, 1866)	Arunachal Pradesh (1), Assam (1), Chhattisgarh (184), Himachal Pradesh (399), Jammu & Kashmir (1), Karnataka (184, 221, 261, 352, 362, 399), Madhya Pradesh (184), Maharashtra (7, 12, 114, 158, 184, 216, 229, 318, 352, 399), Manipur (1), Sikkim (1), Uttarakhand (1), Uttar Pradesh (399), West Bengal (1)
*Aenictus clavatus* Forel, 1901	Arunachal Pradesh (1), Assam (1), Gujarat (7, 184, 352, 355), Himachal Pradesh (1), Jammu & Kashmir (1), Karnataka (352, 355), Maharashtra (12, 184, 352, 355), Mizoram (1), Sikkim (1), West Bengal (1)
*Aenictus clavatus kanariensis* Forel, 1901 **(E)**	Karnataka (7, 114, 158, 184, 352), West Bengal (352)
*Aenictus clavitibia* Forel, 1901	Arunachal Pradesh (1), Sikkim (1), West Bengal (1)
*Aenictus dentatus* Forel, 1911	Maharashtra (399)
*Aenictus doryloides* Wilson, 1964 **(E)**	Arunachal Pradesh (1), Himachal Pradesh (216, 399), Jammu & Kashmir (80), Sikkim (1), Uttarakhand (1), West Bengal (1)
*Aenictus fergusoni* Forel, 1901	Andaman and Nicobar Islands (254, 352, 355), Arunachal Pradesh (1), Assam (1), Goa (7), Gujarat (158, 184, 261, 352, 355, 399), Karnataka (7, 12, 114, 158, 184, 261, 287, 399), Kerala (7, 12, 114, 158, 184, 261, 352, 355, 391, 399), Maharashtra (355), Meghalaya (1), Nagaland (1), Sikkim (1), Tamil Nadu (7, 213, 352, 399), West Bengal (1)
*Aenictus gleadowii* Forel, 1901 **(E)**	Andaman and Nicobar Islands (191), Karnataka (7, 114, 158, 184, 352), West Bengal (352)
*Aenictus hodgsoni* Forel, 1901	Andaman and Nicobar Islands (189)
*Aenictus indicus* Bharti, Wachkoo & Kumar, 2012 **(E)**	Tamil Nadu (7, 35)
*Aenictus laeviceps* (Smith, 1857)	Arunachal Pradesh (1), Assam (1), Kerala (1), Meghalaya (249)
*Aenictus latiscapus* Forel, 1901	Maharashtra (7, 114, 158, 184)
*Aenictus longi* Forel, 1901	Arunachal Pradesh (1), Assam (1), Meghalaya (1)
*Aenictus pachycerus* (Smith, 1858)	Arunachal Pradesh (1), Assam (1), Delhi (1), Himachal Pradesh (28, 184, 192, 261, 352, 399), Jammu & Kashmir (80), Karnataka (114, 184, 261, 287, 352), Kerala (184, 261, 352, 399), Maharashtra (352, 399), Manipur (1), Punjab (29, 79), Sikkim (1), Tamil Nadu (7, 184, 213, 261, 352), Uttar Pradesh (7, 261, 352), Uttarakhand (1), West Bengal (1)
*Aenictus peguensis* Emery, 1895	Himachal Pradesh (1), Uttarakhand (1)
*Aenictus piercei* Wheeler & Chapman, 1930	Himachal Pradesh (399)
*Aenictus porizonoides* Walker, 1860	Kerala (1)
*Aenictus pubescens* Smith, 1859 **(E)**	Arunachal Pradesh (1), Assam (1), Manipur (1), Sikkim (1), West Bengal (1)
*Aenictus punensis* Forel, 1901	Karnataka (287), Maharashtra (7, 114, 158, 184, 399)
*Aenictus sagei* Forel, 1901	Himachal Pradesh (7, 184, 192, 218, 399), Punjab (114, 158)
*Aenictus shillongensis* Mathew & Tiwari, 2000 **(E)**	Arunachal Pradesh (1), Meghalaya (1)
*Aenictus shuckardi* Forel, 1901	Arunachal Pradesh (1), Sikkim (1), West Bengal (1)
*Aenictus westwoodi* Forel, 1901	Kerala (225)
*Aenictus wilsoni* Bharti, Wachkoo & Kumar, 2012 **(E)**	Himachal Pradesh (7, 35)
*Aenictus wroughtonii* Forel, 1890	Kerala (184, 352, 399), Madhya Pradesh (352), Maharashtra (7, 12, 173, 184, 218, 352, 399), West Bengal (352)
***Cerapachys***	
*Cerapachys aitkenii* Forel, 1900	Goa (410), Haryana (21), Karnataka (7, 160, 180, 248, 249, 261, 319, 362), Kerala (60), Meghalaya (1), Punjab (21), West Bengal (319)
*Cerapachys alii* Bharti & Akbar, 2013 **(E)**	Kerala (60)
*Cerapachys anokha* Bharti & Akbar, 2013 **(E)**	Kerala (60)
*Cerapachys besucheti* Brown, 1975 **(E)**	Kerala (1), Tamil Nadu (7, 60, 108)
*Cerapachys biroi* Forel, 1907	Arunachal Pradesh (1), Assam (1), Goa (411), Himachal Pradesh (1), Jammu & Kashmir (80), Kerala (1), Manipur (1), Mizoram (1), Nagaland (1), Sikkim (1), Tripura (1), Uttarakhand (1), West Bengal (1)
*Cerapachys browni* Bharti & Wachkoo, 2013 **(E)**	Himachal Pradesh (42), Uttarakhand (1)
*Cerapachys costatus* Bharti & Wachkoo, 2013 **(E)**	Himachal Pradesh (42), Uttarakhand (1)
*Cerapachys indicus* Brown, 1975 **(E)**	Kerala (7, 60, 108)
*Cerapachys longitarsus* (Mayr, 1879)	Arunachal Pradesh (1), Assam (1), Himachal Pradesh (139, 180, 192), Jammu & Kashmir (80), Karnataka (180, 261, 335, 356, 362), Kerala (139, 180, 261, 335, 343, 352), Maharashtra (139, 139, 180, 261, 335, 343, 352, 356), Meghalaya (1), Nagaland (1), Orissa (139, 180), Punjab (79), Rajasthan (334, 335, 343), Sikkim (1), Tamil Nadu (139, 180, 249, 352, 356), Uttar Pradesh (352, 356), Uttarakhand (1), West Bengal (1)
*Cerapachys nayana* Bharti & Akbar, 2013 **(E)**	Karnataka (60), Kerala (60)
*Cerapachys parva* (Forel, 1900)	West Bengal (7)
*Cerapachys schoedli* Bharti & Akbar, 2013 **(E)**	Kerala (60)
*Cerapachys seema* Bharti & Akbar, 2013 **(E)**	Kerala (60)
*Cerapachys sulcinodis* Emery, 1889	Arunachal Pradesh (206), Meghalaya (206, 228, 248, 249, 335, 355), Sikkim (1), West Bengal (1)
*Cerapachys wighti* Bharti & Akbar, 2013 **(E)**	Kerala (60)
***Dorylus***	
*Dorylus fulvus* (Westwood, 1839)	West Bengal (7)
*Dorylus fulvus juvenculus* Shuckard, 1840	West Bengal (170)
*Dorylus labiatus* Shuckard, 1840	Arunachal Pradesh (1), Assam (1), Delhi (1), Gujarat (206, 237, 335, 337, 338, 340, 342, 344, 351, 355, 357), Haryana (206, 335, 337, 340, 342, 351, 355, 357), Himachal Pradesh (21, 184, 192, 206, 335, 337, 342, 355), Jammu & Kashmir (80), Karnataka (184, 261, 335, 337, 340, 342, 362), Maharashtra (184, 206, 335, 337, 342, 355), Manipur (1), Mizoram (1), Nagaland (1), Orissa (206, 335, 337, 342, 355, 357), Punjab (21, 29, 79, 335, 337, 340, 342), Rajasthan (116, 331, 334, 335, 337, 338, 339, 340, 342, 343, 344), Sikkim (1), Tamil Nadu (286), Uttar Pradesh (206, 335, 337, 342, 355, 357), Uttarakhand (1), West Bengal (1)
*Dorylus laevigatus* (Smith, 1857)	Arunachal Pradesh (206), Haryana (408)
*Dorylus orientalis* Westwood, 1835	Arunachal Pradesh (1), Assam (1), Bihar (298, 360), Delhi (1), Gujarat (335, 340, 342), Haryana (335, 340, 342, 351), Himachal Pradesh (184, 192, 298, 342), Jammu & Kashmir (80), Jharkhand (360), Karnataka (7, 184, 260, 261, 287, 298, 335, 340, 342), Kerala (225, 335, 340, 342, 352, 355), Maharashtra (184, 298, 335, 340, 342, 351, 352, 355, 399), Manipur (1), Meghalaya (1), Mizoram (1), Nagaland (1), Orissa (184, 298, 335, 340, 342, 351, 352, 355, 399), Punjab (79, 335, 340, 342), Rajasthan (116, 334, 335, 338, 339, 340, 342, 343, 344), Sikkim (1), Tamil Nadu (7, 167, 168, 184, 298, 335, 340, 342, 351, 399), Uttar Pradesh (298, 335, 340, 342, 355), Uttarakhand (1), West Bengal (1)
*Dorylus orientalis obscuriceps* Santschi, 1920	Arunachal Pradesh (1), Assam (1), Sikkim (1), Tamil Nadu (12, 114, 302, 303), West Bengal (1)
***Sphinctomyrmex***	
*Sphinctomyrmex furcatus* (Emery, 1893)	Kerala (7, 108), Tamil Nadu (7, 108)
*Sphinctomyrmex taylori* Forel, 1990	Orissa (108, 180), Sikkim (1), West Bengal (1)
**ECTATOMMINAE**	
***Gnamptogenys***	
*Gnamptogenys bicolor* (Emery, 1889)	Arunachal Pradesh (1), Assam (1), Kerala (1), Manipur (1), Meghalaya (180, 239, 248, 249), Mizoram (1), Sikkim (1), West Bengal (1)
*Gnamptogenys binghamii* (Forel, 1990)	Arunachal Pradesh (1), Assam (1), Kerala (7, 239), Manipur (1), Meghalaya (239), Mizoram (1), Sikkim (1), Tamil Nadu (239), West Bengal (1)
*Gnamptogenys coxalis* (Roger, 1860)	Andaman and Nicobar Islands (254), Karnataka (261)
*Gnamptogenys meghalaya* Lattke, 2004 **(E)**	Arunachal Pradesh (1), Meghalaya (1)
*Gnamptogenys menadensis* (Mayr, 1887)	Assam (356), West Bengal (356)
**FORMICINAE**	
***Acropyga***	
*Acropyga acutiventris* Roger, 1862	Andaman and Nicobar Islands (177, 189, 238, 254, 262), Arunachal Pradesh (1), Assam (1), Himachal Pradesh (23), Karnataka (262, 306, 362), Maharashtra (177), Manipur (1), Meghalaya (122), Mizoram (1), Nagaland (1), Orissa (238), Sikkim (1), Uttarakhand (1), West Bengal (1)
*Acropyga rubescens* Forel, 1894	Karnataka (7, 238)
***Anoplolepis***	
*Anoplolepis gracilipes* (Smith, 1857) **(I)**	Andaman and Nicobar Islands (117, 189, 254, 257, 262, 355, 378), Arunachal Pradesh (1), Assam (1), Goa (7, 410, 411, 412), Gujarat (1), Karnataka (7, 125, 214, 262, 264, 264, 265, 288, 327), Kerala (140, 225, 294, 329, 349, 352, 355, 357), Maharashtra (214, 229), Manipur (1), Meghalaya (1), Mizoram (1), Nagaland (1), Orissa (415), Punjab (255), Sikkim (355), Tamil Nadu (122, 219, 286), Tripura (247, 250), West Bengal (1)
***Camponotus***	
*Camponotus aethiops cachmiriensis* Emery, 1925 **(E)**	Jammu & Kashmir (7, 190, 192)
*Camponotus albosparsus* Bingham, 1903	Arunachal Pradesh (1), Assam (1), Himachal Pradesh (190, 192), Mizoram (1), Sikkim (1), West Bengal (1)
*Camponotus angusticollis* (Jerdon, 1851)	Assam (23, 249, 287, 331, 335, 340, 343, 351, 352, 356), Delhi (1), Goa (410, 411, 412), Gujarat (335, 338, 340, 344), Karnataka (7, 174, 262, 265, 287, 288, 327, 335, 340), Kerala (8, 225, 255, 301, 305, 335, 340, 352, 369), Maharashtra (174, 335, 340), Meghalaya (1), Orissa (335), Rajasthan (331, 334, 335, 338, 339, 340, 343, 344), Tamil Nadu (140, 219, 335, 340, 352), West Bengal (335, 351, 352, 356)
*Camponotus angusticollis sanguinolentus* Forel, 1895	Arunachal Pradesh (1), Assam (1), West Bengal (1)
*Camponotus arrogans* (Smith, 1858)	Arunachal Pradesh (1), Manipur (355, 357), Sikkim (1), West Bengal (1)
*Camponotus ashokai* Karmaly & Narenderan, 2006 **(E)**	Kerala (226)
*Camponotus auratus* Karavaiev, 1935	Andhra Pradesh (114)
*Camponotus badius* (Smith, 1857)	Andaman and Nicobar Islands (254), West Bengal (132)
*Camponotus barbatus* Roger, 1863	Kerala (305, 352), Orissa (415), West Bengal (352)
*Camponotus barbatus taylori* Forel, 1892	Kerala (140, 335, 337, 352, 355), Maharashtra (115, 174, 331, 335, 337, 352, 355), Orissa (174, 331, 335, 337, 352, 355), Rajasthan (331, 334, 335, 337, 338, 344), Sikkim (331, 335, 337, 352, 355), Tamil Nadu (174, 187, 335, 337, 352), West Bengal (214, 300, 335, 337, 352, 356)
*Camponotus binghamii* Forel, 1894	Kerala (305)
*Camponotus buddhae* Forel, 1892	Arunachal Pradesh (1), Himachal Pradesh (23, 174, 192, 243, 367), Jammu & Kashmir (7), Sikkim (1), West Bengal (1)
*Camponotus camelinus* (Smith, 1857)	Arunachal Pradesh (1), Meghalaya (1), Sikkim (1), Tripura (247, 250), West Bengal (1)
*Camponotus carin* Emery, 1889	Assam (187), Kerala (305), Maharashtra (174, 335, 337), Rajasthan (334, 335, 337)
*Camponotus cinerascens* (Fabricius, 1787)	Arunachal Pradesh (1), Assam (1), Manipur (1), Sikkim (1), West Bengal (1)
*Camponotus compressus* (Fabricius, 1787)	Andaman and Nicobar Islands (117, 254, 337, 340, 342, 355, 357), Arunachal Pradesh (1), Assam (1), Bihar (360), Delhi (1), Goa (410, 411, 412), Gujarat (227, 237, 335, 337, 338, 340, 342, 344), Haryana (258, 335, 337, 340, 351), Himachal Pradesh (174, 342), Jammu & Kashmir (67, 80), Jharkhand (360), Karnataka (7, 19, 20, 125, 256, 260, 262, 265, 287, 288, 306, 335, 337, 340, 342, 362), Kerala (8, 140, 225, 305, 369), Maharashtra (18, 154, 156, 174, 194, 229, 299, 335, 337, 340, 342), Manipur (1), Meghalaya (1), Mizoram (1), Nagaland (1), Orissa (205, 335, 337, 340, 342, 355, 357), Punjab (29, 79, 299, 335, 337, 340, 342), Rajasthan (116, 331, 333, 334, 335, 337, 338, 339, 340, 342, 343, 344), Sikkim (1), Tamil Nadu (112, 140, 205, 206, 219, 255, 256, 286, 289, 293, 299, 335, 337, 340, 342, 352, 355, 357, 383), Tripura (1), Uttar Pradesh (2, 3, 299, 326), Uttarakhand (1), West Bengal (1)
*Camponotus confucii* Forel, 1894	Arunachal Pradesh (206), Karnataka (177, 206, 262, 352), Kerala (1), Tamil Nadu (1), West Bengal (352)
*Camponotus cotesii* Forel, 1893	Arunachal Pradesh (1), Assam (1), Himachal Pradesh (175), Meghalaya (1), Uttarakhand (1)
*Camponotus crassisquamis* Forel, 1902	Arunachal Pradesh (1), Assam (1), Bihar (214), Jharkhand (360), Punjab (214)
*Camponotus dolendus* Forel, 1892	Andaman and Nicobar Islands (342, 355), Arunachal Pradesh (1), Maharashtra (214), Sikkim (1), Tamil Nadu (205, 342, 352, 355), West Bengal (1)
*Camponotus exiguoguttatus* Forel, 1886	Arunachal Pradesh (1), Assam (1)
*Camponotus festinus* (Smith, 1857)	Delhi (1), Haryana (351)
*Camponotus fulvopilosus* (De Geer, 1778)	Meghalaya (1)
*Camponotus gretae* Forel, 1902	Arunachal Pradesh (1), Assam (1), Sikkim (1), West Bengal (1)
*Camponotus himalayanus* Forel, 1893	Himachal Pradesh (1), Jammu & Kashmir (80)
*Camponotus holosericeus* Emery, 1889	Assam (222), Meghalaya (1)
*Camponotus horseshoetus* Datta & Ray Chaudhury, 1985 **(E)**	Himachal Pradesh (1), Nagaland (1)
*Camponotus indeflexus* (Walker, 1859)	Maharashtra (330)
*Camponotus invidus* Forel, 1892 **(E)**	Andaman and Nicobar Islands (117, 206, 254, 337, 341, 355), Arunachal Pradesh (206), Delhi (1), Haryana (335, 337, 341, 342, 351), Karnataka (265, 288), Kerala (117), Orissa (23, 114, 117, 174, 206, 335, 337, 341, 342, 351, 355, 356), Rajasthan (334, 335, 337, 342), Sikkim (206, 335, 337, 341, 342, 355), West Bengal (206, 335, 337, 341, 342, 351, 355, 356)
*Camponotus irritans* (Smith, 1857)	Andaman and Nicobar Islands (254), Arunachal Pradesh (206), Goa (411, 412), Gujarat (335, 338, 340, 344), Karnataka (262, 265, 287, 288, 335, 340), Kerala (301), Maharashtra (7), Orissa (415), Rajasthan (331, 334, 335, 338, 339, 340, 344), West Bengal (170, 206, 335, 356)
*Camponotus irritans carensis* Emery, 1920	Andaman and Nicobar Islands (7)
*Camponotus irritans pallidus* (Smith, 1857)	Andaman and Nicobar Islands (189), Karnataka (260)
*Camponotus kattensis* Bingham, 1903 **(E)**	Arunachal Pradesh (1), Assam (1), Himachal Pradesh (192), Sikkim (1), West Bengal (1)
*Camponotus keralensis* Karmaly & Narendran, 2006 **(E)**	Kerala (226)
*Camponotus lamarckii* Forel, 1892	Himachal Pradesh (23), Karnataka (175), Sikkim (355), Uttarakhand (1)
*Camponotus leonardi* Emery, 1889	Andaman and Nicobar Islands (189, 254)
*Camponotus longi* Forel, 1902 **(E)**	Arunachal Pradesh (1), Assam (1), Meghalaya (187, 188)
*Camponotus luteus* (Smith, 1858) **(E)**	Arunachal Pradesh (1), Assam (1), Sikkim (1), West Bengal (1)
*Camponotus mendax* Forel, 1895	Karnataka (163, 178, 194, 352), West Bengal (352)
*Camponotus misturus fornaronis* Forel, 1892	Kerala (8, 225, 369)
*Camponotus mitis* (Smith, 1858)	Andaman and Nicobar Islands (254, 355), Arunachal Pradesh (1), Assam (1), Bihar (214), Himachal Pradesh (255), Jammu & Kashmir (1), Jharkhand (214), Karnataka (214, 256, 256), Maharashtra (174, 335), Orissa (335), Punjab (1), Rajasthan (331, 334, 335, 338, 344), Sikkim (1), Tamil Nadu (256, 256, 335, 352, 355), Uttarakhand (1), West Bengal (1)
*Camponotus nicobarensis* Mayr, 1865	Andaman and Nicobar Islands (7, 117, 174, 189, 206, 254), Arunachal Pradesh (1), Assam (1), West Bengal (132, 193, 255)
*Camponotus nirvanae* Forel, 1893 **(E)**	Himachal Pradesh (1), Jammu & Kashmir (1), Karnataka (175, 352), Maharashtra (175), Tamil Nadu (352), Uttarakhand (1), West Bengal (352)
*Camponotus oblongus* (Smith, 1858)	Andaman and Nicobar Islands (117, 254, 355, 357), Assam (117, 355, 356, 357), Karnataka (125), Kerala (117), Manipur (355, 357), Sikkim (117, 355, 356, 357), West Bengal (355, 356, 357)
*Camponotus oblongus binominatus* Forel, 1916 **(E)**	Arunachal Pradesh (1), Himachal Pradesh (1), Jammu & Kashmir (80), Sikkim (1), Tamil Nadu (194), Uttarakhand (1), West Bengal (1)
*Camponotus opaciventris* Mayr, 1879	Arunachal Pradesh (1), Assam (1), Delhi (1), Himachal Pradesh (174), Jammu & Kashmir (1), Maharashtra (174), Orissa (174), Punjab (1), Sikkim (1), Uttarakhand (1), West Bengal (1)
*Camponotus parabarbatus* Bharti & Wachkoo, 2014 **(E)**	Himachal Pradesh (45), Uttarakhand (45)
*Camponotus parius* Emery, 1889	Andaman and Nicobar Islands (254), Arunachal Pradesh (1), Assam (1), Goa (410, 411, 412), Haryana (408), Himachal Pradesh (1), Jammu & Kashmir (80), Karnataka (7, 262, 265, 287, 288, 327, 362), Kerala (8, 140, 225, 305, 352, 369), Maharashtra (115), Meghalaya (1), Nagaland (1), Orissa (415), Punjab (79, 214), Sikkim (1), Tamil Nadu (293), Uttar Pradesh (2, 3, 326), Uttarakhand (1), West Bengal (1)
*Camponotus phragmaticola* Donisthorpe, 1943 **(E)**	Kerala (7, 114, 352), West Bengal (352)
*Camponotus puniceps* Donisthorpe, 1942 **(E)**	Kerala (1), Tamil Nadu (7, 114, 140, 352), West Bengal (352)
*Camponotus radiates* Forel, 1892 **(E)**	Goa (410, 411, 412), Karnataka (7, 174, 352, 357), Kerala (8, 225, 301, 369), Maharashtra (174), Manipur (357), Tripura (1), West Bengal (352)
*Camponotus reticulatus latitans* Forel, 1893	India (no further state, 32)
*Camponotus rothneyi* Forel, 1893	Orissa (175, 356), Sikkim (1), Uttarakhand (1), West Bengal (1)
*Camponotus rufifemur* Emery, 1900	Assam (7, 114)
*Camponotus rufoglaucus* (Jerdon, 1851)	Arunachal Pradesh (1), Assam (1), Delhi (1), Goa (1), Haryana (249, 262, 351, 352), Jammu & Kashmir (80), Karnataka (260, 262, 265, 288, 306, 352), Kerala (249, 255, 262, 305, 352), Maharashtra (129), Meghalaya (1), Nagaland (1), Punjab (79), Sikkim (1), Tamil Nadu (140, 219, 352), Tripura (1), West Bengal (1)
*Camponotus rufoglaucus tenuis* Forel, 1907 **(E)**	Tamil Nadu (194)
*Camponotus selene* (Emery, 1889)	Arunachal Pradesh (1), Assam (187, 188), Meghalaya (1), West Bengal (1)
*Camponotus selene obtusatus* (Emery, 1895)	Assam (7)
*Camponotus sericeus* (Fabricius, 1798)	Andhra Pradesh (128), Bihar (128, 214), Chhattisgarh (128), Goa (410, 411, 412), Gujarat (237, 335, 338, 340, 342, 344), Haryana (128, 333, 335, 340, 342, 357), Himachal Pradesh (342), Jammu & Kashmir (80), Jharkhand (128, 214), Karnataka (7, 125, 128, 174, 256, 260, 262, 265, 287, 288, 306, 333, 335, 340, 342, 352, 357, 362), Kerala (8, 225, 294, 349, 369), Madhya Pradesh (128), Maharashtra (174, 229, 335, 340, 342), Manipur (335, 342, 357), Meghalaya (249, 335, 340, 342, 357), Orissa (174, 335, 340, 342, 357), Punjab (79, 335, 340, 342), Rajasthan (333, 334, 335, 340, 342), Tamil Nadu (112, 119, 128, 140, 219, 256, 286, 289, 293, 335, 340, 342, 352, 357), West Bengal (174, 204, 255, 335, 340, 342, 352, 356, 357)
*Camponotus sericeus peguensis* Emery, 1895	Assam (114, 355), Sikkim (355)
*Camponotus siemsseni* Forel, 1901	Arunachal Pradesh (1), Himachal Pradesh (1), Sikkim (1), Uttarakhand (1), West Bengal (1)
*Camponotus singularis* (Smith, 1858)	Sikkim (355), West Bengal (174, 255)
*Camponotus sklarus* Bolton, 1995	Kerala (7, 114, 352), West Bengal (352)
*Camponotus socrates* Forel, 1904	Jammu & Kashmir (192)
*Camponotus strictus* (Jerdon, 1851)	Kerala (175, 249, 352), Meghalaya (1), West Bengal (352)
*Camponotus sylvaticus basalis* Smith, 1878 **(E)**	Gujarat (340), Himachal Pradesh (1), Jammu & Kashmir (163, 187, 188, 192, 340, 357), Manipur (357), Punjab (1), Uttarakhand (1)
*Camponotus sylvaticus paradichrous* Emery, 1925	Jammu & Kashmir (243)
*Camponotus thraso* Forel, 1893	Karnataka (214), Kerala (114, 352), West Bengal (352)
*Camponotus timidus* (Jerdon, 1851) **(E)**	Kerala (352), West Bengal (352)
*Camponotus varians* Roger, 1863	India (no further state, 32)
*Camponotus variegatus* (Smith, 1858)	Andaman and Nicobar Islands (254), Kerala (352), Maharashtra (214), Rajasthan (334, 335, 337), Tamil Nadu (140, 289, 335, 352, 352), West Bengal (352)
*Camponotus variegatus bacchus* (Smith, 1858)	Maharashtra (174), Tamil Nadu (256)
*Camponotus variegatus dulcis* Dalla Torre, 1893	Andaman and Nicobar Islands (189), Maharashtra (174)
*Camponotus variegatus fuscithorax* Dalla Torre, 1893	Maharashtra (174), Sikkim (1), West Bengal (1)
*Camponotus variegatus infuscus* Forel, 1892	Andaman and Nicobar Islands (254), Karnataka (287), Uttar Pradesh (3, 326)
*Camponotus variegatus somnificus* Forel, 1902	Kerala (352), Tamil Nadu (114, 187, 188, 352), West Bengal (352)
*Camponotus varius* Donisthorpe, 1943 **(E)**	Tamil Nadu (7, 352), West Bengal (352)
*Camponotus velox* (Jerdon, 1851) **(E)**	Karnataka (352), Kerala (352), West Bengal (352)
*Camponotus vitreus* (Smith, 1860)	Andaman and Nicobar Islands (189, 254)
*Camponotus vitreus angustulus* Emery, 1925	Assam (356), West Bengal (114, 175, 356)
*Camponotus wasmanni* Emery, 1893	Assam (7, 23, 178, 355), Maharashtra (115), Meghalaya (1), Sikkim (1), Uttarakhand (416), West Bengal (1)
*Camponotus wasmanni mutilarius* Emery, 1893	Arunachal Pradesh (1), Himachal Pradesh (1, 178), Jammu & Kashmir (1), Punjab (23), Sikkim (156), Uttarakhand (1)
*Camponotus wroughtonii* Forel, 1893	Arunachal Pradesh (1), Sikkim (1), West Bengal (1)
***Cataglyphis***	
*Cataglyphis cugiai* Menozzi, 1939	Jammu & Kashmir (4, 80, 243)
*Cataglyphis indica* Pisarski, 1962 **(E)**	Maharashtra (4, 271)
*Cataglyphis longipedem* (Eichwald, 1841)	Bihar (255), Haryana (351), Madhya Pradesh (177), Meghalaya (249), Punjab (249, 255, 351, 352, 356), Rajasthan (7, 177), Tamil Nadu (352), Uttar Pradesh (177, 255)
*Cataglyphis setipes* (Forel, 1894)	Arunachal Pradesh (206), Delhi (1), Gujarat (338, 340, 344), Haryana (340), Himachal Pradesh (370), Jammu & Kashmir (370), Madhya Pradesh (1), Meghalaya (206, 355), Punjab (1,79, 116, 206, 214, 331, 339, 340, 355), Rajasthan (4, 116, 331, 334, 338, 339, 340, 344, 386), Sikkim (206, 355), Tamil Nadu (206, 355), Uttarakhand (370), Uttar Pradesh (116), West Bengal (206, 340, 355)
***Echinopla***	
*Echinopla cherapunjiensis* Bharti & Gul, 2012	Arunachal Pradesh (1), Meghalaya (1)
*Echinopla lineata senilis* Mayr, 1862	Andaman and Nicobar Islands (163, 189, 254, 406)
***Formica***	
*Formica candida* Smith, 1878	Himachal Pradesh (1), Jammu & Kashmir (7, 313), Uttarakhand (1)
*Formica clara* Forel, 1886	Jammu & Kashmir (7, 80, 311)
*Formica cunicularia* Latreille, 1798	Himachal Pradesh (243), Jammu & Kashmir (80, 190, 243), Uttarakhand (1)
*Formica fusca* Linnaeus, 1758	Himachal Pradesh (177, 341), Jammu & Kashmir (67, 80, 341), Madhya Pradesh (177, 341, 355), Sikkim (192, 341, 355), Uttarakhand (1)
*Formica gagates* Latreille, 1798	Himachal Pradesh (177, 192, 243), Jammu & Kashmir (80)
*Formica gagatoides* Ruzsky, 1904	Himachal Pradesh (1), Jammu & Kashmir (80)
*Formica kashmirica* Starcke, 1935 **(E)**	Jammu & Kashmir (312)
*Formica picea* Nylander, 1846	Himachal Pradesh (243), Jammu & Kashmir (243)
*Formica polyctena* Foerster, 1850	Himachal Pradesh (1), Jammu & Kashmir (1)
*Formica rufibarbis* Fabricius, 1793	Himachal Pradesh (177, 177, 192, 192, 243, 341, 355), Jammu & Kashmir (190)
*Formica sanguinea* Latreille, 1798	Himachal Pradesh (23, 177, 192, 243, 341, 381), Jammu & Kashmir (67, 80)
*Formica truncorum* Fabricius, 1804	Himachal Pradesh (177, 192, 243, 381), Jammu & Kashmir (67, 80)
***Gesomyrmex***	
*Gesomyrmex spatulatus* Cole, 1949 **(E)**	Assam (1)
***Lasius***	
*Lasius alienoflavus* Bingham, 1903	Himachal Pradesh (51), Jammu & Kashmir (7, 51, 80, 121), Uttarakhand (1)
*Lasius alienus* (Foerster, 1850)	Himachal Pradesh (1), Jammu & Kashmir (67, 80, 121, 190, 394), Uttarakhand (1)
*Lasius bicornis* (Foerster, 1850)	Jammu & Kashmir (7, 121, 133, 394)
*Lasius breviscapus* Seifert, 1992 **(E)**	Himachal Pradesh (309)
*Lasius brunneus* (Latreille, 1798)	Himachal Pradesh (1), Jammu & Kashmir (80)
*Lasius crinitus* (Smith, 1858)	Jammu & Kashmir (177, 192), Sikkim (121), West Bengal (7, 121, 132, 394)
*Lasius draco* Collingwood, 1982	Arunachal Pradesh (1), Sikkim (1), West Bengal (1)
*Lasius elevates* Bharti & Gul, 2013 **(E)**	Himachal Pradesh (7, 50)
*Lasius himalayanus* Bingham, 1903	Himachal Pradesh (23, 309), Jammu & Kashmir (309), Uttarakhand (1)
*Lasius lawarai* Seifert, 1992	Arunachal Pradesh (1), Sikkim (1), West Bengal (1)
*Lasius magnus* Seifert, 1992	Arunachal Pradesh (1), Meghalaya (309), Sikkim (1), West Bengal (1)
*Lasius mikir* Collingwood, 1982 **(E)**	Assam (121), Sikkim (1), West Bengal (1)
*Lasius niger* (Linnaeus, 1758)	Himachal Pradesh (1), Jammu & Kashmir (80, 121)
*Lasius wittmeri* Seifert, 1992	Jammu & Kashmir (309)
***Lepisiota***	
*Lepisiota annandalei* (Mukerjee, 1930) **(E)**	Himachal Pradesh (255), Punjab (1), Sikkim (1), West Bengal (1)
*Lepisiota bipartita* (Smith, 1861)	Andhra Pradesh (340), Gujarat (340), Haryana (340), Himachal Pradesh (177), Jammu & Kashmir (1), Karnataka (340), Maharashtra (340), Meghalaya (340), Punjab (340), Rajasthan (177, 339, 340), Uttarakhand (1), West Bengal (170, 177, 300, 340)
*Lepisiota capensis* (Mayr, 1862)	Arunachal Pradesh (1), Assam (1), Bihar (214), Goa (410, 411, 412), Haryana (408), Himachal Pradesh (177), Jammu & Kashmir (67, 80), Jharkhand (214), Karnataka (287), Madhya Pradesh (177), Maharashtra (177, 249), Manipur (1), Meghalaya (1), Mizoram (1), Sikkim (1), Uttarakhand (1), West Bengal (1)
*Lepisiota capensis lunaris* (Emery, 1893)	Himachal Pradesh (214), Jammu & Kashmir (190)
*Lepisiota capensis simplex* (Forel, 1892)	Haryana (408), Himachal Pradesh (1), Jammu & Kashmir (1), Meghalaya (1), Orissa (177, 249, 356), Punjab (1), Uttarakhand (1), West Bengal (356)
*Lepisiota fergusoni* (Forel, 1895)	Karnataka (287), Kerala (114, 178, 352), West Bengal (352)
*Lepisiota frauenfeldi* (Mayr, 1855)	Andhra Pradesh (335, 342, 352, 357), Delhi (1), Gujarat (338, 344), Haryana (335), Himachal Pradesh (255, 342), Karnataka (262, 335, 342, 362), Maharashtra (229, 335, 342), Manipur (357), Meghalaya (249, 335, 342), Punjab (335, 342), Rajasthan (116, 331, 334, 335, 338, 342, 344), West Bengal (116, 170, 171, 177, 249, 255, 300, 335, 342, 352, 356, 357)
*Lepisiota frauenfeldi integra* (Forel, 1894)	Himachal Pradesh (177, 192), Jammu & Kashmir (80), Madhya Pradesh (177), Meghalaya (1), Punjab (79), Uttarakhand (1)
*Lepisiota modesta* (Forel, 1894)	Himachal Pradesh (25, 177), Punjab (1), Uttarakhand (1)
*Lepisiota opaca* (Forel, 1892)	Arunachal Pradesh (1), Assam (1), Himachal Pradesh (1), Goa (177, 262, 287, 352, 410, 411, 412), Jammu & Kashmir (80), Karnataka (7, 177, 178, 262, 265, 287, 288, 306, 352), Kerala (225), Maharashtra (229), Sikkim (1), West Bengal (1)
*Lepisiota opaca pulchella* (Forel, 1892)	Arunachal Pradesh (1), Assam (1), Himachal Pradesh (1), Jammu & Kashmir (80), Maharashtra (177), Punjab (79), Sikkim (1), Uttarakhand (1), West Bengal (1)
*Lepisiota rothneyi* (Forel, 1894)	Karnataka (177), Kerala (329), Orissa (177), Tamil Nadu (7), Uttarakhand (1), West Bengal (114, 177, 300, 356, 391)
*Lepisiota rothneyi watsonii* (Forel, 1894)	West Bengal (384)
*Lepisiota rothneyi wroughtonii* (Forel, 1902)	Himachal Pradesh (1), Kerala (352), Tamil Nadu (114, 187, 188, 352, 391), Uttarakhand (1), West Bengal (352)
*Lepisiota sericea* (Forel, 1892)	Himachal Pradesh (177), Jammu & Kashmir (1), Maharashtra (177), Uttar Pradesh (177), Uttarakhand (1)
***Myrmoteras***	
*Myrmoteras agostii* Bharti & Akbar, 2014 **(E)**	Kerala (63)
*Myrmoteras brachygnathum* Moffett, 1985 **(E)**	Kerala (63), Tamil Nadu (7, 253)
*Myrmoteras indicum* Moffett, 1985 **(E)**	Karnataka (287), Kerala (253, 287), Tamil Nadu (7, 253, 287)
*Myrmoteras moffetti* Bharti & Akbar, 2014 **(E)**	Kerala (63)
*Myrmoteras scabrum* Moffett, 1985 **(E)**	Kerala (253)
***Nylanderia***	
*Nylanderia assimilis* (Jerdon, 1851) **(E)**	Kerala (352), West Bengal (352)
*Nylanderia birmana* (Forel, 1902)	Himachal Pradesh (1), Uttarakhand (1)
*Nylanderia bourbonica* (Forel, 1886)	Andaman and Nicobar Islands (189, 254), Arunachal Pradesh (1), Jammu & Kashmir (80), Manipur (357), Sikkim (1), Tamil Nadu (206, 352, 357), West Bengal (1)
*Nylanderia himalayana* Wachkoo & Bharti, 2015 **(E)**	Himachal Pradesh (1)
*Nylanderia indica* (Forel, 1894)	Andaman and Nicobar Islands (117, 189, 254), Andhra Pradesh (187, 188), Arunachal Pradesh (206), Himachal Pradesh (214, 341), Jammu & Kashmir (7), Karnataka (177, 262), Maharashtra (177, 206, 341, 391), Sikkim (1), Tamil Nadu (187, 188), Uttarakhand (1), West Bengal (1)
*Nylanderia smythiesii* (Forel, 1894) **(E)**	Himachal Pradesh (177, 192, 371), Jammu & Kashmir (371), Punjab (1), Uttarakhand (1)
*Nylanderia taylori* (Forel, 1894)	Assam (1), Himachal Pradesh (1), Jammu & Kashmir (80), Kerala (1), Maharashtra (187, 188), Orissa (114, 177, 335, 356, 391), Rajasthan (334, 335), Sikkim (1), Uttarakhand (1), West Bengal (1)
*Nylanderia vividula* (Nylander, 1846) **(I)**	West Bengal (171)
*Nylanderia yerburyi* (Forel, 1894)	Andaman and Nicobar Islands (254), Himachal Pradesh (1), Karnataka (362), Kerala (352), Tamil Nadu (177, 352), West Bengal (300, 352)
***Oecophylla***	
*Oecophylla smaragdina* (Fabricius, 1775)	Andaman and Nicobar Islands (117, 172, 189, 206, 254, 257, 340, 342, 355, 357), Andhra Pradesh (9), Arunachal Pradesh (1), Assam (1), Bihar (122, 214, 257), Delhi (1), Goa (7, 410, 411, 412), Gujarat (237, 335, 340, 342), Haryana (335, 340), Himachal Pradesh (177, 257, 335, 340, 342), Jammu & Kashmir (80), Jharkhand (122, 214, 257), Karnataka (7, 19, 20, 124, 125, 206, 256, 260, 262, 264, 264, 265, 287, 288, 291, 306, 327, 335, 340, 342, 352, 355, 357, 362), Kerala (206, 225, 294, 305, 329, 335, 340, 342, 352, 355, 357), Madhya Pradesh (269), Maharashtra (115, 129, 177, 214, 229, 335, 340, 342), Manipur (1), Meghalaya (1), Mizoram (248), Nagaland (1), Orissa (206, 257, 335, 340, 342, 355), Punjab (52, 79, 342), Rajasthan (332, 334, 335, 340, 342), Sikkim (1), Tamil Nadu (122, 140, 206, 219, 256, 286, 289, 293, 335, 340, 342, 352, 355, 357), Tripura (1), Uttar Pradesh (122, 292, 335, 340, 342), Uttarakhand (1), West Bengal (1)
***Paraparatrechina***	
*Paraparatrechina aseta* (Forel, 1902)	Gujarat (338, 340, 344), Himachal Pradesh (46), Jammu & Kashmir (80), Sikkim (1), West Bengal (1)
***Paratrechina***	
*Paratrechina longicornis* (Latreille, 1802) **(I)**	Andaman and Nicobar Islands (189, 254, 257, 357), Arunachal Pradesh (1), Assam (1), Delhi (1), Goa (410), Gujarat (237), Himachal Pradesh (177, 342), Jammu & Kashmir (80), Karnataka (125, 205, 260, 262, 264, 264, 265, 287, 288, 306, 327, 357, 362), Kerala (294, 305), Maharashtra (115, 129, 177, 214, 229, 257, 335, 342), Manipur (1), Meghalaya (1), Mizoram (1), Nagaland (1), Orissa (205, 257, 335, 342, 357), Punjab (29, 79, 214), Rajasthan (334, 335, 342), Sikkim (1), Tamil Nadu (122, 205, 219, 289, 335, 342, 352, 357), Tripura (1), Uttar Pradesh (122, 214, 257, 335, 342), Uttarakhand (1), West Bengal (1)
***Plagiolepis***	
*Plagiolepis alluaudi* Emery, 1894 **(I)**	India (240, 380)
*Plagiolepis balestrierii* Menozzi, 1939	Arunachal Pradesh (1), Assam (1), Jammu & Kashmir (243), Sikkim (1), West Bengal (1)
*Plagiolepis dichroa* Forel, 1902	Himachal Pradesh (1), Jammu & Kashmir (80), Karnataka (362), Meghalaya (1), Sikkim (1), West Bengal (1)
*Plagiolepis exigua* Forel, 1894	Karnataka (177, 287, 362), Maharashtra (7, 177, 314, 386, 391)
*Plagiolepis jerdonii* Forel, 1894	Gujarat (335, 340), Himachal Pradesh (1), Jammu & Kashmir (80), Karnataka (125, 205, 262, 332, 333, 335, 340, 362), Kerala (114, 205, 262, 290, 332, 333, 335, 340, 352), Maharashtra (177, 205, 332, 333, 335, 340, 352, 391), Punjab (1), Rajasthan (332, 333, 334, 335, 340), Uttarakhand (1), West Bengal (205, 335, 352)
*Plagiolepis moelleri* Bingham, 1903 **(E)**	Sikkim (1)
*Plagiolepis pontii* Menozzi, 1939	Arunachal Pradesh (1), Sikkim (1), West Bengal (1)
*Plagiolepis rogeri* Forel, 1894	Karnataka (177, 352), West Bengal (352)
***Polyrhachis***	
*Polyrhachis abdominalis* Smith, 1858	Andaman and Nicobar Islands (297), Meghalaya (1)
*Polyrhachis aculeate* Mayr, 1879	Karnataka (176, 352), Kerala (176, 232, 297, 297, 352), West Bengal (352)
*Polyrhachis aedipus* Forel, 1893	Andaman and Nicobar Islands (7)
*Polyrhachis alatisquamis* Forel, 1893	Andaman and Nicobar Islands (232)
*Polyrhachis armata* (Le Guillou, 1842)	Andaman and Nicobar Islands (117, 254, 297, 297), Arunachal Pradesh (206, 319), Assam (1), Meghalaya (1), West Bengal (255, 319)
*Polyrhachis armata defensa* Smith, 1857	Assam (172, 176)
*Polyrhachis bicolor* Smith, 1858	Andaman and Nicobar Islands (231, 254, 257, 297), Arunachal Pradesh (206), Meghalaya (1), Sikkim (1), West Bengal (1)
*Polyrhachis bicolor aurinasis* Forel, 1901	West Bengal (183)
*Polyrhachis bihamata* (Drury, 1773)	Andaman and Nicobar Islands (189, 212, 254), Assam (297), Karnataka (362)
*Polyrhachis binghamii* Forel, 1893	Kerala (7, 140, 352), West Bengal (352)
*Polyrhachis calypso* Forel, 1911	Andaman and Nicobar Islands (85)
*Polyrhachis convexa* Roger, 1863	Arunachal Pradesh (206, 358), Kerala (225), Meghalaya (249)
*Polyrhachis corporaali* Santschi, 1928	Karnataka (352), West Bengal (352)
*Polyrhachis dives* Smith, 1857	Andaman and Nicobar Islands (254), Arunachal Pradesh (1), Assam (172, 231, 297, 382), Karnataka (262), Meghalaya (1), Sikkim (196, 206, 352, 355), Tamil Nadu (206, 352, 355), Tripura (1), West Bengal (132, 204)
*Polyrhachis dives belli* Forel, 1912	Karnataka (198, 352), Meghalaya (228), West Bengal (352)
*Polyrhachis exercita* (Walker, 1859)	Goa (411, 412), Gujarat (340), Jammu & Kashmir (80), Karnataka (7, 176, 202, 256, 262, 287, 297, 327), Kerala (176, 262, 301, 305, 340, 352, 356), Maharashtra (176), Orissa (176), Tamil Nadu (7, 140, 213, 256, 262, 297, 297, 352), Tripura (1), West Bengal (176, 262, 340, 352, 356)
*Polyrhachis exercita lucidiventris* Forel, 1907 **(E)**	Himachal Pradesh (1), Karnataka (194), Kerala (1), Orissa (194)
*Polyrhachis exercita obtusisquama* Forel, 1902 **(E)**	Himachal Pradesh (1), Karnataka (352), Maharashtra (187, 188), West Bengal (352)
*Polyrhachis exercita rastrata* Emery, 1889	Assam (255)
*Polyrhachis furcata* Smith, 1858	Assam (114, 172, 176, 248, 249), Meghalaya (248, 249), West Bengal (1)
*Polyrhachis gracilior* Forel, 1893 **(E)**	Arunachal Pradesh (1), Assam (1), Karnataka (202, 262), Kerala (7, 84, 114, 176, 262, 297, 352), West Bengal (352)
*Polyrhachis halidayi* Emery, 1889	Arunachal Pradesh (382)
*Polyrhachis hauxwelli* Bingham, 1903	Karnataka (265, 327)
*Polyrhachis hector* Smith, 1857	Andaman and Nicobar Islands (254), Meghalaya (248)
*Polyrhachis hemiopticoides* Mukerjee, 1930	Karnataka (256), Sikkim (1), Tamil Nadu (232, 256), West Bengal (1)
*Polyrhachis hippomanes* Smith, 1861	Arunachal Pradesh (358)
*Polyrhachis hippomanes ceylonensis* Emery, 1893	Arunachal Pradesh (206, 358), Meghalaya (1), Tripura (247, 250)
*Polyrhachis horni* Emery, 1901	Bihar (262), Karnataka (262)
*Polyrhachis illaudata* Walker, 1859	Andaman and Nicobar Islands (117, 206, 254, 355), Arunachal Pradesh (1), Assam (1), Goa (411, 412), Himachal Pradesh (1), Jammu & Kashmir (80), Karnataka (117, 176, 206, 248, 249, 250, 255, 262, 288, 319, 352, 355), Kerala (7, 84, 114, 117, 140, 176, 206, 225, 248, 249, 250, 255, 262, 297, 319, 329, 352, 355), Meghalaya (1), Mizoram (1), Sikkim (1), Tamil Nadu (140, 206, 219, 262, 352, 355), Tripura (247, 250), Uttarakhand (1), West Bengal (1)
*Polyrhachis illaudata intermedia* Forel, 1886	Assam (114, 172, 176, 249, 297), Meghalaya (249)
*Polyrhachis illaudata pauperata* Emery, 1889	West Bengal (194)
*Polyrhachis indificans* (Jerdon, 1851) **(E)**	Kerala (352), West Bengal (352)
*Polyrhachis lacteipennis* Smith, 1858	Arunachal Pradesh (1), Assam (1), Delhi (1), Gujarat (237, 335, 337, 338, 340, 344), Haryana (335, 337, 340), Himachal Pradesh (176), Jammu & Kashmir (67, 80, 84), Karnataka (256, 265, 287, 288), Maharashtra (176, 194, 229), Manipur (1), Punjab (79, 214), Rajasthan (7, 335, 337, 340, 343), Sikkim (1), Tamil Nadu (256, 335, 337, 340, 352, 357), Uttarakhand (1), West Bengal (1)
*Polyrhachis lacteipennis obsoleta* Forel, 1893 **(E)**	Maharashtra (176)
*Polyrhachis laevissima* Smith, 1858	Andaman and Nicobar Islands (117, 189, 254, 257), Arunachal Pradesh (1), Assam (1), Maharashtra (117, 257), Meghalaya (1), Orissa (117, 176, 248, 249, 257), Sikkim (1), West Bengal (1)
*Polyrhachis laevissima dichroa* Forel, 1893	Assam (114, 172, 176)
*Polyrhachis menelas* Forel, 1904	Himachal Pradesh (190, 192), Jammu & Kashmir (1), Punjab (1), Sikkim (1), Uttarakhand (1)
*Polyrhachis moeschi* Forel, 1912	Andaman and Nicobar Islands (297)
*Polyrhachis mutata* Smith, 1858	Meghalaya (1)
*Polyrhachis narendrani* Karmaly, 2004 **(E)**	Kerala (223)
*Polyrhachis numeria* Smith, 1861	Andaman and Nicobar Islands (7)
*Polyrhachis pagana* Santschi, 1928	Karnataka (304), Tamil Nadu (289)
*Polyrhachis proxima* Roger, 1863	Assam (199), Kerala (305), Meghalaya (1)
*Polyrhachis pubescens* Mayr, 1879	Kerala (305)
*Polyrhachis punctillata* Roger, 1863	Gujarat (340), Karnataka (249, 256, 287, 340, 352, 357, 362), Kerala (225, 297, 352), Manipur (357), Meghalaya (1), Tamil Nadu (7, 256, 352, 357), West Bengal (352)
*Polyrhachis punctillata fergusoni* Forel, 1902 **(E)**	Himachal Pradesh (1), Kerala (114, 187, 188, 352), West Bengal (352)
*Polyrhachis punctillata smythiesii* Forel, 1895	Himachal Pradesh (178, 192), Jammu & Kashmir (80), Kerala (297), Uttarakhand (1)
*Polyrhachis punjabi* Bharti, 2003 **(E)**	Himachal Pradesh (27), Punjab (27)
*Polyrhachis rastellata* (Latreille, 1802)	Andaman and Nicobar Islands (254, introduced but no evidence for establishment), Arunachal Pradesh (1), Assam (1), Karnataka (176, 248, 249, 256, 265, 288, 306, 319, 352, 362), Kerala (305, 319), Maharashtra (115, 153, 248, 249), Manipur (1), Meghalaya (1), Mizoram (1), Nagaland (1), Orissa (415), Tamil Nadu (219, 256), West Bengal (319, 352)
*Polyrhachis rupicapra* Roger, 1863	Karnataka (256, 287), Tamil Nadu (256)
*Polyrhachis saevissima* Smith, 1860	Assam (172), West Bengal (300)
*Polyrhachis saevissima argentea* Mayr, 1862	Karnataka (176), West Bengal (300)
*Polyrhachis scissa* (Roger, 1862)	Karnataka (7), Kerala (7, 140, 141, 297, 352), Tamil Nadu (140, 141, 297, 352), West Bengal (352)
*Polyrhachis sculpturata* Smith, 1860	Assam (176)
*Polyrhachis spinigera* Mayr, 1879	Assam (172), West Bengal (130, 299, 300)
*Polyrhachis striata* Mayr, 1862	Arunachal Pradesh (1), Assam (1), Meghalaya (1), Sikkim (1)
*Polyrhachis striatorugosa* Mayr, 1862	Arunachal Pradesh (382)
*Polyrhachis subpilosa* Emery, 1895	Manipur (7)
*Polyrhachis sylvicola* (Jerdon, 1851)	Kerala (352), West Bengal (352)
*Polyrhachis textor brunneogaster* Donisthorpe, 1937	Andaman and Nicobar Islands (7, 137, 297)
*Polyrhachis thompsoni* Bingham, 1903	Himachal Pradesh (297), Sikkim (355), Tripura (250)
*Polyrhachis thrinax* Roger, 1863	Andaman and Nicobar Islands (254), Arunachal Pradesh (1), Assam (104, 297), Karnataka (176, 262, 319, 352, 356), Kerala (7, 104, 140, 140, 176, 262, 294, 297, 319, 352), Maharashtra (104, 297), Sikkim (1), Tamil Nadu (319), West Bengal (1)
*Polyrhachis thrinax lancearia* Forel, 1893	Karnataka (176, 297), Kerala (176, 297)
*Polyrhachis tibialis* Smith, 1858	Andaman and Nicobar Islands (254), Arunachal Pradesh (1), Assam (382), Karnataka (7, 124, 206, 248, 249, 262, 265, 288, 319, 352, 356), Kerala (140, 206, 262, 297, 319, 352, 352), Meghalaya (1), Sikkim (1), Tamil Nadu (219), Uttarakhand (1), West Bengal (1)
*Polyrhachis tibialis caligata* Emery, 1895	Assam (297), Maharashtra (129), Uttarakhand (1)
*Polyrhachis tibialis parsis* Emery, 1900	Karnataka (7), Kerala (140, 352), West Bengal (352)
*Polyrhachis tubericeps* Forel, 1893	Himachal Pradesh (319), Kerala (319), Sikkim (1), Uttar Pradesh (176, 319), West Bengal (1)
*Polyrhachis tyrannical* Smith, 1858	Tamil Nadu (297)
*Polyrhachis vicina* Roger, 1863	Arunachal Pradesh (358), Meghalaya (249)
*Polyrhachis wallacei* Emery, 1887	Andaman and Nicobar Islands (7, 297)
*Polyrhachis wroughtonii* Forel, 1894	Karnataka (7, 177, 178, 233, 352), Kerala (233, 297), Maharashtra (233, 297), West Bengal (352)
***Prenolepis***	
*Prenolepis fisheri* Bharti & Wachkoo, 2012 **(E)**	Uttarakhand (1, 7, 40)
*Prenolepis melanogaster* Emery, 1893	Manipur (357)
*Prenolepis naoroji* Forel, 1902	Arunachal Pradesh (1), Assam (1), Haryana (408), Himachal Pradesh (7, 23, 40), Jammu & Kashmir (40, 80), Punjab (1), Uttarakhand (1)
***Pseudolasius***	
*Pseudolasius binghami* Emery, 1911	Sikkim (159)
*Pseudolasius diversus* Wachkoo & Bharti, 2014 **(E)**	Uttarakhand (1, 372)
*Pseudolasius emeryi* Forel, 1911	Sikkim (7)
*Pseudolasius familiaris* (Smith, 1860)	Arunachal Pradesh (206), Himachal Pradesh (1), Jammu & Kashmir (80), Meghalaya (1), Sikkim (192, 249, 355)
*Pseudolasius machhediensis* Bharti, Gul & Sharma, 2012 **(E)**	Himachal Pradesh (73), Jammu & Kashmir (73)
*Pseudolasius polymorphicus* Wachkoo & Bharti, 2014 **(E)**	Himachal Pradesh (372)
**LEPTANILLINAE**	
***Leptanilla***	
*Leptanilla escheri* (Kutter, 1948) **(E)**	Kerala (12, 13), Tamil Nadu (7, 12, 13, 54)
*Leptanilla lamellate* Bharti & Kumar, 2012 **(E)**	Himachal Pradesh (7, 54)
***Protanilla***	
*Protanilla wardi* Bharti & Akbar, 2015 **(E)**	Kerala (1)
***Yavnella***	
*Yavnella indica* Kugler, 1987 **(E)**	Kerala (7, 236)
**MYRMICINAE**	
***Anillomyrma***	
*Anillomyrma decamera* (Emery, 1901)	Bihar (92, 149)
***Aphaenogaster***	
*Aphaenogaster annandalei* Mukerjee, 1930 **(E)**	Himachal Pradesh (255)
*Aphaenogaster beccarii* Emery, 1887	Andaman and Nicobar Islands (254, 257), Arunachal Pradesh (1), Goa (410, 411, 412), Karnataka (7, 19, 20, 188, 262, 265, 287, 288, 306, 319, 352, 362), Maharashtra (152, 188, 214, 262, 287, 319, 352), Sikkim (1), Tamil Nadu (219), Tripura (1), West Bengal (1)
*Aphaenogaster beesoni* Donisthorpe, 1933	Himachal Pradesh (7, 76, 134), Jammu & Kashmir (1), Uttarakhand (1)
*Aphaenogaster cavernicola* Donisthorpe, 1938 **(E)**	Himachal Pradesh (7, 138)
*Aphaenogaster cristata* (Forel, 1902) **(E)**	Arunachal Pradesh (1), Himachal Pradesh (7, 186, 188, 192, 355), Jammu & Kashmir (80), Sikkim (1), West Bengal (1)
*Aphaenogaster feae* Emery, 1889	Andaman and Nicobar Islands (254), Arunachal Pradesh (1), Assam (188), Goa (411, 412), Jammu & Kashmir (80), Sikkim (1), West Bengal (1)
*Aphaenogaster feae nicobarensis* (Forel, 1903) **(E)**	Andaman and Nicobar Islands (189, 254)
*Aphaenogaster longiceps* (Smith, 1858)	Arunachal Pradesh (1), Sikkim (1), West Bengal (1)
*Aphaenogaster rothneyi* (Forel, 1902)	Arunachal Pradesh (1), Himachal Pradesh (186, 188), Jammu & Kashmir (80), Madhya Pradesh (186, 188, 352, 355), Meghalaya (1), Sikkim (1), Uttarakhand (1), West Bengal (1)
*Aphaenogaster sagei* (Forel, 1902)	Himachal Pradesh (21, 186, 188, 190, 192, 243, 341), Jammu & Kashmir (80), Meghalaya (1), Uttarakhand (1)
*Aphaenogaster sagei pachei* (Forel, 1906)	Himachal Pradesh (1), Jammu & Kashmir (80), Uttarakhand (1)
*Aphaenogaster schurri* (Forel, 1902)	Arunachal Pradesh (1), Himachal Pradesh (1), Madhya Pradesh (186, 188), Maharashtra (115), Meghalaya (1), Sikkim (1), West Bengal (1)
*Aphaenogaster singaporensis* (Smith, 1858)	Andaman and Nicobar Islands (254)
*Aphaenogaster smythiesii* (Forel, 1902)	Arunachal Pradesh (1), Himachal Pradesh (186, 188, 214), Jammu & Kashmir (67, 80, 190), Meghalaya (206, 249, 355), Sikkim (1), Uttarakhand (1), West Bengal (1)
*Aphaenogaster smythiesii prudens* (Forel, 1902)	Himachal Pradesh (186), Jammu & Kashmir (80), Uttarakhand (1)
***Calyptomyrmex***	
*Calyptomyrmex wittmeri* Baroni Urbani, 1975	Kerala (417)
***Cardiocondyla***	
*Cardiocondyla breviscapa* Seifert, 2003 **(E)**	Tamil Nadu (7, 310)
*Cardiocondyla carbonaria* Forel, 1907 **(E)**	Karnataka (327), Maharashtra (114, 194, 310)
*Cardiocondyla emeryi* Forel, 1881 **(I)**	Maharashtra (188), Tamil Nadu (188)
*Cardiocondyla goa* Seifert, 2003 **(E)**	Goa (310), Karnataka (310), Kerala (310)
*Cardiocondyla kagutsuchi* Terayama, 1999	Arunachal Pradesh (1), Assam (1), Himachal Pradesh (310), Nagaland (1), Sikkim (1), West Bengal (1)
*Cardiocondyla mauritanica* Forel, 1890 **(I)**	Arunachal Pradesh (1), Assam (1), Haryana (310), Himachal Pradesh (310), Manipur (1), Meghalaya (1), Mizoram (1), Nagaland (1), Sikkim (1), Tripura (1), West Bengal (1)
*Cardiocondyla minutior* Forel, 1899	Andaman and Nicobar Islands (189, 377), Himachal Pradesh (310), Jammu & Kashmir (1), Maharashtra (194), Uttarakhand (1)
*Cardiocondyla obscurior* Wheeler, 1929	Himachal Pradesh (310)
*Cardiocondyla opaca* Seifert, 2003 **(E)**	Goa (310), Karnataka (310)
*Cardiocondyla parvinoda* Forel, 1902 **(E)**	Kerala (225), Maharashtra (186, 188), Punjab (21), West Bengal (7)
*Cardiocondyla shagrinata* Seifert, 2003 **(E)**	Karnataka (310)
*Cardiocondyla tiwarii* Ghosh, Sheela & Kundu, 2005 **(E)**	Sikkim (1), West Bengal (1)
*Cardiocondyla wroughtonii* (Forel, 1890)	Arunachal Pradesh (1), Assam (1), Bihar (90), Gujarat (188), Himachal Pradesh (1), Jammu & Kashmir (80), Karnataka (262, 265, 287, 288, 362), Kerala (225), Maharashtra (7, 90, 173, 188, 235, 262, 310, 345), Manipur (1), Mizoram (1), Nagaland (1), Sikkim (1), Uttar Pradesh (310), Uttarakhand (1), West Bengal (1)
***Carebara***	
*Carebara aborensis* (Wheeler, 1913) **(E)**	Assam (382)
*Carebara affinis* (Jerdon, 1851)	Andaman and Nicobar Islands (189, 254, 357), Arunachal Pradesh (1), Assam (1), Himachal Pradesh (1), Jammu & Kashmir (80), Karnataka (188, 206, 262, 265, 287, 288, 335, 362), Kerala (188, 225, 249, 262, 287, 305, 335, 343, 352, 357), Maharashtra (188, 206, 249, 262, 287, 335, 343, 352, 357), Manipur (335, 357), Meghalaya (1), Mizoram (1), Rajasthan (334, 335, 343), Sikkim (1), Tamil Nadu (188, 287, 335, 352, 357), Uttarakhand (1), West Bengal (1)
*Carebara asina* (Forel, 1902)	Himachal Pradesh (7), Karnataka (287), Orissa (186, 188), Punjab (214), Uttar Pradesh (214), West Bengal (287, 356)
*Carebara bengalensis* (Forel, 1902)	Sikkim (1), West Bengal (1)
*Carebara carinata* Bharti & Kumar, 2013 **(E)**	Himachal Pradesh (7, 59)
*Carebara dentata* Bharti & Kumar, 2013 **(E)**	Himachal Pradesh (7, 59), Jammu & Kashmir (7, 59), Punjab (59), Uttarakhand (1)
*Carebara diversa* (Jerdon, 1851)	Arunachal Pradesh (1), Assam (1), Goa (411, 412), Himachal Pradesh (1), Karnataka (188, 205, 206, 262, 264, 264, 265, 287, 288, 327, 352, 355, 356), Kerala (188, 205, 206, 262, 287, 305, 319, 329, 352, 355, 383, 386), Maharashtra (115, 129, 188, 205, 206, 262, 287, 319, 352, 355, 356), Mizoram (1), Sikkim (1), Tamil Nadu (255), Uttarakhand (1), West Bengal (1)
*Carebara hornata* Bharti & Kumar, 2013 **(E)**	Himachal Pradesh (7, 59)
*Carebara lamellifrons* (Forel, 1902) **(E)**	Karnataka (186, 188, 352), West Bengal (352)
*Carebara leei* (Forel, 1902) **(E)**	Karnataka (186, 188, 262, 352), West Bengal (352)
*Carebara lignata* Westwood, 1840	Arunachal Pradesh (1), Assam (1), Karnataka (262), Meghalaya (1), West Bengal (205)
*Carebara mukkaliensis* Bharti & Akbar, 2014 **(E)**	Kerala (65)
*Carebara nana* (Roger, 1863)	Arunachal Pradesh (206, 358), Kerala (234)
*Carebara nayana* (Sheela & Narendran, 1997) **(E)**	Karnataka (1), Kerala (322), Orissa (415)
*Carebara obtusidenta* (Xu, 2003)	Arunachal Pradesh (1), Kerala (65), Sikkim (1), West Bengal (1)
*Carebara propomegata* Bharti & Kumar, 2013 **(E)**	Himachal Pradesh (59), Jammu & Kashmir (7, 59), Punjab (59)
*Carebara raja* Forel, 1902	Orissa (1)
*Carebara rectangulata* Bharti & Kumar, 2013 **(E)**	Jammu & Kashmir (7, 59)
*Carebara rectidorsa* (Xu, 2003)	Meghalaya (228)
*Carebara rothneyi* (Forel, 1902) **(E)**	Punjab (21), Sikkim (1), West Bengal (1)
*Carebara similis* (Mayr, 1862)	Andaman and Nicobar Islands (254)
*Carebara spinata* Bharti & Kumar, 2013 **(E)**	Himachal Pradesh (7, 59), Jammu & Kashmir (59), Uttarakhand (1)
*Carebara terayamai* Bharti & Akbar, 2014 **(E)**	Arunachal Pradesh (65), Kerala (65)
*Carebara wroughtonii* (Forel, 1902)	Kerala (225), Orissa (7, 186, 188, 356), West Bengal (356)
***Cataulacus***	
*Cataulacus granulatus* (Latreille, 1802)	Andaman and Nicobar Islands (83, 117, 189, 254), Arunachal Pradesh (1), Assam (1), Himachal Pradesh (83, 188, 192), Tamil Nadu (167)
*Cataulacus latus* Forel, 1891	Arunachal Pradesh (1), Assam (1), Bihar (83), Goa (411), Himachal Pradesh (83), Karnataka (83, 188, 262, 287), Kerala (83, 140, 352), Maharashtra (83, 188), Manipur (1), Mizoram (1), Nagaland (1), Orissa (83, 188, 262, 287, 352, 356), Sikkim (1), Tamil Nadu (219, 262), Uttarakhand (1), West Bengal (1)
*Cataulacus muticus* Emery, 1889	Andaman and Nicobar Islands (254)
*Cataulacus simoni* Emery, 1893	Andaman and Nicobar Islands (83, 114, 189, 254), Haryana (408), Kerala (1), Meghalaya (1)
*Cataulacus taprobanae* Smith, 1853	Andaman and Nicobar Islands (254), Arunachal Pradesh (1), Assam (1), Goa (83, 411, 412), Haryana (408), Himachal Pradesh (1), Jammu & Kashmir (80), Karnataka (7, 83, 188, 256, 262, 264, 265, 287, 288, 362), Kerala (83, 188, 248), Maharashtra (83, 229, 248), Meghalaya (1), Nagaland (1), Sikkim (1), Tamil Nadu (83, 219, 256), Uttarakhand (1), West Bengal (1)
***Crematogaster***	
*Crematogaster abdominalis* Motschoulsky, 1863	India (no state record, 32, 210)
*Crematogaster aberrans* Forel, 1892	Arunachal Pradesh (206), Assam (1), Gujarat (237), Haryana (408), Karnataka (206, 210, 352), Kerala (82, 114, 186, 188, 209, 210, 352), Maharashtra (7, 81, 82, 114, 115, 186, 188, 199, 206, 209, 210, 255, 352), Nagaland (1), Sikkim (1), West Bengal (255, 300, 352)
*Crematogaster aitkenii* Forel, 1902	Arunachal Pradesh (382), Karnataka (186, 188, 210, 352), West Bengal (352)
*Crematogaster anthracina* Smith, 1857	Arunachal Pradesh (1), Assam (1), Haryana (408), Himachal Pradesh (1), Jammu & Kashmir (80), Karnataka (287), Manipur (1), Meghalaya (1), Mizoram (1), Punjab (1), Sikkim (1), Tamil Nadu (1), Uttarakhand (1), West Bengal (1)
*Crematogaster betapicalis* Bolton, 1995 **(E)**	Punjab (210)
*Crematogaster binghamii* Forel, 1904	Arunachal Pradesh (1), Himachal Pradesh (7, 81), Jammu & Kashmir (1), Sikkim (1), Uttarakhand (1), West Bengal (1)
*Crematogaster biroi* Mayr, 1897	Arunachal Pradesh (206), Himachal Pradesh (192, 210), Jammu & Kashmir (80), Karnataka (206, 210, 249, 352, 355), Meghalaya (1), Orissa (415), Punjab (214), Sikkim (1), Uttarakhand (1), West Bengal (1)
*Crematogaster biroi smythiesii* Forel, 1902	Arunachal Pradesh (1), Himachal Pradesh (114, 186, 188, 210), Jammu & Kashmir (1), Sikkim (1), Uttarakhand (1), West Bengal (1)
*Crematogaster brunnea* Smith, 1857	Tamil Nadu (352)
*Crematogaster brunnea contemta* Mayr, 1879	Arunachal Pradesh (1), Assam (1), Gujarat (335, 340, 342), Haryana (335, 340, 342, 351), Himachal Pradesh (335, 340, 342), Karnataka (256), Maharashtra (115), Nagaland (1), Punjab (29, 335, 340, 342), Rajasthan (116, 334, 335, 338, 339, 340, 342, 344), Sikkim (1), Tamil Nadu (256), Uttar Pradesh (335, 340, 342), West Bengal (1)
*Crematogaster brunnea nicevillei* Emery, 1922	Sikkim (1), West Bengal (1)
*Crematogaster brunnea nilgirica* Emery, 1922 **(E)**	Tamil Nadu (114, 188, 210, 352), West Bengal (352)
*Crematogaster brunnea rabula* Forel, 1902	Karnataka (256), Maharashtra (7, 186, 188, 210, 214), Tamil Nadu (256), West Bengal (300)
*Crematogaster brunnea ruginota* Santschi, 1928	Madhya Pradesh (186, 188, 210, 391), West Bengal (186, 188)
*Crematogaster buddhae* Forel, 1902	Andaman and Nicobar Islands (355, 357), Arunachal Pradesh (1), Delhi (1), Kerala (305), Manipur (355, 357), Sikkim (1), West Bengal (1)
*Crematogaster dalyi* Forel, 1902 **(E)**	Haryana (408), Karnataka (262, 287), Tamil Nadu (82, 114, 186, 188, 209, 210, 352), West Bengal (352)
*Crematogaster diffusa* (Jerdon, 1851) **(E)**	Kerala (210, 352), Maharashtra (115)
*Crematogaster dohrni* Mayr, 1879	Haryana (408), Karnataka (202, 262, 287), Kerala (1), Manipur (250), Tamil Nadu (210, 219, 352), Tripura (247, 250)
*Crematogaster dohrni artifex* Mayr, 1879	Andaman and Nicobar Islands (254), Arunachal Pradesh (1), Karnataka (124, 260), Meghalaya (249)
*Crematogaster ebenina* Forel, 1902	Arunachal Pradesh (1), Assam (1), Karnataka (7, 82, 186, 188, 209, 210, 262, 352, 355, 356), Kerala (225), Maharashtra (7, 82, 115, 186, 188, 209, 210, 262, 352, 355), Manipur (1), Nagaland (1), Sikkim (1), West Bengal (1)
*Crematogaster flava* Forel, 1886	Andaman and Nicobar Islands (355), Arunachal Pradesh (1), Assam (1), Himachal Pradesh (7, 81), Jammu & Kashmir (80), Karnataka (188), Kerala (188, 210, 249, 352, 355), Manipur (1), Meghalaya (1), Mizoram (1), Nagaland (1), Orissa (188, 210, 249, 352, 355, 356), Sikkim (1), Tamil Nadu (210, 352, 355), Tripura (1), Uttarakhand (1), West Bengal (1)
*Crematogaster himalayana* Forel, 1902	Himachal Pradesh (7, 186, 188, 192, 210), Manipur (357)
*Crematogaster hogsoni* Forel, 1902	Haryana (351), Karnataka (262, 287), Meghalaya (1)
*Crematogaster inflata* Smith, 1857	Manipur (357)
*Crematogaster kirbii* (Sykes, 1835) **(E)**	Maharashtra (210)
*Crematogaster perelegans* Forel, 1902	Himachal Pradesh (7), Karnataka (256), Maharashtra (186, 188, 210), Punjab (210), Tamil Nadu (256)
*Crematogaster politula* Forel, 1902	Arunachal Pradesh (1), Assam (1), Himachal Pradesh (7), Jammu & Kashmir (80), Meghalaya (1), Sikkim (206, 355), West Bengal (255)
*Crematogaster pradipi* Tiwari, 1999 **(E)**	Tamil Nadu (210, 352)
*Crematogaster ransonneti* Mayr, 1868	Haryana (408), Karnataka (210, 287, 352, 355, 362), Maharashtra (229), Sikkim (1), West Bengal (204, 210, 352)
*Crematogaster rogenhoferi* Mayr, 1879	Andaman and Nicobar Islands (189, 254, 355, 357), Arunachal Pradesh (1), Assam (1), Goa (410, 411, 412), Jammu & Kashmir (67, 80), Karnataka (256, 262, 287), Kerala (210, 248, 249, 352, 355, 357), Maharashtra (115, 194, 210, 248, 249, 352, 355, 357), Manipur (244, 355, 357), Meghalaya (248, 249, 355, 357), Sikkim (1), Tamil Nadu (210, 256, 352, 355, 357), Uttarakhand (1), West Bengal (1)
*Crematogaster rothneyi* Mayr, 1879	Bihar (214), Goa (410, 411, 412), Gujarat (205, 210, 335, 337, 342, 352, 355, 356), Haryana (335, 337, 342), Himachal Pradesh (188, 192, 335, 337, 342), Jammu & Kashmir (1), Jharkhand (214), Karnataka (256, 265, 288, 362), Kerala (188), Maharashtra (115, 129, 188, 205, 210, 214, 229, 248, 249, 287, 335, 337, 342, 352, 355, 356), Meghalaya (1), Orissa (335, 337, 342), Punjab (335, 337, 342), Rajasthan (334, 335, 337, 342), Sikkim (1), Tamil Nadu (140, 188, 205, 210, 219, 256, 335, 337, 342, 352, 355), Uttar Pradesh (335, 337, 342), West Bengal (1)
*Crematogaster rothneyi civa* Forel, 1902	Maharashtra (186, 188, 194, 210), Sikkim (1), West Bengal (1)
*Crematogaster rufa* (Jerdon, 1851) **(E)**	Kerala (210, 352), West Bengal (352)
*Crematogaster sagei* Forel, 1902	Arunachal Pradesh (1), Haryana (355), Himachal Pradesh (7, 81, 186, 188, 192, 210), Jammu & Kashmir (67, 80), Sikkim (1), Uttarakhand (1), West Bengal (1)
*Crematogaster sagei laevinota* Forel, 1902 **(E)**	Himachal Pradesh (186, 188, 210), Madhya Pradesh (186, 188, 210)
*Crematogaster sikkimensis* Forel, 1904 **(E)**	Sikkim (1, 209), West Bengal (1)
*Crematogaster subnuda* Mayr, 1879	Arunachal Pradesh (1), Assam (1), Delhi (1), Goa (410, 411, 412), Gujarat (340, 342), Haryana (31, 340, 342), Himachal Pradesh (7, 31, 81, 340, 342), Jammu & Kashmir (67, 80), Karnataka (124, 125, 262, 287, 340, 342), Maharashtra (31, 229, 340, 342), Meghalaya (228), Mizoram (1), Nagaland (1), Orissa (188), Punjab (31, 79, 214, 340, 342), Sikkim (1), Tamil Nadu (140, 188, 219, 289, 340, 342, 352), Uttar Pradesh (214, 340, 342), Uttarakhand (1), West Bengal (1)
*Crematogaster travancorensis* Forel, 1902	Kerala (114, 186, 188, 210, 249, 352), Manipur (244), Meghalaya (1), West Bengal (352)
*Crematogaster urvijae* Bharti, 2003 **(E)**	Punjab (7, 26, 210)
*Crematogaster walshi* Forel, 1902	Arunachal Pradesh (206), Assam (1), Meghalaya (1), Mizoram (1), Nagaland (1), Orissa (206, 249, 335, 337, 342, 355), Sikkim (1), West Bengal (1)
*Crematogaster wroughtonii* Forel, 1902	Andaman and Nicobar Islands (254, 257), Haryana (408), Karnataka (19, 20, 202, 256, 262, 265, 287, 288), Kerala (305), Maharashtra (186, 188, 210, 262, 352), Tamil Nadu (210, 219, 256, 352), West Bengal (210, 352, 356)
***Dilobocondyla***	
*Dilobocondyla bangalorica* Varghese, 2006 **(E)**	Karnataka (363)
*Dilobocondyla gasteroreticulatus* Bharti & Kumar, 2013 **(E)**	Himachal Pradesh (7, 58), Sikkim (1), Uttarakhand (1)
***Gauromyrmex***	
*Gauromyrmex acanthinus* (Karavaiev, 1935)	Arunachal Pradesh (1), Himachal Pradesh (7), Sikkim (1)
***Indomyrma***	
*Indomyrma dasypyx* Brown, 1986 **(E)**	Karnataka (110), Kerala (7, 110)
***Kartidris***	
*Kartidris nyos* Bolton, 1991	Meghalaya (1), Sikkim (1), West Bengal (1)
***Liomyrmex***	
*Liomyrmex gestroi* (Emery, 1887)	Andaman and Nicobar Islands (254, 296, 353), West Bengal (352)
***Lophomyrmex***	
*Lophomyrmex ambiguus* Rigato, 1994	Arunachal Pradesh (1), Himachal Pradesh (295, 321), Jammu & Kashmir (80), Meghalaya (321), Sikkim (1), Uttarakhand (1), West Bengal (1)
*Lophomyrmex bedoti* Emery, 1893	Arunachal Pradesh (1), Himachal Pradesh (188, 214), Jammu & Kashmir (80), Meghalaya (1), Sikkim (1), Uttarakhand (1), West Bengal (1)
*Lophomyrmex birmanus* Emery, 1893	Arunachal Pradesh (1), Meghalaya (1), Sikkim (321, 355)
*Lophomyrmex changlangensis* Sheela & Ghosh, 2008 **(E)**	Arunachal Pradesh (1)
*Lophomyrmex kali* Rigato, 1994 **(E)**	Arunachal Pradesh (1), Assam (1), West Bengal (204, 321)
*Lophomyrmex quadrispinosus* (Jerdon, 1851)	Arunachal Pradesh (1), Assam (1), Haryana (408), Himachal Pradesh (192), Jammu & Kashmir (80), Karnataka (7, 188, 248, 249, 260, 262, 265, 287, 288, 306, 352, 355, 356, 362), Kerala (248, 249, 262, 287, 349, 352, 355), Maharashtra (188), Meghalaya (1), Mizoram (1), Orissa (188, 248, 249, 262, 287, 295, 352, 355, 356), Sikkim (1), Tamil Nadu (295, 352, 355), Uttar Pradesh (248, 249, 352, 355, 356), Uttarakhand (1), West Bengal (1)
*Lophomyrmex terraceensis* Bharti & Kumar, 2012 **(E)**	Himachal Pradesh (7, 55, 55)
***Lordomyrma***	
*Lordomyrma lakshmi* Taylor, 2012 **(E)**	Kerala (346)
*Lordomyrma taylori* Bharti & Ali, 2013 **(E)**	Kerala (7, 66)
***Mayriella***	
*Mayriella transfuga* Baroni Urbani, 1977	Arunachal Pradesh (1), Himachal Pradesh (122, 317), Jammu & Kashmir (80), Sikkim (1), Uttarakhand (1), West Bengal (1)
*Mayriella warchalowskii* Borowiec, 2007	Arunachal Pradesh (1), Meghalaya (102)
***Meranoplus***	
*Meranoplus bellii* Forel, 1902	Karnataka (7, 186, 188, 262, 307, 352), Kerala (7, 114, 140, 307, 352), Maharashtra (307), Tamil Nadu (352), West Bengal (352)
*Meranoplus bicolor* (Guerin-Meneville, 1844)	Arunachal Pradesh (1), Assam (1), Bihar (7, 214, 307, 357), Delhi (1), Goa (307, 357, 411), Gujarat (335, 340, 342, 357), Haryana (307, 335, 340, 342, 351, 357), Himachal Pradesh (307, 342, 357), Jammu & Kashmir (80), Jharkhand (214, 307), Karnataka (7, 125, 262, 264, 265, 287, 288, 306, 307, 335, 340, 342, 357, 362), Kerala (188, 307, 335, 342, 349, 357), Maharashtra (194, 229, 307, 335, 342, 357), Manipur (1), Meghalaya (1), Mizoram (1), Nagaland (1), Orissa (307, 335, 342, 357), Punjab (29, 79, 214, 307, 335, 340, 342, 357), Rajasthan (116, 307, 333, 334, 335, 338, 340, 342, 344, 357), Sikkim (1), Tamil Nadu (7, 213, 219, 289, 307, 335, 340, 342, 352, 357, 391), Tripura (1), Uttar Pradesh (214, 307, 335, 342, 357), Uttarakhand (1), West Bengal (1)
*Meranoplus laeviventris* Emery, 1889	Meghalaya (1)
*Meranoplus levis* Donisthorpe, 1942	Kerala (1), Tamil Nadu (7, 114, 140, 307, 352), West Bengal (352)
*Meranoplus periyarensis* Bharti & Akbar, 2014 **(E)**	Kerala (62)
*Meranoplus rothneyi* Forel, 1902	Arunachal Pradesh (1), Assam (1), Kerala (7, 186, 188, 206, 249, 307, 352), Manipur (1), Meghalaya (1), Mizoram (1), Sikkim (1), Tamil Nadu (194), Tripura (307), West Bengal (1)
***Messor***	
*Messor himalayanus* (Forel, 1902)	Himachal Pradesh (7, 116, 186, 188, 192, 331, 332, 335, 336, 339, 342, 366), Jammu & Kashmir (67, 80, 186, 188, 190, 192), Kerala (275), Punjab (332, 335, 342), Rajasthan (116, 331, 332, 334, 335, 336, 338, 339, 342, 344)
*Messor instabilis* (Smith, 1858)	Haryana (408), Himachal Pradesh (188), Jammu & Kashmir (80, 386), Madhya Pradesh (188), Maharashtra (188), Punjab (79), Rajasthan (188), Uttar Pradesh (188)
*Messor minor* (Andre, 1883)	Haryana (114, 152, 152)
*Messor semirufus* (Andre, 1883)	Jammu & Kashmir (172)
***Metapone***	
*Metapone nicobarensis* Tiwari & Jonathan, 1986 **(E)**	Andaman and Nicobar Islands (254, 354)
***Monomorium***	
*Monomorium atomum* Forel, 1902	Arunachal Pradesh (1), Assam (1), Goa (411, 412), Karnataka (262, 335, 362), Maharashtra (186, 188, 262, 331, 335), Orissa (186, 188), Punjab (335), Rajasthan (331, 334, 335, 338, 344), Sikkim (1), West Bengal (1)
*Monomorium atomum integrium* Forel, 1902 **(E)**	Maharashtra (186, 188, 194)
*Monomorium biroi* Forel, 1907 **(E)**	Tamil Nadu (194)
*Monomorium carbonarium* (Smith, 1858) **(I)**	Kerala (188)
*Monomorium dichroum* Forel, 1902	Karnataka (186, 188, 265, 287, 288), Maharashtra (7, 186, 188), Tamil Nadu (186, 188, 352), Uttar Pradesh (200), West Bengal (352)
*Monomorium effractor* Bolton, 1987 **(E)**	Maharashtra (92)
*Monomorium floricola* (Jerdon, 1851)	Andaman and Nicobar Islands (92, 254, 357), Arunachal Pradesh (1), Assam (1), Himachal Pradesh (1), Jammu & Kashmir (80), Karnataka (92, 124, 125, 262, 265, 287, 288), Kerala (205, 352, 357, 383, 386), Manipur (1), Meghalaya (1), Mizoram (1), Nagaland (1), Orissa (205, 357), Sikkim (1), Tamil Nadu (205, 289, 352, 357), Tripura (1), West Bengal (1)
*Monomorium indicum* Forel, 1902	Andhra Pradesh (335, 336, 337, 340, 342, 352), Arunachal Pradesh (1), Assam (1), Delhi (1), Gujarat (335, 336, 337, 338, 340, 342, 344), Haryana (335, 336, 337, 340), Himachal Pradesh (342), Jammu & Kashmir (80), Karnataka (262, 265, 287, 288, 306, 335, 336, 337, 340, 342), Maharashtra (7, 194, 195, 287, 331, 335, 336, 337, 340, 342, 352), Manipur (1), Orissa (415), Punjab (29, 79, 214, 287, 331, 335, 336, 337, 340, 342, 352), Rajasthan (116, 331, 332, 334, 335, 335, 336, 337, 338, 339, 340, 342, 344), Sikkim (1), Tamil Nadu (194, 331, 335, 336, 337, 340, 342, 352), Uttar Pradesh (214), West Bengal (1)
*Monomorium indicus* (Smith, 1873) **(E)**	West Bengal (299)
*Monomorium kempi* Mukerjee, 1930 **(E)**	Sikkim (1), West Bengal (1)
*Monomorium latinode* Mayr, 1872	Arunachal Pradesh (1), Assam (1), Karnataka (92, 188, 265, 287, 288), Kerala (294), Maharashtra (188, 214), Manipur (1), Orissa (188, 205, 335, 357), Rajasthan (331, 334, 335, 338, 344), Sikkim (1), Tamil Nadu (92, 194, 205, 289, 335, 352, 357), Uttar Pradesh (3, 326), Uttarakhand (1), West Bengal (1)
*Monomorium longi* Forel, 1902	Arunachal Pradesh (1), Assam (1), Meghalaya (1), Rajasthan (332, 334, 335), Tripura (247, 250, 335)
*Monomorium luisae* Forel, 1904	Jammu & Kashmir (166)
*Monomorium monomorium* Bolton, 1987 **(I)** (see also the dubious records section)	Himachal Pradesh (342), Karnataka (205, 262, 287, 342), Kerala (249, 352), Manipur (244), Meghalaya (249), Tamil Nadu (262), Uttarakhand (1), West Bengal (205, 342, 352)
*Monomorium orientale* Mayr, 1879	Andaman and Nicobar Islands (254), Arunachal Pradesh (1), Assam (1), Himachal Pradesh (1), Jammu & Kashmir (80), Karnataka (125, 188, 287), Manipur (1), Orissa (188), Sikkim (1), Uttarakhand (1), West Bengal (1)
*Monomorium pharaonis* (Linnaeus, 1758) **(I)**	Andaman and Nicobar Islands (254), Arunachal Pradesh (1), Assam (1), Delhi (1), Goa (410, 411, 412), Gujarat (335, 336, 340, 342), Haryana (114, 335, 336, 340, 342), Himachal Pradesh (335, 336, 340, 342), Jammu & Kashmir (80), Karnataka (125, 205, 262, 265, 287, 288, 306, 336, 340, 352), Kerala (92), Maharashtra (129, 229), Manipur (1), Meghalaya (1), Mizoram (1), Nagaland (1), Orissa (415), Punjab (79, 335, 336, 340, 342), Rajasthan (331, 334, 335, 336, 338, 342, 344), Sikkim (1), Tamil Nadu (286), Uttar Pradesh (335, 336, 340, 342), Uttarakhand (1), West Bengal (1)
*Monomorium rugifrons* (Smith, 1858)	India (no further state, 32)
*Monomorium sagei* Forel, 1902	Andaman and Nicobar Islands (254, 257), Himachal Pradesh (186, 188, 192, 257, 272, 332, 342), Jammu & Kashmir (80), Karnataka (335, 342), Rajasthan (332, 334, 335, 342)
*Monomorium schurri* Forel, 1902	Kerala (249, 352), Madhya Pradesh (188), Meghalaya (1), Tamil Nadu (7, 352), West Bengal (352)
*Monomorium subopacum* (Smith, 1858) **(I)**	Karnataka (287, 362), Tamil Nadu (352)
***Myrmecina***	
*Myrmecina pilicornis* Smith, 1858 **(E)**	Maharashtra (114)
*Myrmecina striata* Emery, 1889	Arunachal Pradesh (1), Assam (1), Kerala (305), Meghalaya (1), Sikkim (1), West Bengal (1)
*Myrmecina urbanii* Tiwari, 1994 **(E)**	Karnataka (362), Kerala (350, 352), Orissa (415), Tamil Nadu (1)
*Myrmecina vidyae* Tiwari, 1994 **(E)**	Kerala (350, 352)
***Myrmica***	
*Myrmica adrijae* Bharti, 2012 **(E)**	Himachal Pradesh (7, 34)
*Myrmica aimonissabaudiae* Menozzi, 1939	Arunachal Pradesh (1), Himachal Pradesh (280), Jammu & Kashmir (7, 80, 280), Meghalaya (1), Sikkim (1), West Bengal (1)
*Myrmica cachmiriensis* Forel, 1904	Himachal Pradesh (1), Jammu & Kashmir (7, 80, 190, 190, 192, 280, 283, 375)
*Myrmica curvispinosa* Bharti & Sharma, 2013 **(E)**	Himachal Pradesh (71, 71)
*Myrmica elmesi* Bharti & Sharma, 2011 **(E)**	Jammu & Kashmir (7, 68, 80)
*Myrmica ereptrix* Bolton, 1988 **(E)**	Jammu & Kashmir (7, 93, 280, 282, 283)
*Myrmica foreliana* Radchenko & Elmes, 2001 **(E)**	Jammu & Kashmir (80), Madhya Pradesh (186, 188, 280, 280, 283, 375)
*Myrmica fortior* Forel, 1904 **(E)**	Jammu & Kashmir (7, 80, 190, 192, 280, 281, 283, 375)
*Myrmica hecate* Weber, 1947	Arunachal Pradesh (1), Himachal Pradesh (280, 281), Jammu & Kashmir (280, 280), Sikkim (1), West Bengal (1)
*Myrmica indica* Weber, 1950	Arunachal Pradesh (1), Sikkim (1), West Bengal (1)
*Myrmica inezae* Forel, 1902	Himachal Pradesh (280, 281, 283), Madhya Pradesh (186, 188, 283, 375)
*Myrmica kothiensis* Bharti & Sharma, 2013 **(E)**	Himachal Pradesh (71)
*Myrmica kozlovi* Ruzsky, 1915	Arunachal Pradesh (1), Sikkim (1), West Bengal (1)
*Myrmica longisculpta* Bharti & Sharma, 2011 **(E)**	Jammu & Kashmir (69, 80)
*Myrmica margaritae* Emery, 1889	Meghalaya (1)
*Myrmica nefaria* Bharti, 2012 **(E)**	Himachal Pradesh (7, 33, 33)
*Myrmica nitida* Radchenko & Elmes, 1999 **(E)**	Himachal Pradesh (1), Jammu & Kashmir (7, 80, 279, 280, 283)
*Myrmica ordinaria* Radchenko & Elmes, 1999	Jammu & Kashmir (7, 80, 279, 280, 283)
*Myrmica pachei* Forel, 1906	Arunachal Pradesh (1), Sikkim (1), West Bengal (1)
*Myrmica petita* Radchenko & Elmes, 1999 **(E)**	Jammu & Kashmir (279, 280, 283)
*Myrmica radchenkoi* Bharti & Sharma, 2011 **(E)**	Jammu & Kashmir (70, 80)
*Myrmica religiosa* Bharti & Gul, 2013 **(E)**	Uttarakhand (1)
*Myrmica rhytida* Radchenko & Elmes, 1999 **(E)**	Himachal Pradesh (1), Jammu & Kashmir (7, 80, 279, 280, 283)
*Myrmica ritae* Emery, 1889	Sikkim (355)
*Myrmica rugosa* Mayr, 1865	Arunachal Pradesh (1), Himachal Pradesh (1), Jammu & Kashmir (67, 80, 280), Madhya Pradesh (188), Sikkim (1), Uttarakhand (1), West Bengal (1)
*Myrmica rupestris* Forel, 1902	Arunachal Pradesh (1), Himachal Pradesh (7, 186, 188, 280, 283, 375), Jammu & Kashmir (80, 190, 280), Sikkim (1), West Bengal (1)
*Myrmica smythiesii* Forel, 1902	Himachal Pradesh (186, 190, 280, 281, 283, 375), Jammu & Kashmir (67, 80), Uttarakhand (1)
*Myrmica urbanii* Radchenko & Elmes, 1998	Arunachal Pradesh (1), Meghalaya (1)
*Myrmica varisculpta* Radchenko & Elmes, 2009 **(E)**	Jammu & Kashmir (7, 80, 283, 284)
*Myrmica wardi* Radchenko & Elmes, 1999	Himachal Pradesh (279, 280, 283), Jammu & Kashmir (7, 80, 279, 280, 283)
*Myrmica weberi* Elmes & Radchenko, 2009	Bihar (151, 283), West Bengal (151, 283)
*Myrmica williamsi* Radchenko & Elmes, 1999 **(E)**	Jammu & Kashmir (7, 279, 280, 283)
*Myrmica wittmeri* Radchenko & Elmes, 1999	Himachal Pradesh (279, 280, 283), Jammu & Kashmir (80)
***Myrmicaria***	
*Myrmicaria brunnea* Saunders, 1842	Arunachal Pradesh (1), Assam (1), Bihar (214), Goa (410, 411, 412), Himachal Pradesh (1), Jammu & Kashmir (80), Jharkhand (214), Karnataka (260, 262, 264, 265, 287, 288, 291, 306, 362), Kerala (8, 140, 225, 301, 352, 369), Maharashtra (115, 188), Manipur (1), Meghalaya (1), Punjab (79), Sikkim (1), Tamil Nadu (7, 140, 213, 219, 256, 289, 352), Uttarakhand (1), West Bengal (1)
*Myrmicaria brunnea subcarinata* (Smith, 1857)	West Bengal (170)
*Myrmicaria carinata* (Smith, 1857)	Karnataka (7)
*Myrmicaria fodica* (Jerdon, 1851)	Arunachal Pradesh (1), Assam (1), Sikkim (1), Tamil Nadu (7), West Bengal (1)
***Paratopula***	
*Paratopula andamanensis* (Forel, 1903) (E)	Andaman and Nicobar Islands (94, 114, 189, 254)
*Paratopula ceylonica* (Emery, 1901)	Karnataka (1), Orissa (94, 186, 188, 320), Uttar Pradesh (320), West Bengal (94, 114, 186, 188, 300, 320, 356)
*Paratopula intermedia* Sheela & Narendran, 1998 **(E)**	Kerala (320, 323)
***Perissomyrmex***	
*Perissomyrmex monticola* Baroni Urbani & De Andrade, 1993	West Bengal (1)
***Pheidole***	
*Pheidole allani* Bingham, 1903	Meghalaya (249)
*Pheidole asperata* Emery, 1895	Gujarat (1), Karnataka (1), Kerala (1), Maharashtra (1), Tamil Nadu (1)
*Pheidole bandata* Bharti, 2004 **(E)**	Himachal Pradesh (30)
*Pheidole binghamii* Forel, 1902	Jammu & Kashmir (80)
*Pheidole capellinii* Emery, 1887	Andaman and Nicobar Islands (254), Meghalaya (1)
*Pheidole constanciae* Forel, 1902	Kerala (273), Meghalaya (1), Tamil Nadu (185, 186, 213, 249, 259, 273, 352), West Bengal (352)
*Pheidole constanciae nigra* Forel, 1902 **(E)**	Tamil Nadu (185, 186)
*Pheidole coonoorensis* Forel, 1902 **(E)**	Tamil Nadu (145, 185, 186)
*Pheidole diffusa* (Jerdon, 1851) **(E)**	Arunachal Pradesh (1), Assam (1), Sikkim (1), West Bengal (1)
*Pheidole duneraensis* Bharti, 2001 **(E)**	Himachal Pradesh (22)
*Pheidole feae* Emery, 1895	Meghalaya (249)
*Pheidole fergusoni* Forel, 1902	Kerala (114, 185, 186, 352), Tamil Nadu (213, 259), West Bengal (352)
*Pheidole fervens* Smith, 1858	Arunachal Pradesh (1), Assam (1), Himachal Pradesh (145, 145, 185, 186, 192), Jammu & Kashmir (80), Sikkim (1), West Bengal (1)
*Pheidole ghatica* Forel, 1902 **(E)**	Kerala (273), Maharashtra (7, 185, 186, 213, 259)
*Pheidole grayi* Forel, 1902 **(E)**	Goa (410), Maharashtra (185, 186, 213, 259), Sikkim (1)
*Pheidole horni* Emery, 1901	Manipur (357)
*Pheidole hospita* Bingham, 1903 **(E)**	Sikkim (1), West Bengal (1)
*Pheidole indica* Mayr, 1879	Andaman and Nicobar Islands (189, 254), Arunachal Pradesh (1), Assam (1), Bihar (214), Delhi (1), Himachal Pradesh (7, 48, 145, 185, 186, 190, 192), Jammu & Kashmir (67, 80, 185, 186, 192, 273, 355), Jharkhand (214), Karnataka (185, 186), Kerala (185, 186, 273), Maharashtra (115, 145, 185, 186, 214), Meghalaya (249), Mizoram (1), Nagaland (1), Orissa (145, 185, 186), Punjab (79, 214), Sikkim (1), Tamil Nadu (219), Tripura (1), Uttarakhand (1), West Bengal (1)
*Pheidole jucunda* Forel, 1885	Arunachal Pradesh (1), Assam (1), Jammu & Kashmir (80), Maharashtra (185, 186), Meghalaya (1), Nagaland (1), Sikkim (1), West Bengal (1)
*Pheidole jucunda fossulata* Forel, 1902	Himachal Pradesh (1), Jammu & Kashmir (80), Maharashtra (7, 186), Sikkim (192, 355), Uttarakhand (1)
*Pheidole lamellinoda* Forel, 1902	Delhi (1), Maharashtra (115, 185, 186), Meghalaya (1)
*Pheidole lanuginosa* Wilson, 1984 **(E)**	Arunachal Pradesh (1), Assam (1)
*Pheidole latinoda* Roger, 1863	Arunachal Pradesh (1), Assam (1), Bihar (214), Delhi (1), Himachal Pradesh (7), Jharkhand (214), Maharashtra (185, 186, 214), Manipur (1), Mizoram (1), Nagaland (1), Punjab (29, 214), Sikkim (1), Tamil Nadu (219, 289), Tripura (1), Uttar Pradesh (214), West Bengal (1)
*Pheidole latinoda angustior* Forel, 1902	Delhi (1), Jammu & Kashmir (80), Maharashtra (185, 186, 194, 213, 259), Punjab (79)
*Pheidole latinoda major* Forel, 1885 **(E)**	Jammu & Kashmir (80), Punjab (1), Uttarakhand (1), West Bengal (114, 170, 185, 186)
*Pheidole malabarica* (Jerdon, 1851) **(E)**	West Bengal (352)
*Pheidole malinsii* Forel, 1902	Haryana (408), Meghalaya (249, 355), Sikkim (249, 352, 355), Tamil Nadu (352, 355), West Bengal (7)
*Pheidole megacephala* (Fabricius, 1793) **(I)**	Andaman and Nicobar Islands (189, 254)
*Pheidole minor* (Jerdon, 1851) **(E)**	Kerala (352), West Bengal (352)
*Pheidole multidens* Forel, 1902	Karnataka (287), Maharashtra (7, 185, 186), Uttar Pradesh (319), West Bengal (319)
*Pheidole mus* Forel, 1902	Karnataka (185, 186, 249, 352, 356), Maharashtra (214), Meghalaya (1), Sikkim (1), West Bengal (1)
*Pheidole naoroji* Forel, 1902 **(E)**	Maharashtra (185, 186)
*Pheidole noda* Smith, 1874	Andaman and Nicobar Islands (189, 254), Arunachal Pradesh (1), Assam (1), Goa (1), Himachal Pradesh (185, 186, 192), Karnataka (185, 186), Kerala (185, 186), Maharashtra (185, 186, 194), Manipur (1), Orissa (185, 186), Sikkim (1), Tamil Nadu (256), Tripura (1), West Bengal (1)
*Pheidole parasitica* Wilson, 1984 **(E)**	Arunachal Pradesh (1), Assam (1), Kerala (400)
*Pheidole parva* Mayr, 1865	Arunachal Pradesh (1), Himachal Pradesh (147, 148), Jammu & Kashmir (1), Karnataka (185, 186, 265, 288), Kerala (148, 185, 186), Maharashtra (185, 186), Meghalaya (1), Uttar Pradesh (147), Uttarakhand (1), West Bengal (300)
*Pheidole phipsoni* Forel, 1902	Karnataka (7, 185, 186, 352), Maharashtra (194), Tamil Nadu (194, 352)
*Pheidole pronotalis* Forel, 1902	Himachal Pradesh (1), Meghalaya (1), Sikkim (1)
*Pheidole providens* (Sykes, 1835)	Maharashtra (330), West Bengal (352)
*Pheidole roberti* Forel, 1902	Gujarat (338, 340, 344), Karnataka (185, 186, 205, 249, 340, 352, 355, 356), Kerala (1), Maharashtra (340), Meghalaya (1), Sikkim (1), Tamil Nadu (1), West Bengal (204, 205, 266, 340, 352, 355, 356)
*Pheidole rogersi* Forel, 1902	Arunachal Pradesh (1), Assam (185, 186, 192), Sikkim (1), West Bengal (1)
*Pheidole rogersi taylori* Forel, 1902	Orissa (185, 186), West Bengal (114)
*Pheidole sagei* Forel, 1902	Himachal Pradesh (7, 185, 186, 192, 249), Jammu & Kashmir (80), Meghalaya (1), Uttarakhand (1)
*Pheidole sharpi* Forel, 1902	Goa (411, 412), Himachal Pradesh (1), Jammu & Kashmir (80), Karnataka (185, 186, 265, 287), Kerala (274), Maharashtra (185, 186), Tamil Nadu (185, 186, 352), Uttarakhand (1)
*Pheidole sharpi hoogwerfi* Forel, 1902	Karnataka (352), Maharashtra (114, 115, 185, 186, 352), West Bengal (352)
*Pheidole singaporensis* Ozdikmen, 2010	Andaman and Nicobar Islands (254, 254), Jammu & Kashmir (80)
*Pheidole smythiesii* Forel, 1902	Arunachal Pradesh (1), Assam (1), Haryana (408), Himachal Pradesh (147), Jammu & Kashmir (80), Meghalaya (1), Sikkim (249, 355, 355), Uttarakhand (1), West Bengal (7, 147, 300, 355)
*Pheidole spathifera* Forel, 1902	Andhra Pradesh (352), Assam (262, 319, 351, 352, 356), Delhi (1), Haryana (351), Jammu & Kashmir (80), Karnataka (262, 265, 287, 288, 306, 319, 362), Kerala (185, 186, 225, 319, 352), Tamil Nadu (167, 168, 185, 186, 213, 259, 293, 319, 352), West Bengal (300, 319, 351, 352, 356)
*Pheidole spathifera aspatha* Forel, 1902 **(E)**	Assam (114, 185, 186), Delhi (1), Himachal Pradesh (1), Jammu & Kashmir (80), Kerala (185, 186), Punjab (79)
*Pheidole spathifera yerburyi* Forel, 1902	Tamil Nadu (194)
*Pheidole sulcaticeps* Roger, 1863	Gujarat (335, 338, 340, 344), Maharashtra (185, 186), Orissa (185, 186), Rajasthan (116, 334, 335, 338, 339, 344), Tamil Nadu (1), West Bengal (114, 335, 339, 340, 356)
*Pheidole sulcaticeps punensis* Forel, 1902 **(E)**	Maharashtra (185, 186)
*Pheidole sykesii* Forel, 1902 **(E)**	Arunachal Pradesh (1), Assam (1), Karnataka (287), Maharashtra (185, 186, 213, 259), Sikkim (1), West Bengal (1)
*Pheidole templaria* Forel, 1902	Assam (185, 186)
*Pheidole terraceensis* Bharti, 2001 **(E)**	Himachal Pradesh (22)
*Pheidole vulgaris* Eguchi, 2006	Uttar Pradesh (146, 147, 148)
*Pheidole watsoni* Forel, 1902	Andaman and Nicobar Islands (254), Arunachal Pradesh (206), Haryana (408), Jammu & Kashmir (80), Karnataka (265, 287, 288), Maharashtra (229), Meghalaya (249), Orissa (185, 186), West Bengal (186, 249, 287, 300, 356)
*Pheidole woodmasoni* Forel, 1885	Arunachal Pradesh (1), Assam (1), Delhi (1), Himachal Pradesh (185, 186, 192), Jammu & Kashmir (80), Karnataka (185, 262, 265, 287, 288, 306, 362), Maharashtra (185, 186), Meghalaya (1), Orissa (185, 186), Punjab (214), Sikkim (1), Tamil Nadu (185, 186), Uttarakhand (1), West Bengal (1)
*Pheidole wroughtonii* Forel, 1902 **(E)**	Gujarat (335, 338, 340, 344), Karnataka (185, 186, 262, 335, 339, 340), Maharashtra (185, 186), Rajasthan (331, 334, 335, 338, 339, 344), Uttar Pradesh (7)
***Pristomyrmex***	
*Pristomyrmex brevispinosus* Emery, 1887	Assam (382)
*Pristomyrmex sulcatus* Emery, 1895	Sikkim (1)
***Recurvidris***	
*Recurvidris pickburni* Bolton, 1992	Uttar Pradesh (122)
*Recurvidris recurvispinosa* (Forel, 1890)	Arunachal Pradesh (1), Assam (1), Himachal Pradesh (96, 205, 324, 342), Jammu & Kashmir (80), Karnataka (205, 262, 265, 287, 288, 342, 362), Kerala (96, 205, 324, 342), Maharashtra (96, 114, 173, 188, 248, 249, 262, 388, 391), Manipur (1), Meghalaya (1), Orissa (415), Punjab (1), Sikkim (1), Tamil Nadu (96, 205, 324, 342), Uttarakhand (1), West Bengal (1)
***Rhopalomastix***	
*Rhopalomastix rothneyi* Forel, 1900	Karnataka (122), Sikkim (1), West Bengal (1)
***Solenopsis***	
*Solenopsis geminata* (Fabricius, 1804) **(I)**	Andaman and Nicobar Islands (114, 117, 189, 254, 340, 355, 357), Arunachal Pradesh (1), Assam (1), Bihar (214, 360), Goa (7, 410, 411, 412), Gujarat (335, 340), Himachal Pradesh (1), Jammu & Kashmir (80), Jharkhand (214, 360), Karnataka (7, 125, 205, 214, 260, 262, 265, 288, 306, 335, 340, 352, 355, 357, 362), Kerala (205, 225, 335, 340, 352, 355, 357), Maharashtra (214, 229), Manipur (205, 355, 357), Meghalaya (205, 355, 357), Mizoram (1), Nagaland (1), Orissa (205, 335, 355, 357), Punjab (21, 335, 340), Rajasthan (334, 335, 340, 343), Sikkim (1), Tamil Nadu (112, 205, 219, 286, 293, 335, 340, 352, 355, 357), Tripura (1), West Bengal (1)
*Solenopsis nitens* Bingham, 1903	Karnataka (125), Kerala (305)
***Stenamma***	
*Stenamma jhitingriense* Bharti, Gul & Sharma, 2012 **(E)**	Himachal Pradesh (7, 74)
*Stenamma kashmirense* Baroni Urbani, 1977	Himachal Pradesh (103), Jammu & Kashmir (7, 11, 143, 242)
*Stenamma wilsoni* Bharti, Gul & Sharma, 2012 **(E)**	Himachal Pradesh (7, 74)
***Strumigenys***	
*Strumigenys aduncomala* De Andrade, 2007 **(E)**	Arunachal Pradesh (1), Meghalaya (16)
*Strumigenys assamensis* Baroni Urbani & De Andrade, 1994 **(E)**	Arunachal Pradesh (1), Meghalaya (7, 15)
*Strumigenys emmae* (Emery, 1890) **(I)**	Arunachal Pradesh (1), Assam (1), Gujarat (1), Karnataka (361, 362), Kerala (1), Maharashtra (1), Manipur (1), Sikkim (1), West Bengal (1)
*Strumigenys exilirhina* Bolton, 2000	Arunachal Pradesh (1), Assam (1), Nagaland (1), Sikkim (1), Tripura (1), Uttar Pradesh (1), Uttarakhand (1), West Bengal (1)
*Strumigenys fixata* Bolton, 2000 **(E)**	Karnataka (1), Kerala (99), Maharashtra (1), Tamil Nadu (7)
*Strumigenys godeffroyi* Mayr, 1866	Karnataka (262), Kerala (1), Maharashtra (186, 188), Meghalaya (1), Sikkim (1), West Bengal (352)
*Strumigenys habropilosa* Bolton, 2000 **(E)**	Kerala (1), Tamil Nadu (7)
*Strumigenys hemisobek* (Bolton, 2000)	Kerala (61), Sikkim (1)
*Strumigenys hostilis* Bolton, 2000 **(E)**	Goa (7, 99), Karnataka (7, 99)
*Strumigenys hypoturba* Bolton, 2000 **(E)**	Kerala (99), Tamil Nadu (7)
*Strumigenys lyroessa* (Roger, 1862)	Arunachal Pradesh (1), Assam (1), Goa (7, 99), Gujarat (1), Karnataka (7, 99), Kerala (99), Maharashtra (1), Nagaland (1), Sikkim (1), Tamil Nadu (1), West Bengal (1)
*Strumigenys membranifera* Emery, 1869 **(I)**	Arunachal Pradesh (1), Assam (1), Himachal Pradesh (1), Jammu & Kashmir (1), Mizoram (1), Sikkim (1), Uttarakhand (1), West Bengal (1)
*Strumigenys mitis* (Brown, 2000)	Arunachal Pradesh (1), Assam (1), Kerala (61), Mizoram (1), Sikkim (1), West Bengal (1)
*Strumigenys mukkaliensis* Bharti & Akbar, 2013 **(E)**	Kerala (61)
*Strumigenys mutica* (Brown, 1949)	Kerala (61)
*Strumigenys nannosobek* (Bolton, 2000)	Kerala (61), Sikkim (1)
*Strumigenys nanzanensis* Lin & Wu, 1996	Sikkim (1), West Bengal (1)
*Strumigenys nepalensis* Baroni Urbani & De Andrade, 1994	Arunachal Pradesh (1), Assam (1), Himachal Pradesh (122), Manipur (1), Meghalaya (15), Mizoram (1), Sikkim (1), Uttarakhand (1), West Bengal (1)
*Strumigenys peraucta* Bolton, 2000 **(E)**	Goa (7, 99), Karnataka (7, 99)
*Strumigenys podarge* (Bolton, 2000)	Himachal Pradesh (122)
*Strumigenys rogeri* Emery, 1890 **(I)**	Kerala (61)
*Strumigenys smythiesii* Forel, 1902 **(E)**	Arunachal Pradesh (1), Assam (1), Himachal Pradesh (1), Kerala (225)
*Strumigenys thanikkudyensis* Bharti & Akbar, 2013 **(E)**	Kerala (61)
*Strumigenys virgila* Bolton, 2000	Arunachal Pradesh (1), Assam (1), Himachal Pradesh (7, 99), Sikkim (1), Uttar Pradesh (1), West Bengal (1)
***Temnothorax***	
*Temnothorax desioi* (Menozzi, 1939)	Himachal Pradesh (1), Jammu & Kashmir (80), Uttarakhand (1)
*Temnothorax desioi melanicus* (Menozzi, 1939) **(E)**	Himachal Pradesh (1), Jammu & Kashmir (7, 80)
*Temnothorax fultonii* (Forel, 1902)	Himachal Pradesh (186, 188, 192), Jammu & Kashmir (80)
*Temnothorax himachalensis* Bharti, Gul & Schulz, 2012 **(E)**	Himachal Pradesh (7, 72), Jammu & Kashmir (72)
*Temnothorax inermis* (Forel, 1902) **(E)**	Himachal Pradesh (186, 188, 192)
*Temnothorax kashmirensis* Bharti, Gul & Schulz, 2012 **(E)**	Himachal Pradesh (7, 72), Jammu & Kashmir (7, 72)
*Temnothorax microreticulatus* Bharti, Gul & schulz, 2012 **(E)**	Himachal Pradesh (7, 72)
*Temnothorax nordmeyeri* (Schulz, 1997) **(E)**	Goa (308), Karnataka (308)
*Temnothorax rothneyi* (Forel, 1902) **(E)**	Himachal Pradesh (186, 188), Jammu & Kashmir (80), Kerala (225), Madhya Pradesh (186, 188, 355), Meghalaya (228), Sikkim (114, 192, 355), Uttarakhand (1)
*Temnothorax rothneyi simlensis* (Forel, 1904) **(E)**	Himachal Pradesh (190, 192)
*Temnothorax schurri* (Forel, 1902) **(E)**	Himachal Pradesh (1), Madhya Pradesh (186, 188)
*Temnothorax wroughtonii* (Forel, 1904) **(E)**	Jammu & Kashmir (190, 192)
***Tetramorium***	
*Tetramorium barryi* Mathew, 1981 **(E)**	Arunachal Pradesh (1), Meghalaya (1), Sikkim (1)
*Tetramorium beesoni* (Mukerjee, 1934) **(E)**	Karnataka (256), Tamil Nadu (256)
*Tetramorium belgaense* Forel, 1902 **(E)**	Goa (7), Karnataka (7, 87, 88, 186, 188, 352), West Bengal (352)
*Tetramorium bicarinatum* (Nylander, 1846) **(I)**	Andaman and Nicobar Islands (87, 248, 249, 254, 355), Arunachal Pradesh (248, 249), Assam (87, 248, 249, 355), Himachal Pradesh (1), Karnataka (87, 248, 249, 262, 265), Maharashtra (229), Meghalaya (248, 249, 355), Sikkim (355), Uttarakhand (1)
*Tetramorium browni* Bolton, 1980	Arunachal Pradesh (1)
*Tetramorium caldarium* (Roger, 1857) **(I)**	Punjab (56), Rajasthan (89)
*Tetramorium christiei* Forel, 1902	Meghalaya (1), Sikkim (1), West Bengal (1)
*Tetramorium coonoorense* Forel, 1902 **(E)**	Himachal Pradesh (1), Kerala (352), Tamil Nadu (7, 86, 186, 188, 352), Uttarakhand (1), West Bengal (352)
*Tetramorium cordatum* Sheela & Narendran, 1998 **(E)**	Kerala (325)
*Tetramorium decamerum* (Forel, 1902) **(E)**	Karnataka (86, 114, 186, 188, 256, 262, 352), Tamil Nadu (256), West Bengal (352)
*Tetramorium elisabethae* Forel, 1904 **(E)**	Jammu & Kashmir (7, 87, 190, 192)
*Tetramorium fergusoni* Forel, 1902 **(E)**	Kerala (7, 87, 186, 188, 352), West Bengal (352)
*Tetramorium indicum* Forel, 1913	Andaman and Nicobar Islands (87, 254), Kerala (87), Sikkim (1), West Bengal (1)
*Tetramorium inglebyi* Forel, 1902	Goa (1), Gujarat (1), Karnataka (287), Kerala (7, 87, 186, 188, 287, 352), Maharashtra (1), Tamil Nadu (1), West Bengal (352)
*Tetramorium keralense* Sheela & Narendran, 1998 **(E)**	Kerala (325)
*Tetramorium kheperra* (Bolton, 1976)	Assam (86)
*Tetramorium lanuginosum* Mayr, 1870	Andaman and Nicobar Islands (254), Arunachal Pradesh (1), Assam (1), Delhi (1), Goa (7), Gujarat (206, 355), Haryana (1), Himachal Pradesh (1), Jammu & Kashmir (80), Karnataka (7, 214), Kerala (86, 249), Maharashtra (86, 249), Meghalaya (1), Orissa (86, 188, 208, 249, 356), Punjab (214), Sikkim (1), Uttarakhand (1), West Bengal (1)
*Tetramorium malabarense* Sheela & Narendran, 1998 **(E)**	Kerala (325)
*Tetramorium mayri* (Forel, 1912)	Maharashtra (7, 88, 91, 106, 114, 400)
*Tetramorium meghalayense* Bharti, 2011 **(E)**	Meghalaya (1)
*Tetramorium mixtum* Forel, 1902	Goa (7), Karnataka (7, 262, 287, 362), Kerala (87, 249, 352), Meghalaya (1), Tamil Nadu (7, 87, 114, 186, 188, 249, 352), West Bengal (352)
*Tetramorium myops* Bolton, 1977 **(E)**	Chhattisgarh (7, 87), Kerala (1), Madhya Pradesh (7, 87, 87)
*Tetramorium nursei* Bingham, 1903	Haryana (408), Kerala (305)
*Tetramorium obesum* Andre, 1887	Arunachal Pradesh (1), Assam (1), Himachal Pradesh (1), Karnataka (125, 188, 256, 352, 356), Kerala (86, 188), Maharashtra (115, 352, 356), Sikkim (1), Tamil Nadu (188, 256), West Bengal (1)
*Tetramorium pacificum* Mayr, 1870 **(I)**	Andaman and Nicobar Islands (254, 355), Arunachal Pradesh (1), Assam (1), Karnataka (327), Kerala (1), Manipur (1), Mizoram (1), Sikkim (1), West Bengal (1)
*Tetramorium petiolatum* Sheela & Narendran, 1998 **(E)**	Kerala (325)
*Tetramorium pilosum* Emery, 1893	Haryana (408)
*Tetramorium rossi* (Bolton, 1976) **(E)**	Kerala (7, 86)
*Tetramorium rugigaster* Bolton, 1977 **(E)**	Karnataka (287), Kerala (7, 87, 287)
*Tetramorium salvatum* Forel, 1902	Gujarat (335, 340, 342), Himachal Pradesh (342), Rajasthan (334, 335, 338, 339, 340, 342, 344)
*Tetramorium scabrum* Mayr, 1879	Sikkim (192)
*Tetramorium sentosum* Sheela & Narendran, 1998 **(E)**	Kerala (325)
*Tetramorium shivalikense* Bharti & Kumar, 2012 **(E)**	Himachal Pradesh (56), Punjab (56), Uttarakhand (1)
*Tetramorium simillimum* (Smith, 1851) **(I)**	Arunachal Pradesh (1), Assam (1), Bihar (214), Goa (7), Himachal Pradesh (1), Jammu & Kashmir (80), Jharkhand (214), Karnataka (7), Maharashtra (188), Manipur (1), Meghalaya (1), Punjab (87, 214, 249), Sikkim (1), West Bengal (1)
*Tetramorium smithi* Mayr, 1879	Arunachal Pradesh (1), Assam (1), Bihar (214), Goa (7), Haryana (21), Himachal Pradesh (21), Jammu & Kashmir (80), Jharkhand (214), Karnataka (7, 87, 125, 188, 262, 362), Kerala (87, 225, 249), Maharashtra (87, 229, 249), Meghalaya (1), Punjab (21), Sikkim (1), West Bengal (1)
*Tetramorium tonganum* Mayr, 1870 **(I)**	Himachal Pradesh (56), Uttarakhand (1)
*Tetramorium tortuosum* Roger, 1863	Karnataka (19, 20, 87, 114, 186, 188, 262, 352), Kerala (87), Meghalaya (249), Sikkim (355), Tamil Nadu (87), West Bengal (352)
*Tetramorium triangulatum* Bharti & Kumar, 2012 **(E)**	Himachal Pradesh (56), Punjab (56), Uttarakhand (1)
*Tetramorium urbanii* Bolton, 1977	Meghalaya (56, 228), Sikkim (1)
*Tetramorium walshi* (Forel, 1890)	Arunachal Pradesh (1), Assam (1), Bihar (214), Delhi (1), Himachal Pradesh (262, 319, 333, 335, 342, 343), Jammu & Kashmir (80), Jharkhand (214), Karnataka (125, 188, 262, 265, 287, 288, 319, 333, 335, 342, 343, 362), Kerala (7, 188, 249, 319, 335, 342, 352), Maharashtra (173, 188), Manipur (1), Meghalaya (1), Nagaland (1), Orissa (249, 319, 335, 342), Punjab (79, 214), Rajasthan (333, 334, 335, 342, 343), Sikkim (1), Tamil Nadu (114, 186, 188, 219, 249, 256, 289, 319, 335, 342, 352), Uttar Pradesh (214, 319), West Bengal (1)
*Tetramorium wroughtonii* (Forel, 1902)	Arunachal Pradesh (1), Assam (1), Goa (410), Gujarat (340), Himachal Pradesh (21, 21), Karnataka (1,7, 86, 91, 107, 186, 188, 248, 249, 340, 345, 352, 355, 362, 391), Kerala (1, 225), Maharashtra (340), Manipur (1), Meghalaya (1), Mizoram (1), Nagaland (1), Sikkim (1), Tamil Nadu (219), Tripura (355), West Bengal (1)
*Tetramorium yerburyi* Forel, 1902	Kerala (305), Tamil Nadu (352)
***Trichomyrmex***	
*Trichomyrmex aberrans* (Forel, 1902)	Arunachal Pradesh (206), Haryana (408), Himachal Pradesh (1), Jammu & Kashmir (80), Madhya Pradesh (186, 188, 206), Meghalaya (249), Uttarakhand (1)
*Trichomyrmex criniceps* (Mayr, 1879)	Arunachal Pradesh (1), Assam (1), Gujarat (335, 340), Haryana (335, 340), Karnataka (7, 186, 188, 262, 277, 285, 287, 335, 340, 352, 362), Maharashtra (115, 186, 188, 277, 285, 352), Punjab (79), Rajasthan (331, 334, 335, 338, 340, 344), Sikkim (1), Tamil Nadu (277), West Bengal (1)
*Trichomyrmex destructor* (Jerdon, 1851) **(I)**	Andaman and Nicobar Islands (92, 189, 254), Delhi (1), Goa (410, 411, 412), Gujarat (335, 340, 342), Haryana (122, 335, 340, 342), Himachal Pradesh (190, 335, 340, 342), Jammu & Kashmir (80), Karnataka (262, 265, 287, 288, 335, 340, 342, 362), Kerala (92, 335), Maharashtra (194, 194), Punjab (79, 335, 340, 342), Rajasthan (334, 335, 338, 339, 340, 340, 342, 344), Tamil Nadu (122, 256, 289, 335, 340), Uttar Pradesh (335, 340, 342), Uttarakhand (1), West Bengal (92, 205, 335, 342, 352)
*Trichomyrmex glaber* (Andre, 1883)	Arunachal Pradesh (1), Assam (1), Gujarat (335, 338, 340, 344), Himachal Pradesh (1), Jammu & Kashmir (80), Karnataka (186, 188, 214, 277, 287, 352, 362), Kerala (7), Maharashtra (114, 186, 188, 277, 352), Mizoram (1), Punjab (79, 335, 340), Rajasthan (331, 334, 335, 338, 339, 340, 344), Sikkim (1), Tamil Nadu (140, 277, 335, 340, 352), Uttarakhand (1), West Bengal (1)
*Trichomyrmex mayri* (Forel, 1902)	Gujarat (337), Karnataka (7), Kerala (337, 352), Maharashtra (7, 194), Rajasthan (331, 334, 337, 338, 339, 344), Tamil Nadu (92, 337, 352)
*Trichomyrmex scabriceps* (Mayr, 1879)	Arunachal Pradesh (1), Assam (1), Bihar (214), Delhi (1), Goa (1, 214), Gujarat (335, 336, 340), Haryana (335), Himachal Pradesh (1), Jammu & Kashmir (80), Karnataka (186, 188, 260, 262, 265, 277, 287, 288, 306, 335, 336, 340, 352), Kerala (285, 319, 335, 336, 339, 340, 352), Maharashtra (188), Orissa (335), Punjab (214, 319, 335, 336, 339, 340, 352), Rajasthan (334, 335, 336, 338, 339, 340, 344), Sikkim (1), Tamil Nadu (140, 289, 335, 336, 340, 352), Uttarakhand (1), West Bengal (1)
*Trichomyrmex wroughtoni* Forel, 1902	Goa (1), Gujarat (335, 340, 342), Haryana (335, 342), Himachal Pradesh (335, 342), Karnataka (7, 186, 188, 197, 262, 331, 335, 342, 352), Kerala (225), Maharashtra (186, 188, 194, 195, 331, 335, 340, 342, 352), Punjab (335, 342), Rajasthan (331, 334, 335, 338, 340, 342, 344), Uttar Pradesh (335, 342), West Bengal (352)
***Tyrannomyrmex***	
*Tyrannomyrmex dux* Borowiec, 2007 **(E)**	Kerala (7, 101)
***Vollenhovia***	
*Vollenhovia gastropunctata* Bharti & Kumar, 2013 **(E)**	Himachal Pradesh (57)
*Vollenhovia oblonga laevithorax* Emery, 1889	Andaman and Nicobar Islands (189, 254)
*Vollenhovia penetrans* (Smith, 1857)	Andaman and Nicobar Islands (7)
***Vombisidris***	
*Vombisidris humboldticola* Zacharias & Rajan, 2004 **(E)**	Karnataka (202, 404), Kerala (404)
*Vombisidris occidua* Bolton, 1991 **(E)**	Karnataka (7, 95, 122)
**PONERINAE**	
***Anochetus***	
*Anochetus cryptus* Bharti & Wachkoo, 2013 **(E)**	Himachal Pradesh (44), Jammu & Kashmir (7, 44)
*Anochetus graeffei* Mayr, 1870	Andaman and Nicobar Islands (254), Arunachal Pradesh (1), Assam (1), Goa (7), Gujarat (335, 337, 338, 340, 344), Haryana (7, 122, 261, 316), Himachal Pradesh (1), Jammu & Kashmir (80), Karnataka (7, 179, 260, 261, 287, 335, 337, 340, 352, 362), Kerala (179, 352), Maharashtra (179, 335, 337, 340, 340, 352), Manipur (1), Meghalaya (1), Mizoram (1), Nagaland (1), Orissa (179, 335, 337, 340), Rajasthan (331, 334, 335, 337, 338, 340, 344), Sikkim (1), Tamil Nadu (7, 111, 179, 213, 261, 316, 335, 337, 340, 352), Uttar Pradesh (214), Uttarakhand (1), West Bengal (1)
*Anochetus kanariensis* Forel, 1900 **(E)**	Karnataka (7, 160, 179, 261, 352), Kerala (261), Tamil Nadu (160, 179, 261, 352), West Bengal (352)
*Anochetus madaraszi* Mayr, 1897	Arunachal Pradesh (1), Assam (1), Jammu & Kashmir (1), Karnataka (111, 179), Manipur (1), Mizoram (1), Orissa (111, 179), Sikkim (1), Uttar Pradesh (7, 122), West Bengal (1)
*Anochetus myops* Emery, 1893	Arunachal Pradesh (1), Himachal Pradesh (1), Meghalaya (1), Sikkim (1), Uttarakhand (1), West Bengal (1)
*Anochetus obscurior* Brown, 1978	Karnataka (362), Tamil Nadu (7)
*Anochetus pupulatus* Brown, 1978 **(E)**	Gujarat (1), Karnataka (1), Kerala (7, 111), Maharashtra (1), Tamil Nadu (111)
*Anochetus rufus* (Jerdon, 1851) **(E)**	Tamil Nadu (7, 111, 114, 140, 352), West Bengal (352)
*Anochetus sedilloti* Emery, 1884	Gujarat (111, 179, 338, 340, 344), Karnataka (179), Maharashtra (7, 179, 340), Punjab (1), Rajasthan (340), Tamil Nadu (111, 179, 340, 352)
*Anochetus validus* Bharti & Wachkoo, 2013 **(E)**	Jammu & Kashmir (7, 44)
*Anochetus yerburyi* Forel, 1900	Bihar (7, 122, 214), Goa (7, 122, 214), Karnataka (335, 337, 362), Rajasthan (334, 335, 337)
***Bothroponera***	
*Bothroponera henryi* Donisthorpe, 1942 **(E)**	Goa (411, 412), Karnataka (288), Tamil Nadu (7, 114, 140, 352), West Bengal (352)
*Bothroponera rubiginosa* (Emery, 1889)	Arunachal Pradesh (1), Assam (1), Bihar (214), Jharkhand (214), Maharashtra (160, 352), Manipur (1), Mizoram (1), Sikkim (1), Tamil Nadu (256, 352), West Bengal (1)
*Bothroponera sulcata* (Mayr, 1867)	Andhra Pradesh (352), Arunachal Pradesh (1), Assam (1), Goa (410, 411, 412), Haryana (180), Himachal Pradesh (180, 192), Jammu & Kashmir (80), Karnataka (261, 265, 287), Kerala (261), Madhya Pradesh (261), Maharashtra (180, 194, 261), Manipur (1), Orissa (180), Sikkim (1), Tamil Nadu (7, 180, 256, 289, 352), Tripura (1), West Bengal (1)
*Bothroponera sulcata fossulata* (Forel, 1900) **(E)**	Tamil Nadu (7, 114, 160, 180, 352), West Bengal (114, 352)
*Bothroponera sulcata sulcatotesserinoda* (Forel, 1900) **(E)**	Kerala (160, 180), Tamil Nadu (7, 114, 160, 180, 201), West Bengal (114)
*Bothroponera tesseronoda* (Emery, 1877)	Arunachal Pradesh (1), Assam (1), Goa (1), Himachal Pradesh (180, 192), Karnataka (180, 215, 261, 265, 287, 288, 327), Kerala (180, 261, 352), Maharashtra (180, 194), Mizoram (1), Nagaland (1), Orissa (180), Punjab (79), Sikkim (1), Tamil Nadu (140, 180, 194, 219, 352), Tripura (1), Uttar Pradesh (2, 3, 287, 326, 352, 356), Uttarakhand (1), West Bengal (1)
***Brachyponera***	
*Brachyponera jerdonii* (Forel, 1900)	Arunachal Pradesh (1), Assam (1), Himachal Pradesh (7), Jammu & Kashmir (1), Kerala (114, 180, 352), Maharashtra (180), Manipur (1), Mizoram (1), Nagaland (1), Sikkim (1), Tripura (1), Uttarakhand (1), West Bengal (1)
*Brachyponera luteipes* (Mayr, 1862)	Andaman and Nicobar Islands (160, 189, 217, 254, 345, 356, 383, 393), Arunachal Pradesh (1), Assam (1), Haryana (180), Himachal Pradesh (180, 190, 192), Jammu & Kashmir (80), Karnataka (180, 261, 265, 288, 306), Kerala (180, 206, 352), Madhya Pradesh (180), Maharashtra (180), Manipur (1), Meghalaya (1), Mizoram (1), Nagaland (1), Orissa (415), Punjab (79), Sikkim (1), Tamil Nadu (180, 219), Tripura (1), Uttarakhand (1), West Bengal (1)
*Brachyponera luteipes continentalis* (Karavaiev, 1925) **(E)**	Karnataka (220, 221, 352), West Bengal (352)
*Brachyponera nigrita* (Emery, 1895)	Assam (382), Haryana (408), Meghalaya (249, 355), Punjab (79), Sikkim (160, 249, 355), Uttarakhand (1), West Bengal (7, 132, 180, 192)
*Brachyponera obscurans* (Walker, 1859)	Himachal Pradesh (214), Punjab (214), Uttar Pradesh (214)
*Brachyponera sennaarensis* (Mayr, 1862) **(I)**	Maharashtra (379)
***Buniapone***	
*Buniapone amblyops* (Emery, 1887)	Assam (1), Meghalaya (248, 249, 355), Sikkim (1), Uttarakhand (1)
***Centromyrmex***	
*Centromyrmex feae* (Emery, 1889)	Assam (382), Karnataka (261, 263), Kerala (1), Orissa (415), West Bengal (214, 356)
***Cryptopone***	
*Cryptopone nicobarensis* Forel, 1905 **(E)**	Andaman and Nicobar Islands (160, 191, 392)
*Cryptopone subterranea* Bharti & Wachkoo, 2013 **(E)**	Himachal Pradesh (41), Jammu & Kashmir (7, 41)
*Cryptopone testacea* Emery, 1893	Kerala (140, 352), Tamil Nadu (140), West Bengal (352)
***Diacamma***	
*Diacamma assamense* Emery, 1897	Arunachal Pradesh (1), Assam (1), Karnataka (1), Sikkim (206, 355), Tamil Nadu (1), Tripura (247, 250)
*Diacamma ceylonense* Emery, 1897	Goa (411), Karnataka (10, 362, 365), Kerala (160, 352), Maharashtra (229), Tamil Nadu (352)
*Diacamma cyaneiventre* Andre, 1887	Karnataka (6, 261, 287), Kerala (261, 287, 352), Tamil Nadu (5, 157, 180), West Bengal (352)
*Diacamma indicum* Santschi, 1920	Andaman and Nicobar Islands (7, 114, 160, 189), Arunachal Pradesh (1), Assam (1), Karnataka (7, 368), Manipur (1), Mizoram (1), Sikkim (1), West Bengal (1)
*Diacamma rugosum* (Le Guillou, 1842)	Andaman and Nicobar Islands (117, 254, 257, 352, 355), Arunachal Pradesh (1), Assam (1), Bihar (117), Goa (410, 411, 412), Karnataka (19, 20, 117, 180, 214, 248, 249, 260, 261, 265, 288, 306, 319, 327, 352, 355), Kerala (180, 261, 349, 352), Maharashtra (117, 205, 229, 248, 249, 257, 261, 319, 352, 355, 356), Manipur (1), Meghalaya (1), Mizoram (1), Nagaland (1), Orissa (117, 205, 248, 249, 257, 261, 352, 355, 356), Sikkim (1), Tamil Nadu (256, 289, 352, 355), Tripura (1), West Bengal (1)
*Diacamma rugosum doveri* Mukherjee, 1934 **(E)**	Karnataka (256)
*Diacamma rugosum jerdoni* Forel, 1903	Kerala (114, 140, 352), West Bengal (352)
*Diacamma rugosum rothneyi* Forel, 1900	Kerala (7, 160, 180)
*Diacamma rugosum sculptum* (Jerdon, 1851)	Andaman and Nicobar Islands (206, 355), Arunachal Pradesh (1), Assam (1), Karnataka (180, 206, 352, 355, 356), Kerala (180, 206, 225, 352, 355), Maharashtra (180, 206, 355, 356), Orissa (180), Sikkim (1), Tamil Nadu (206, 352, 355), West Bengal (1)
*Diacamma rugosum sikkimense* Forel, 1903	Sikkim (1)
*Diacamma rugosum viridipurpureum* Emery, 1893	India (no further state, 32)
*Diacamma scalpratum* (Smith, 1858)	Arunachal Pradesh (1), Assam (160, 180, 248, 249, 261, 355, 356), Jammu & Kashmir (114), Karnataka (261), Kerala (225, 261), Meghalaya (1), Sikkim (1), West Bengal (1)
***Ectomomyrmex***	
*Ectomomyrmex annamitus* (Andre, 1892)	Karnataka (287), Kerala (352), Tamil Nadu (1), West Bengal (352)
*Ectomomyrmex annamitus arcuatus* Forel, 1900 **(E)**	Kerala (7, 160, 180), West Bengal (114)
*Ectomomyrmex astutus* (Smith, 1858)	Arunachal Pradesh (206, 382), Assam (1), Meghalaya (1), Sikkim (206, 355)
*Ectomomyrmex javanus* Mayr, 1867	Arunachal Pradesh (1), Assam (1), Meghalaya (228, 249), Sikkim (132, 206, 355), West Bengal (132, 206, 249, 355, 356, 359)
*Ectomomyrmex leeuwenhoeki* (Forel, 1886)	Arunachal Pradesh (206), Assam (7, 114, 160, 172, 206, 247, 248, 249, 250, 287, 352, 355), Karnataka (287), Kerala (160, 206, 247, 249, 250, 352, 355), Meghalaya (206, 248, 249, 355), Sikkim (206, 355), Tripura (247, 250), West Bengal (114, 248, 249, 352)
*Ectomomyrmex striolatus* Donisthorpe, 1933	Himachal Pradesh (7, 135), Uttarakhand (1)
***Emeryopone***	
*Emeryopone narendrani* Varghese, 2006 **(E)**	Karnataka (364)
***Harpegnathos***	
*Harpegnathos saltator* Jerdon, 1851	Assam (114, 199, 261, 352), Goa (411, 412), Karnataka (7, 19, 20, 100, 136, 179, 241, 256, 260, 261, 264, 265, 270, 287, 288, 352, 362), Kerala (114, 136, 160, 179, 261, 305, 329, 352), Maharashtra (1), Punjab (21), Tamil Nadu (256), West Bengal (352)
*Harpegnathos saltator cruentatus* (Smith, 1858)	Karnataka (136, 179), Kerala (136, 179), Maharashtra (136, 179)
*Harpegnathos venator* (Smith, 1858)	Arunachal Pradesh (1), Assam (1), Himachal Pradesh (136, 179, 192), Jammu & Kashmir (80), Manipur (1), Meghalaya (1), Nagaland (1), Punjab (79), Sikkim (1), Tamil Nadu (7, 114, 136, 154, 160, 179, 319, 352, 355, 391), Uttarakhand (1), West Bengal (1)
***Hypoponera***	
*Hypoponera aitkenii* (Forel, 1900) **(E)**	Goa (1), Gujarat (1), Karnataka (7, 78), Kerala (78), Maharashtra (1), Tamil Nadu (78), West Bengal (114, 160, 180)
*Hypoponera assmuthi* (Forel, 1905) **(E)**	Arunachal Pradesh (1), Assam (1), Jammu & Kashmir (78), Karnataka (78), Maharashtra (7, 114, 160, 191), Nagaland (1), Sikkim (1), West Bengal (1)
*Hypoponera confinis* (Roger, 1860)	Arunachal Pradesh (1), Assam (1), Himachal Pradesh (78), Jammu & Kashmir (78, 80), Karnataka (352, 356), Kerala (187, 188), Mizoram (1), Nagaland (1), Punjab (1), Sikkim (1), Uttarakhand (1), West Bengal (1)
*Hypoponera kashmirensis* Bharti, Akbar, Wachkoo & Singh, 2015 **(E)**	Jammu & Kashmir (78)
*Hypoponera ragusai* (Emery, 1894) **(I)**	Arunachal Pradesh (1), Assam (1), Himachal Pradesh (78, 180, 192), Jammu & Kashmir (78), Karnataka (78), Kerala (78), Maharashtra (7, 98, 180, 386, 395), Manipur (1), Mizoram (1), Orissa (180, 395), Punjab (1), Sikkim (1), West Bengal (1)
*Hypoponera schmidti* Bharti, Akbar, Wachkoo & Singh, 2015 **(E)**	Arunachal Pradesh (1), Karnataka (78)
*Hypoponera shattucki* Bharti, Akbar, Wachkoo & Singh, 2015 **(E)**	Arunachal Pradesh (1), Kerala (78)
*Hypoponera truncata* (Smith, 1860)	Arunachal Pradesh (206, 359), Karnataka (359), Sikkim (206, 355), Tamil Nadu (206, 352, 355), West Bengal (206, 352, 355, 356, 359)
*Hypoponera wroughtonii* (Forel, 1900) **(E)**	Arunachal Pradesh (1), Assam (1), Himachal Pradesh (78), Karnataka (7), Sikkim (1), Uttarakhand (1), West Bengal (1)
***Leptogenys***	
*Leptogenys assamensis* Forel, 1900	Assam (1), Meghalaya (1)
*Leptogenys binghamii* Forel, 1900	Assam (1), Karnataka (249), Meghalaya (1)
*Leptogenys birmana* Forel, 1900	Assam (1), Karnataka (1), Kerala (1), Meghalaya (1), Tamil Nadu (7, 213, 352), Tripura (1), West Bengal (249, 352, 356)
*Leptogenys carinata* Donisthorpe, 1943 **(E)**	Kerala (7, 114, 352), West Bengal (352)
*Leptogenys chinensis* (Mayr, 1870)	Arunachal Pradesh (1), Assam (1), Gujarat (237), Jammu & Kashmir (1), Karnataka (7, 180, 261, 265, 287, 306, 362), Kerala (180), Maharashtra (180, 229), Manipur (1), Orissa (180), Tamil Nadu (180, 286), Tripura (1), Uttarakhand (1), West Bengal (170, 180, 299, 300, 356)
*Leptogenys dalyi* Forel, 1900	Karnataka (180, 261, 352), Kerala (180, 261, 352), Tamil Nadu (7, 114, 180, 261, 352), West Bengal (352)
*Leptogenys dentilobis* Forel, 1900 **(E)**	Arunachal Pradesh (1), Assam (1), Gujarat (1), Karnataka (7, 180, 261, 287, 352), Kerala (7, 160, 180, 261, 287, 305, 352), Maharashtra (115, 180, 261, 287, 352), Manipur (1), Mizoram (1), Sikkim (1), Tamil Nadu (180, 261, 287, 352), West Bengal (1)
*Leptogenys diminuta* (Smith, 1857)	Andaman and Nicobar Islands (117, 254), Arunachal Pradesh (1), Assam (1), Goa (410), Jammu & Kashmir (80), Karnataka (261, 265, 288, 306, 352), Kerala (305), Maharashtra (115, 180), Meghalaya (1), Mizoram (1), Nagaland (1), Orissa (180), Sikkim (1, 355), Tamil Nadu (180, 352, 396), West Bengal (1, 355, 356)
*Leptogenys diminuta deceptrix* Forel, 1901	Arunachal Pradesh (1), Assam (1), Maharashtra (7), Sikkim (1), West Bengal (1)
*Leptogenys diminuta diminutolaeviceps* Forel, 1900	Arunachal Pradesh (1), Assam (1), Maharashtra (180), Orissa (180), Sikkim (1), West Bengal (1)
*Leptogenys diminuta laeviceps* (Smith, 1857)	Arunachal Pradesh (1), Assam (1), Himachal Pradesh (180, 192, 214), Jammu & Kashmir (1), Karnataka (260), Maharashtra (180), Punjab (79), Sikkim (1), Uttarakhand (1), West Bengal (1)
*Leptogenys diminuta palliseri* Forel, 1900	Karnataka (7, 114, 160, 352), West Bengal (352)
*Leptogenys diminuta striatula* Emery, 1895	West Bengal (356)
*Leptogenys diminuta woodmasoni* (Forel, 1886) **(E)**	Arunachal Pradesh (1), Assam (1), Sikkim (1), West Bengal (1)
*Leptogenys emiliae* Forel, 1902 **(E)**	Gujarat (7, 114, 160, 187, 188)
*Leptogenys falcigera* Roger, 1861 **(I)**	Kerala (305)
*Leptogenys hysterica* Forel, 1900	Himachal Pradesh (1), Karnataka (180), Uttar Pradesh (214), Uttarakhand (1)
*Leptogenys iridipennis* (Smith, 1858) **(E)**	Sikkim (1), West Bengal (1)
*Leptogenys jeanettei* Mathew & Tiwari, 2000 **(E)**	Meghalaya (1)
*Leptogenys kitteli* (Mayr, 1870)	Arunachal Pradesh (1), Assam (114, 160, 180, 199, 247, 248, 249, 250, 319, 355, 356), Himachal Pradesh (355, 356), Karnataka (287), Kerala (319), Manipur (250), Meghalaya (1), Sikkim (1), Tripura (247, 250), Uttar Pradesh (355, 356), West Bengal (1)
*Leptogenys kitteli minor* Forel, 1900	Sikkim (1), West Bengal (1)
*Leptogenys lattkei* Bharti & Wachkoo, 2013 **(E)**	Himachal Pradesh (7, 43)
*Leptogenys longiscapa* Donisthorpe, 1943 **(E)**	Kerala (7, 114, 352), West Bengal (352)
*Leptogenys lucidula* Emery, 1895	Sikkim (1), Uttarakhand (1), West Bengal (1)
*Leptogenys moelleri* (Bingham, 1903) **(E)**	Sikkim (1)
*Leptogenys mutabilis* (Smith, 1861)	Assam (172)
*Leptogenys peuqueti* (Andre, 1887)	Andaman and Nicobar Islands (254), Karnataka (1), Kerala (1, 180, 305), Meghalaya (1), Sikkim (1), West Bengal (1, 7)
*Leptogenys processionalis* (Jerdon, 1851)	Bihar (214), Chhattisgarh (180), Goa (410, 411, 412), Gujarat (335, 338, 340, 344), Jharkhand (214), Karnataka (7, 180, 260, 261, 264, 265, 287, 288, 306, 333, 335, 340, 362), Kerala (114, 140, 180, 225, 249, 261, 305, 333, 335, 340, 352), Madhya Pradesh (180), Maharashtra (180, 229), Meghalaya (249, 335, 340), Orissa (180, 335), Rajasthan (333, 334, 335, 340), Tamil Nadu (114, 219, 249, 256, 261, 333, 335, 340, 352), Tripura (1), West Bengal (180, 300, 335, 352, 356)
*Leptogenys punctiventris* (Mayr, 1879)	Kerala (1), Meghalaya (1), Sikkim (1), West Bengal (1)
*Leptogenys roberti* Forel, 1900	Arunachal Pradesh (1), Assam (1)
*Leptogenys roberti coonoorensis* Forel, 1900 **(E)**	Kerala (352), Tamil Nadu (7, 180, 352, 356), West Bengal (352, 356)
*Leptogenys stenocheilos* (Jerdon, 1851) **(E)**	Kerala (352), Tamil Nadu (114, 352)
*Leptogenys transitionis* Bharti & Wachkoo, 2013 **(E)**	Himachal Pradesh (7, 43)
*Leptogenys yerburyi* Forel, 1900	Karnataka (180), Kerala (180)
***Mesoponera***	
*Mesoponera manni* (Viehmeyer, 1924)	Maharashtra (180)
*Mesoponera melanaria* (Emery, 1893)	Karnataka (180, 261, 352, 362), Kerala (7, 140, 352), West Bengal (352)
***Myopias***	
*Myopias shivalikensis* Bharti & Wachkoo, 2012 **(E)**	Jammu & Kashmir (7, 38)
***Odontomachus***	
*Odontomachus monticola* Emery, 1892	Andaman and Nicobar Islands (206, 355), Arunachal Pradesh (1), Assam (7, 109, 114, 160, 179, 199, 206, 249, 355, 356, 391, 403), Himachal Pradesh (1), Jammu & Kashmir (80), Meghalaya (1), Sikkim (1), West Bengal (1)
*Odontomachus rixosus* Smith, 1857	Arunachal Pradesh (1), Assam (1), Jammu & Kashmir (80), Meghalaya (1)
*Odontomachus simillimus* Smith, 1858	Andaman and Nicobar Islands (254), Arunachal Pradesh (1), Assam (1), Karnataka (261), Kerala (109, 111, 261), Manipur (1), Meghalaya (1), Nagaland (1), Sikkim (1), West Bengal (1)
***Odontoponera***	
*Odontoponera denticulata* (Smith, 1858)	Andaman and Nicobar Islands (1), Arunachal Pradesh (1), Assam (1), Delhi (1), Haryana (408), Himachal Pradesh (1), Jammu & Kashmir (1), Karnataka (1), Kerala (1), Meghalaya (1), Nagaland (1), Punjab (1), Sikkim (1,172), Uttar Pradesh (1), Uttarakhand (1), West Bengal (1)
***Parvaponera***	
*Parvaponera darwin*ii (Forel, 1893)	Arunachal Pradesh (1), Assam (1), Karnataka (352), Kerala (7, 114, 352), Punjab (1), Sikkim (1), Tamil Nadu (352), West Bengal (1)
*Parvaponera darwinii indica* (Emery, 1899)	India (no state record, 32)
***Platythyrea***	
*Platythyrea nicobarensis* Forel, 1905	Andaman and Nicobar Islands (108, 142, 160, 191)
*Platythyrea parallela* (Smith, 1859)	Andaman and Nicobar Islands (254), Arunachal Pradesh (1), Assam (1), Goa (411), Himachal Pradesh (1), Jammu & Kashmir (1), Karnataka (7, 19, 20, 108, 114, 142, 160, 180, 205, 261, 265, 288, 352, 356, 362), Kerala (7, 108, 114, 142, 160, 180, 261, 352, 385), Sikkim (1), Tamil Nadu (205, 261, 352), Uttarakhand (1), West Bengal (1)
*Platythyrea sagei* Forel, 1900	Himachal Pradesh (7, 108, 180, 192), Karnataka (108, 142, 160, 180, 261, 265, 288, 352), Maharashtra (229), Punjab (142, 160, 261), West Bengal (352)
***Ponera***	
*Ponera indica* Bharti & Wachkoo, 2012 **(E)**	Himachal Pradesh (7, 39), Sikkim (414)
*Ponera paedericera* Zhou, 2001	Arunachal Pradesh (1)
*Ponera sikkimensis* Bharti & Rilta, 2015 **(E)**	Sikkim (414)
*Ponera taylori* Bharti & Wachkoo, 2012 **(E)**	Himachal Pradesh (7, 39), Uttarakhand (1)
***Pseudoneoponera***	
*Pseudoneoponera bispinosa* Smith, 1858	Arunachal Pradesh (1), Assam (1), Himachal Pradesh (180, 192), Jammu & Kashmir (80), Punjab (79), Uttarakhand (1), West Bengal (356)
*Pseudoneoponera rufipes* (Jerdon, 1851)	Andaman and Nicobar Islands (206, 254, 257, 355), Arunachal Pradesh (1), Assam (1), Goa (411), Himachal Pradesh (180, 192), Jammu & Kashmir (80), Karnataka (180, 205, 206, 250, 261, 264, 265, 287, 288, 352, 355, 356, 359), Kerala (205, 206, 250, 261, 352, 355, 356, 359), Maharashtra (180), Manipur (1), Meghalaya (1), Mizoram (1), Nagaland (1), Orissa (180, 205, 206, 355), Punjab (79), Sikkim (1), Tamil Nadu (219), Tripura (247, 250), Uttarakhand (1), West Bengal (1)
**PROCERATIINAE**	
***Discothyrea***	
*Discothyrea periyarensis* Bharti, Akbar & Singh, 2015 **(E)**	Kerala (409)
*Discothyrea sringerensis* Zacharias & Rajan, 2004 **(E)**	Karnataka (405), Kerala (1)
*Discothyrea stumperi* Baroni Urbani, 1977	Assam (1), Sikkim (1)
***Probolomyrmex***	
*Probolomyrmex bidens* Brown, 1975 **(E)**	Tamil Nadu (7, 108, 150)
*Probolomyrmex procne* Brown, 1975 **(E)**	Karnataka (7), Tamil Nadu (7, 108, 150)
***Proceratium***	
*Proceratium williamsi* Mathew & Tiwari, 2000	Arunachal Pradesh (1), Himachal Pradesh (47), Meghalaya (1), Meghalaya (47, 249), Sikkim (1), Uttar Pradesh (1), Uttarakhand (1), West Bengal (1,47)
**PSEUDOMYRMECINAE**	
***Tetraponera***	
*Tetraponera aitkenii* (Forel, 1902)	Andaman and Nicobar Islands (254), Goa (373), Karnataka (114, 124, 189, 248, 249, 262, 265, 288, 306, 306, 352, 362, 373), Kerala (294, 373), Maharashtra (129), Meghalaya (1), Tamil Nadu (286, 352, 373)
*Tetraponera allaborans* (Walker, 1859)	Andaman and Nicobar Islands (254), Arunachal Pradesh (1), Assam (1), Goa (373, 410, 411), Gujarat (188, 338, 340, 344, 373), Haryana (340), Himachal Pradesh (1), Jammu & Kashmir (80), Karnataka (7, 124, 188, 256, 262, 287, 327, 340, 362, 373, 374), Kerala (188, 373), Maharashtra (129, 188, 373), Meghalaya (1), Nagaland (1), Orissa (188, 373), Punjab (79), Sikkim (1), Tamil Nadu (205, 256, 286, 340, 352, 373), Uttarakhand (1), West Bengal (1)
*Tetraponera attenuate* Smith, 1877	Arunachal Pradesh (1), Assam (1), Sikkim (1), West Bengal (7, 373)
*Tetraponera binghami* (Forel, 1902)	Arunachal Pradesh (1), Assam (1), Maharashtra (12, 186, 188), Tamil Nadu (7, 373), West Bengal (356, 373)
*Tetraponera modesta* (Smith, 1860)	Manipur (1)
*Tetraponera nigra* (Jerdon, 1851)	Andhra Pradesh (373), Arunachal Pradesh (1), Assam (1), Delhi (1), Goa (411, 412), Haryana (21), Himachal Pradesh (21, 188, 192, 373), Jammu & Kashmir (80), Karnataka (7, 124, 125, 188, 256, 262, 265, 287, 288, 352, 355, 362, 373), Kerala (7, 114, 186, 188, 188, 262, 287, 352, 355, 356, 373, 374), Madhya Pradesh (373), Maharashtra (7, 114, 115, 188, 262, 352, 355, 356, 373), Meghalaya (1), Nagaland (1), Orissa (373), Punjab (21, 21), Sikkim (1), Tamil Nadu (140, 188, 256, 262, 286, 289, 352, 355, 373), Uttar Pradesh (319, 373), Uttarakhand (1), West Bengal (1)
*Tetraponera nitida* (Smith, 1860)	Andaman and Nicobar Islands (114, 189, 254, 373), Kerala (12, 186, 188, 352, 373), Punjab (21), Tamil Nadu (186), West Bengal (352)
*Tetraponera periyarensis* Bharti & Akbar, 2014 **(E)**	Kerala (64)
*Tetraponera pilosa* (Smith, 1858)	Andaman and Nicobar Islands (114, 189, 254, 373)
*Tetraponera rufonigra* (Jerdon, 1851)	Andaman and Nicobar Islands (114, 189, 254, 373, 385), Arunachal Pradesh (1), Assam (1), Bihar (360), Delhi (1), Goa (373, 410, 411, 412), Gujarat (7, 237, 335, 337, 340, 342, 373), Haryana (21, 335, 337, 340, 342, 373), Himachal Pradesh (21, 335, 337, 340, 342, 373), Jammu & Kashmir (80), Jharkhand (360), Karnataka (7, 205, 206, 256, 262, 265, 287, 288, 306, 335, 337, 340, 342, 352, 357, 362, 373, 374), Kerala (7, 140, 205, 206, 305, 335, 337, 340, 342, 352, 357, 373), Maharashtra (229, 373), Manipur (335, 337, 342, 357), Meghalaya (1), Mizoram (1), Nagaland (1), Orissa (335, 337, 342, 373), Punjab (21, 79, 335, 337, 340, 342), Rajasthan (7, 116, 331, 333, 334, 335, 337, 338, 342, 344, 373), Sikkim (1), Tamil Nadu (7, 140, 205, 219, 256, 286, 289, 335, 337, 340, 342, 352, 357, 373), Tripura (1), Uttar Pradesh (335, 337, 340, 342, 373), Uttarakhand (1), West Bengal (1)

### Dubious records

Several records have been historically reported either from India or from specific states that we have excluded from the list above. Here we briefly present them and explain why those records have been excluded.

**Table:** Dubious records

Taxonomy	State(s) recorded	Explanation
**Amblyoponinae**		
*Myopopone castanea* (Smith, 1860)	Haryana, Punjab	Erroneous locality
**Dolichoderinae**		
*Tapinoma indicum* Forel, 1895	Haryana, Punjab	Misidentification (*Tapinoma melanocephalum*)
**Dorylinae**		
*Aenictus clavatus* Forel, 1901	Haryana	Erroneous locality
*Cerapachys keralensis* Karmaly, 2012	Kerala	Considered species *species inquirenda* (60)
*Cerapachys sulcinodis* Emery, 1889	Haryana, Punjab, Rajasthan	Misidentification (*Cerapachys longitarsus*) and erroneous locality
**Formicinae**		
*Camponotus angusticollis* (Jerdon, 1851)	Haryana	Erroneous locality
*Camponotus arrogans* (Smith, 1858)	Punjab	Erroneous locality
*Camponotus dolendus* Forel, 1892	Himachal Pradesh	Erroneous locality
*Camponotus invidus* Forel, 1892	Himachal Pradesh	Erroneous locality
*Camponotus mitis* (Smith, 1858)	Haryana	Erroneous locality
*Camponotus oblongus* (Smith, 1858)	Himachal Pradesh	Erroneous locality
*Camponotus sericeus* (Fabricius, 1798)	Uttar Pradesh	Misidentification (*Camponotus opaciventris*)
*Camponotus siemsseni* Forel, 1901	Uttar Pradesh	Exact locality not known
*Camponotus wasmanni* Emery, 1893	Jammu & Kashmir, Uttar Pradesh	Misidentification (*Camponotus wasmanni mutilaris*)
*Formica clara* Forel, 1886	Himachal Pradesh, Punjab	Erroneous locality
*Formica fusca* Linnaeus, 1758	Uttar Pradesh	Erroneous locality
*Formica gagates* Latreille, 1798	Haryana	Erroneous locality
*Formica gravelyi* Mukerjee, 1930	West Bengal	Erroneous locality
*Formica rufibarbis* Fabricius, 1793	Sikkim	Misidentification (*Formica fusca*)
*Lasius niger* (Linnaeus, 1758)	Punjab	Erroneous locality
*Polyrhachis exercita rastrata* Emery, 1889	Goa	Misidentification (*Polyrhachis exercita*)
*Polyrhachis jerdonii* Forel, 1894	Punjab	Erroneous locality
*Polyrhachis rupicapra* Roger, 1863	Punjab	Erroneous locality
**Myrmicinae**		
*Aphaenogaster beccarii* Emery, 1887	Haryana, Himachal Pradesh, Punjab	Erroneous locality
*Aphaenogaster feae* Emery, 1889	Goa	Misidentification (*Aphaenogaster baccarii*)
*Aphaenogaster rothneyi* (Forel, 1902)	Maharashtra, Tamil Nadu, Uttar Pradesh	Erroneous locality
*Aphaenogaster sagei* (Forel, 1902)	Haryana, Punjab	Erroneous locality
*Cardiocondyla nuda* (Mayr, 1866)	Arunachal Pradesh, Assam, Manipur, Meghalaya, Mizoram, Nagaland, Sikkim, Tripura, West Bengal	Misidentification (potentially Cardiocondyla kagutsuchi or Cardiocondyla mauritanica, see Seifert 2003)
*Crematogaster buddhae* Forel, 1902	Haryana	Erroneous locality
*Crematogaster walshi* Forel, 1902	Haryana, Himachal Pradesh, Punjab, Rajasthan, Uttar Pradesh	Erroneous locality
*Meranoplus rothneyi* Forel, 1902	Haryana, Himachal Pradesh, Punjab	Erroneous locality
*Messor himalayanus* (Forel, 1902)	Haryana, Uttar Pradesh	Misidentification (*Messor instabilis*)
*Monomorium dichroum* Forel, 1902	Punjab	Erroneous locality
*Monomorium longi* Forel, 1902	Haryana, Punjab	Erroneous locality
*Monomorium monomorium* Bolton, 1987		The status of this species is uncertain and needs extensive taxonomic work. Here we tentatively considered the species valid in the species list of India, but future work might change its status.
*Monomorium orientale* Mayr, 1879	Punjab	Erroneous locality
*Myrmica pachei* Forel, 1906	Jammu & Kashmir	Erroneous locality
*Pheidole lamellinoda* Forel, 1902	Haryana	Erroneous locality
*Solenopsis invicta* Buren, 1972		Misidentification (*Solenopsis geminata*)
*Temnothorax rothneyi* Forel, 1902	Punjab, Uttar Pradesh	Erroneous locality
*Tetramorium caespitum* (Linnaeus, 1758)	Himachal Pradesh	Erroneous locality
*Tetramorium christiei* Forel, 1902	Haryana	Erroneous locality
**Ponerinae**		
*Harpegnathos venator* (Smith, 1858)	Uttar Pradesh	Erroneous locality
*Leptogenys dalyi* Forel, 1900	Punjab	Erroneous locality
*Leptogenys dentilobis* Forel, 1900	Punjab	Erroneous locality
*Odontomachus haematodus* (Linnaeus, 1758)	Arunachal Pradesh, Assam, Goa, Karnataka, Kerala, Maharashtra, Manipur, Meghalaya, Nagaland, Sikkim, Tamil Nadu, West Bengal	Misidentification (potentially *Odontomachus simillimus*)
*Ponera affinis* Heer, 1849	Kerala	*incertae sedis* in *Ponera*
**Pseudomyrmecin**ae		
*Tetraponera carbonaria* (Smith, 1863)	West Bengal	Erroneous locality
